# New RNA Structural Elements Identified in the Coding Region of the Coxsackie B3 Virus Genome

**DOI:** 10.3390/v12111232

**Published:** 2020-10-30

**Authors:** Mariola Dutkiewicz, Jakub Kuczynski, Michal Jarzab, Aleksandra Stachowiak, Agata Swiatkowska

**Affiliations:** Institute of Bioorganic Chemistry Polish Academy of Sciences, Zygmunta Noskowskiego 12/14, 61-704 Poznań, Poland; jakkuc23@gmail.com (J.K.); m.p.jarzab@gmail.com (M.J.); aleksandra.stachowiak1@gmail.com (A.S.); agaswiat@ibch.poznan.pl (A.S.)

**Keywords:** enterovirus, Coxsackie B3 virus, coxsackievirus B3, CVB3, RNA secondary structure, RNA motif, RNA structural element, RNA structure of coding region, RNA virus, RNA–protein interaction

## Abstract

Here we present a set of new structural elements formed within the open reading frame of the virus, which are highly probable, evolutionarily conserved and may interact with host proteins. This work focused on the coding regions of the CVB3 genome (particularly the V4-, V1-, 2C-, and 3D-coding regions), which, with the exception of the *cis*-acting replication element (CRE), have not yet been subjected to experimental analysis of their structures. The SHAPE technique, chemical modification with DMS and RNA cleavage with Pb^2+^, were performed in order to characterize the RNA structure. The experimental results were used to improve the computer prediction of the structural models, whereas a phylogenetic analysis was performed to check universality of the newly identified structural elements for twenty CVB3 genomes and 11 other enteroviruses. Some of the RNA motifs turned out to be conserved among different enteroviruses. We also observed that the 3′-terminal region of the genome tends to dimerize in a magnesium concentration-dependent manner. RNA affinity chromatography was used to confirm RNA–protein interactions hypothesized by database searches, leading to the discovery of several interactions, which may be important for virus propagation.

## 1. Introduction

Over the last decade the importance of the secondary structure of the RNA of viral genomes has become apparent, which has led to the mapping of the RNA of whole viral genomes, and transcriptomes isolated from simple organisms [1,2,3,4,5]. There is growing evidence for the presence of functional RNA motifs in protein coding sequences. Protein-coding RNA sequences can also fold into functional structural motifs, similar to those found in non-protein-coding RNA sequences, though it appears to be less common [1,6].

**Figure 1:**
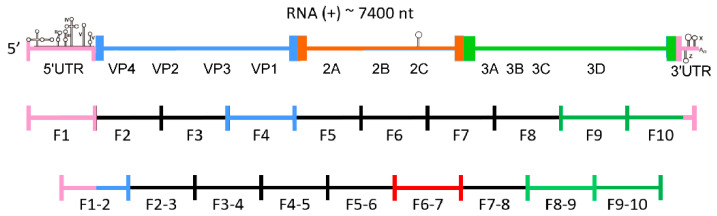
Scheme of coxsackievirus B3 genome organization with division into fragments, ca. 750 nt each. Fragments examined in this study are highlighted with colors corresponding to respective regions of the viral genome; UTR—untranslated region; VP1, VP2, VP3, VP4—capsid proteins; 2A, 2B, 2C, 3A, 3B, 3C, 3D—non-structural proteins.

By using global analysis of viral genomes in search of secondary structures, it was possible to identify new structural patterns and motifs present in both the coding and non-coding regions [7,8]. Numerous structures present in viral genomes play important roles, including at the stages of replication, translation, and stabilization of RNA transcripts or translocation. In the case of HIV (human immunodeficiency virus), analysis using SHAPE (Selective 2′ Hydroxyl acylation Analyzed by Primer Extension) showed that both terminal regions—the 5′-UTR and the 3′-UTR—are characterized by high complexity structures, while the coding region only contains periodically occurring complex structures and relatively unstructured regions [7]. The complex structures in the coding region are periodic in nature and are important during translation, where they contribute to slowing down the ribosome, which in turn affects the process of protein folding. Repeated structural patterns present near the regions responsible for coding particular protein domains have been identified in HIV [1]. Stopping or slowing down ribosomes with the higher order mRNA structure can facilitate the correct folding of proteins. This in turn suggests that the protein structure can be influenced by both the primary sequence and the higher-order RNA structures. An example is the cellular ribonucleoprotein, within the structure of the SP (signal peptide) particle, and this probably gives additional time for recognition and binding of SRP and subsequent translocation of the complex to the endoplasmic reticulum [9]. Another good example is the hepatitis C virus (HCV), which has a number of conserved structures in its genome that are necessary for its proper and efficient replication [10]. Evolutionarily conserved structures have also been found in the ORF, primarily within the regions encoding core protein (C) and the viral RNA polymerase (NS5B; Figure 1). The key structural element for virus replication is 5BSL3.2, identified in the NS5B gene [10]. The complementary sequence that creates the long-range kissing loop interactions is located in the SL2 motif located in the 3′-UTR region. Another site of long-range interaction is found in the 5′-UTR [10,11]. These examples of the genome structures of RNA viruses (HIV, HCV) show that coding regions of the RNA viral genomes often contain structural motifs of very important functional significance, in addition to coding for proteins.

The degree of RNA structure complexity in the protein coding regions can affect translation by changing the reading frame with a pseudoknot or temporarily stalling the ribosome, for example. Identification of thermodynamically stable and evolutionarily conserved RNA secondary structures within Epstein–Barr Virus (EBV) coding regions revealed the importance of their function in the viral life cycle. Analysis of the structure of the EBV genome led to the tentative identification of twenty-two structures, which include the START and STOP codons. RNA structure has been shown to influence the regulation of translation by modulating the accessibility of these codons. A high degree of RNA structure in the vicinity of the STOP codon can lead to “readthrough”, or continuation of translation past a stop codon. In contrast, manipulation of the START codon can result in a reduction in the efficiency of translation [3].

The GORS (genome-scale ordered RNA structures) occurrence prediction for the genus *Enterovirus* of the *Picornaviridae* family showed only two potential secondary structure motifs located inside the open reading frame [12]. One of them is consistent with the well-known CRE (*cis*-acting replication element) also called oriI (internal origin of replication). It folds into a stem-loop RNA structure within the 2C-ATPase coding site of poliovirus (PV), coxsackievirus B3, and other members of the genus and it plays a crucial role during the replication process [13,14]. The second predicted structural motif is localized in the 3D site encoding RNA-dependent RNA polymerase. The Viral RNA Structure Database used a phylogenetic-structural computer analysis to propose a new structural element: the E-10 hairpin, found in a number of enteroviruses and potentially conserved in all of them [12]. Later, the presence of E-10 was experimentally demonstrated in poliovirus, in the wider context of a WT-β hairpin [12,15]. Another RNA element was proposed in the same publication: the WT-α element, which is located slightly upstream of the WTβ element. Both RNA structures proved to be important for the replication efficacy. Burrill et al. investigated the characteristics of the RNA structure on the PV genome scale using the SHAPE method, finding a very interesting RNA element, 3D-7000, which largely overlaps with the sequence of WT-α [5]. Various models regarding their construction were proposed: WT-α/3D-7000 [5,15]. Their function has yet to be determined in detail, but they influence replication efficiency. The presence of the E-10 hairpin in the CVB3 genome has not yet been demonstrated experimentally.

In contrast, a new extended structural element was found experimentally that had not previously been predicted by bioinformatic screening. In the 3′-terminal of the type C enteroviruses, which includes poliovirus, a complex RNA structure has been found that inhibits ribonuclease L: i-RNase L. This element is encoded in the 3CPro site of poliovirus and several types of coxsackievirus A, all members of the subgenus Enterovirus C [16,17,18]. This discovery showed how important it is to not only to search for universal RNA structures among viral families and genera but also to screen smaller phylogenetic groups for less broadly conserved structural elements as well. For enteroviruses, more than 100 serotypes have been defined so far, which include: 4 poliovirus, 23 coxsackievirus A, and 6 coxsackievirus B serotypes [19]. It seems probable that important structural elements common to different strains and isolates within a given serotype exist.

CVB3 and another ssRNA(+) virus, Hepatitis C virus (HCV) are sensitive to RNase L digestion, and they do not have the known i-RNase L structure within their genomes. It has been shown that fragments generated by this enzyme from the HCV genome are able to refold into hairpin structures, inducing an interferon response. Among them are so called “super-inductors” of interferon, which is probably a function of their secondary structure [20].

The genome of coxsackievirus B3 (CVB3), a member of the *Picornaviridae* family, consists of positive-sense single-stranded RNA molecule, 7400 nucleotides (nt) long [21]. It contains one open reading frame, flanked by two untranslated regulatory regions, the secondary RNA structures of which have been thoroughly studied [22,23,24]. However, the intervening coding region remains uncharacterized, with the exception of the CRE, which is indispensable for viral replication [14]. The known RNA structural elements, such as the 5′-cloverleaf, IRES, CRE, and the 3′-terminal pseudoknot, play important roles in the life cycle of the virus. Little is known about RNA structure elements present within the coding region of CVB3.

In this study, the goal was to find very well-defined and evolutionarily conserved RNA structural elements in the CVB3 genome, particularly in its protein-coding region. We consider well-defined structural elements as those that remain unchanged in alternate predictions of the structure, and therefore have a high probability of occurrence in vivo. The selected regions of CVB3 genome were subjected to secondary structure probing, and to phylogenetic analysis. We focused on the V4-, V1-, 2C-, and 3D-coding regions. By analogy to other RNA viruses, we expected to find structurally well-defined and potentially essential RNA elements, especially in the highly conserved sequence encoding viral polymerase (3D). Additionally, we investigated the 3D-coding region for RNase L digestion sites, the homodimerization process, and RNA–protein interactions.

## 2. Materials and Methods

### 2.1. Materials

The materials used in this study were from the following sources: Unlabeled DNA primers were from Genomed (Warsaw, Poland). Fluorescently 5′-labeled “reverse” primers were from Life Technologies (ThermoFisher Scientific, Waltham, MA, USA) and all the chemicals (DMS, DMSO, EDTA dithiothreitol, lead acetate, Tris, KCl, MgCl_2_, ATP, β-mercaptoethanol) were from Sigma-Aldrich Corp (St. Louis, MO, USA) or BioShop Canada Inc. (Burlington, Ontario, Canada). Enzymes: Q5 DNA polymerase (New England Biolabs, Ipswich, MA, USA), Superscript III reverse transcriptase (Life Technologies), recombinant RNase L (in an active state) were purchased from Cusabio Technology LLC (Houston, TX, USA).

### 2.2. DNA Templates and RNA Synthesis

In order to obtain the dsDNA templates for fragments F1, F1–2, F4, F6–7, F8–9, F9, F9–10, and F10, under control of the T7 promoter, single-stranded DNA complementary to the 3′ end of the CVB-3 genome was synthesized by RT-PCR using total RNA isolated from HeLa cells infected with the “Nancy” strain of CVB-3. To this end, the Superscript III reverse transcriptase and 10+(R) were used. The cDNAs were amplified by PCR with the primers shown in Table 1. The dsDNA templates were amplified with the Q5-MasterMix (New England Biolabs) according to the manufacturer’s protocols. Synthesis of dsDNA templates for F1-F10 was carried out in one step. The forward primer included the promotor sequence for the T7 polymerase. As a result, eight dsDNAs encoding the desired RNA sequences were generated, each containing the T7 promoter at the 5′ end. The reaction products were purified using the PCR/DNA Clean Up Kit (EURx, Gdansk, Poland) and the obtained dsDNA templates were dissolved in TE buffer.

In the next step, the ca. 750-nt long RNAs were transcribed. Transcription reactions were performed using the TranscriptAid T7 High Yield Transcription Kit (Thermo Fisher Scientific) according to the manufacturer’s instructions. In case of RT products intended for capillary electrophoresis, 5′-end fluorescently labeled primers were purchased and added to the reverse transcription reaction and incubated at 55 °C for 10 min. The synthesized RNAs, were checked for size, integrity, and homogeneity on a denaturing agarose gel, and purified with the GeneJet RNA Cleanup and Concentration Micro Kit (Thermo Fisher Scientific). The quantity of RNA obtained was measured spectrophotometrically at λ = 260 nm.

### 2.3. RNA Structure Probing In Vitro

The in vitro SHAPE (Selective 2′-Hydroxyl Acylation analyzed by Primer Extension) reaction, RNA cleavage in the presence of lead ions (Pb^2+^), and DMS methylation were performed on the basis of protocols published elsewhere [25,26,27,28]. Briefly, after standard RNA renaturation, the SHAPE reagent 2-methylnicotinic acid imidazolide (NMIA, NAI; Merck KGaA, Darmstadt, Germany) was added to the sample at a final concentration of 55 mM. One control sample was treated with DMSO only (Sigma-Aldrich). After incubation for 50 min at 37 °C, the samples were subjected to RNA precipitation. For a second probing assay, 8.3% DMS (Sigma-Aldrich) solution in ethanol was added to two samples to a final concentration of 0.4%. The second control sample was only treated with ethanol. After incubation for 7 or 14 min at 37 °C, the samples were treated with ice-cold 100 mM dithiothreitol (Sigma-Aldrich) to stop the reaction and subjected to RNA precipitation. Lead acetate (Sigma-Aldrich) was added to three samples at a final concentration of 0.5, 1, or 2 mM. An additional control sample was treated with just water. After incubation for 5 min at 37 °C, the samples were treated with chilled 100 mM EDTA (Sigma-Aldrich) to stop the reaction and subjected to RNA precipitation. The obtained purified RNA was suspended in RNase-free water.

To determine which sites in the RNA were modified by DMS or NAI treatment or cleaved in the presence of 1 mM lead ions (Pb^2+^), reverse transcription reactions were performed with 0.2 μg of the RNA and a suitable reverse DNA primer (see Table 1) fluorescently labeled at its 5′ end with VIC, FAM, PET, or NED (Life Technologies). To determine the exact cleavage and chemical modification sites, products of the primer extension reaction were run on capillary electrophoresis using ABI 3130 xl sequencer (Applied Biosystems, Thermo Fischer Scientific) along with dideoxy sequencing markers in the Laboratory of Molecular Biology Techniques, Faculty of Biology, Adam Mickiewicz University in Poznań. Dideoxy sequencing markers were generated as described in [27].

### 2.4. Sequences of CVB-3 Isolates

Sequence data were obtained from the National Center for Biotechnology Information (NCBI) [29]. For prediction of conserved RNA motifs, a set of twenty full CVB-3 and 11 other enteroviruses genome sequences from human strains was used, Taxonomy IDs: M88483.1; M16572.1; AY752946.1; AY752945.1; AY752944; AF231765.1; AF231763.1; AF231764.1; M33854.1; JX312064.1; U57056.1; FJ000001.1; GQ141875.1; GU109481.1; FJ357838.1; EU144042.1; AY673831.1; JN048468.1; JQ040513.1; JN048469.1. Other enteroviruses: U05876.1, M16560.1, AF085363.1, X05690.1, X67706.1, D00538.1, X84981.1, AF176044.1, V01150.1, X00595.1, K01392.1.

### 2.5. Secondary Structure Modeling

Based on experimental data and theoretical thermodynamic calculations, the program generated models of analyzed secondary RNA structures. The resulting reactivity values of individual nucleotides from SHAPE reactions were analyzed in the ShapeFinder program, normalized, and then entered into the RNAstructure tool, together with the sequence of the analyzed RNA fragment [30]. The secondary structure models for the analysis of regions of the CVB-3 genome were built with RNAstructure v.5.7 [31] using default options of the program. RNAstructure generates multiple potential models with different free energies. The selected models had a free energy within of 10% of the minimal energy. There is a possibility to incorporate RNA structure probing data, such as from SHAPE experiments, which is what we did, to improve the accuracy of structural predictions [30,32]. The probability reflects the odds of being in the unspecified pair, for a paired nucleotide, or of being single-stranded, for an unpaired nucleotide. The predicted secondary structures were also color annotated with these probabilities. The higher the probability, the higher the confidence of the prediction accuracy. For modeling of the F10 RNA fragment, results from DMS modification and Pb^2+^-induced cleavages were taken as additional information by choosing one of the models generated by the computer program [26,28,33,34].

**Figure 2:**
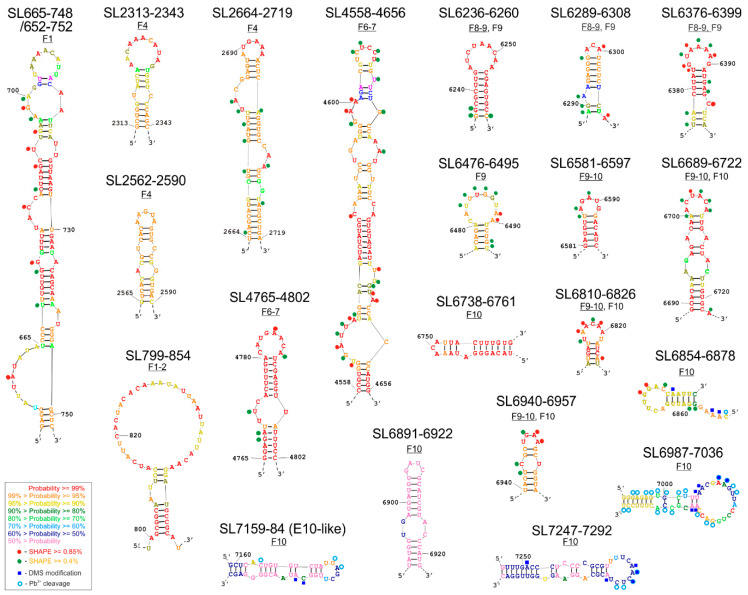
New RNA structural elements identified in the coding part of the Coxsackie B3 virus genome. Most probable secondary structural models of 21 new motifs with experimental mapping results depicted according to the key in the figure. The color of the letters corresponds to the probability values calculated by RNAstructure, according to the key in the figure. Motifs marked in red, orange, and yellow have the highest probability of occurrence in the analyzed RNA fragment and in the corresponding region of the viral genome, so they are considered structurally well-defined. Predicting secondary structure of partly overlapping fragments: F1 and F1–2.

The fragments F1, F1–2, and F10 are known to contain complex autonomous structures, which were omitted for better structure modeling of the remainder of the sequences. For F1 and F1–2, the excluded domain was a part of the IRES elements [22]. In the case of F10 it was a 3′-terminal pseudoknot [23]. In each analyzed fragment, the well-defined, highly probable structural elements were identified. The RNAstructure program analyzes the probability of a particular structural motif being formed, which greatly aided the identification of the conserved elements. The analysis is based on the probability of each nucleotide position being double- or single-stranded in the different structural models of a RNA fragment. In Figure 2 (and SM1) this probability is depicted by the different colors of the nucleotides, according to the legend.

### 2.6. Prediction of Conserved Structural Regions

**Figure 3:**
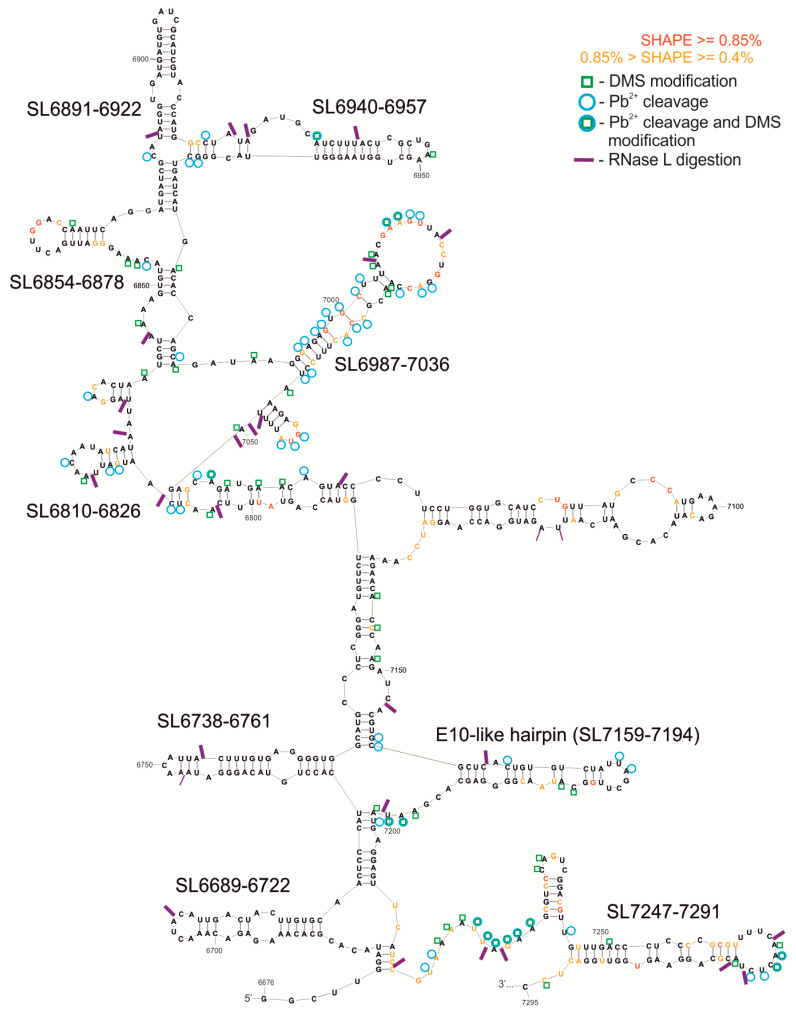
Secondary structure model of F10 RNA, without the last 120 nt of the known pseudoknot. The free energy of the structure is −286.4 kcal/mol. This model displays NMIA modification sites from the SHAPE reaction, DMS modification sites, RNase L digestion, and Pb^2+^ cleavages, according to the key. In addition, the most interesting structural motifs are marked with their names.

The RNA or cDNA sequences of twenty CVB-3 isolates were aligned with sequences of analyzed structural elements in the Clustal Omega program [35,36] using the default RNA parameters. The program aligns two or more sequences and highlights areas of similarity, which may be associated with specific features. The aligned sequences were input into RNAalifold [37,38,39] and run with the default program parameters. RNAalifold predicts the consensus secondary structure for a set of related aligned sequences using a combination of free energy and a covariance (Vienna Package ver. 2.0). The program also calculates the “partition function” and “base pairing probability matrix”. The chosen output is an alignment and graphical representation of a secondary structural model with color annotation of its structural conservation and (Figure 3 and Figure S2) [34].

The Clustal Omega program can be found at https://www.ebi.ac.uk/Tools/msa/clustalo/.

The RNAalifold program can be found at: http://rna.tbi.univie.ac.at/cgi-bin/RNAWebSuite/RNAalifold.cgi.

### 2.7. Digestion with Ribonuclease L (RNase L)

Before the procedure, 5 µg of the F10 RNA was combined with a cleavage buffer containing 25 mM Tris pH 7.4, 100 mM KCl, 10 mM MgCl_2_, 50 µM ATP, and 7 mM β-mercaptoethanol. Four identical samples, each of 14 µL, were prepared, denatured for 5 min at 70 °C, and renatured by incubating for 10 min at 37 °C. Subsequently either 0, 0.5, 1, and 2 µL of 4.15 mM RNase L was added and filled with water to bring the volume to 16 µL. The mixture was incubated for 1 h at 37 °C and the RNA was subsequently purified with the GeneJET RNA Cleanup and Concentration Micro Kit (Thermo Fisher Scientific, Waltham, MA, USA).

### 2.8. Dimerization Studies

From 0.5 to 1 µg of the F10 RNA (for each reaction option) were combined with a cleavage buffer containing 10 or 25 mM Tris pH 7.4, 100 mM KCl or 40 mM NaCl, and 0.5 or 10 mM MgCl_2_. The mixture was prepared in 10 µL samples, denatured for 5 min at 70 °C and renatured by incubating for 10 min at 37 °C. For dimer formation inhibition, either 5- or 50-fold molar excess of DNA oligomer “antydimer” (5′-ATCTA TAGGC CATGG GTACG ATGC GATCAC-3′) was added before renaturation. RNA was subsequently loaded onto a 1% agarose gel with standard non-denaturing 6 × loading dye and subjected to electrophoresis in 0.5 × TAE buffer containing 0.1 mM EDTA. As an RNA length marker, RNA Riboruler was used (Thermo Fisher Scientific).

### 2.9. Predicting RNA Interactions with Proteins Using the ATtRACT Database

Potential interactions with proteins were proposed with the help of the ATtRACT database, which is a database of experimentally validated RNA binding proteins and associated motifs [40]. We uploaded a text file containing RNA sequence(s) in fasta or multi-fasta format and scanned the sequence(s) searching for the presence of motifs. Results are provided in the table format and graphical format. Searches were limited to protein interactions known for *Homo sapiens*. The database can be found on: https://attract.cnic.es/index#.

### 2.10. Cell Culture and Cytoplasmic Lysate Preparation

MCF-7 cells (originally from ECACC, Salisbury, UK) were maintained in DMEM medium. All medium solutions were supplemented with 10% fetal bovine serum, non-essential amino acids (Gibco-BRL, Thermo Fischer Scientific), 100 U/mL of penicillin G, 0.1 mg/mL of streptomycin sulphate (Sigma-Aldrich, St. Louis, MO, USA) and the cells were maintained at 37 °C in a 5% carbon dioxide atmosphere. Genotoxic stress was generated by addition of doxorubicin to a final concentration of 0.5 μg/mL. Approximately 4 × 10^7^ MCF7 cells were used per extract for one RNA-affinity chromatography procedure. The cells were washed with PBS buffer and then they were collected by centrifugation at 1000 rpm. The pellet was resuspended in 5 pellet volumes of CE buffer (10 mM HEPES, 60 mM KCl, 1 mM EDTA, 0.075% (*v*/*v*) NP40, 1 mM DTT, and 1 mM PMSF, pH 7.6) and incubated on ice for 3 min. Then cell lysate was centrifuged at 1000–1500 rpm for 4 min and the cytoplasmic extract was collected.

### 2.11. RNase-Assisted RNA Chromatography

RNase-assisted RNA chromatography was performed as described previously [41,42]. Briefly, F10 RNA (1.3 nmol) was incubated in a 200 μL reaction volume containing 0.1 M NaOAc (pH 5.0) and 5 mM sodium m-periodate (Sigma-Aldrich) for 1 h in the dark at room temperature. After ethanol precipitation, RNA was resuspended in 0.1 M NaOAc (pH 5.0). Adipic acid dehydrazide agarose bead slurry (Sigma-Aldrich) was also resuspended in 0.1 M NaOAc (pH 5.0) and added to the periodate-treated RNA. RNA with the beads was incubated overnight at 4 °C and then the beads were washed three times in 1 mL of 2 M KCl and three times in 1 mL of buffer D (20 mM Tris-HCl, pH 7.9, 20% (*p*/*v*) glycerol, 0.1 M KCl, 0.2 mM EDTA, 0.5 mM dithiothreitol, 0.2 mM PMSF). The RNA coupled to beads was incubated with 40% (*v*/*v*) cytoplasmic extract with addition of 1.5 mM MgCl_2_, 25 mM creatine-phosphate, and 5 mM ATP for 30 min at 37 °C with shaking at 400 rpm. Next, the beads were washed four times with 1 mL of buffer D containing 1.5 mM MgCl_2_ and twice with Milli-Q water. Subsequently, RNA coupled to the beads was incubated in 60 μL reaction mixture containing 10 mM Tris-HCl (pH 7.2), 1 mM MgCl_2_, 40 mM NaCl, and 5 μL of A/T1 ribonuclease mix (Ambion, Thermo Fischer Scientific) for 30 min at 37 °C, shaking at 1400 rpm for 10 sec every minute. The concentration of RNases in the A/T1 mix was 500 U/mL for RNase A and 20,000 U/mL for RNase T1. The resulting reaction mixture was centrifuged for 1 min at 4 °C and the supernatant was collected.

### 2.12. Mass Spectrometry Analysis

The proteins obtained after RNase-assisted RNA chromatography were identified using MS/MS analysis [43]. MS analysis was performed by LC–MS in the Laboratory of Mass Spectrometry (IBB PAS, Warsaw). The peak lists were uploaded to the Mascot engine (version 2.4.1, Matrix Science, Boston, MA, USA) and searched against the SwissProt *Homo sapiens* database. Subsequently, the Mascot results list including proteins represented by at least one peptide with a score above the threshold was analyzed by using MScan software available at proteom.ibb.waw.pl/mscan/index.html.

## 3. Results and Discussion

Due to the extreme evolutionary pressure viruses are subjected to, a conservation of RNA structural motifs within their open reading frames is generally an indication that they play an important role in the life cycle of the virus. For example, they may be sites of interaction with host proteins, as observed with poliovirus and Coxsackie A viruses, where an RNA structural element has recently been demonstrated to bind to cellular ribonuclease L and inhibits its activity. Stable RNA secondary structures in the coding part may also affect the proper folding of viral proteins by slowing the ribosome, as proposed for HIV [1], or play a role in an immune response against HCV [20].

Coxsackie B3 virus, as an enterovirus and representative of the picornavirus family, is an excellent model for research on the occurrence of structural motifs throughout the entire genome and for discovering their function. Moreover, in a broader sense, CVB3 is a representative of viruses with an RNA genome.

To the best of our knowledge, with the exception of the CRE in the 2C region, the coding part of the CVB3 genome has not yet been structurally characterized. We decided to search the coding region of the viral genome for the presence of other RNA structural elements that are highly probable, evolutionarily conserved, and thus potentially important for the virus propagation.

In order to investigate the structure of the entire genome of the CVB3 virus, especially the part encoding the viral polyprotein, nineteen RNA fragments corresponding to the entire viral genome were synthesized. We decided to experimentally study the secondary structure of several of those RNA fragments, each less than or equal to 800 nt in length (Figure 1). This length was proposed as the maximum length allowing proper folding of RNA in vitro, without the involvement of proteins and apart from the transcription process. The minimum number of such fragments required to cover the entire 7400 nt viral genome is 10, but we employed 9 additional fragments in order to be able to observe motifs formed by sequences split in two in the minimal set of 10 fragments. We conducted RNA structure probing using the SHAPE technique (Selective 2′-Hydroxyl Acylation analyzed by Primer Extension) of several RNA fragments, F1, F1–2, F4, F6–7, F8–9, F-9, F9–10, and F10, which correspond, in part, to the regions of CVB3 genome encoding for the V4, V1, 2C, and 3D proteins, respectively (Figure 1) [25]. The secondary structure of the most 3′-terminal region of the CVB3 genome, represented by F10 RNA, was additionally characterized by chemical modification with the DMS and RNA cleavage method in the presence of lead ions (Pb^2+^) [26,33].

These particular fragments of CVB3 genomic RNA were selected for their location in different regions of the genome, representing both conserved non-coding regions (F1, partially F1–2, and F10), coding for variable structural proteins (F1–2 and F4), and conserved non-structural proteins: viral protease 2C (F6–7), and 3D polymerase (F8–9, F9, F9–10, and F10). The selection of the F8–9_ F10 fragments was partly dictated by the relatively high level of conservation of the sequences encoding this polymerase within related viruses. This offered the chance of finding potentially relevant motifs in more than one isolate of RNA virus. The 2C-coding region was chosen in order to test our experimental approach by confirming the presence of the CRE and to search for other structures in the close neighborhood of CRE. Region 3D was chosen because in analogous regions of related viruses, secondary structure elements have already been found, such as: iRNasel (group C enteroviruses), E-10 hairpin (bioinformatic predictions for enteroviruses), and the 3D-7000/WT-α and WT-β elements important for the replication of poliovirus. The V4 region was selected for its proximity to the 5′-UTR. In other viral genomes, such as HCV, stable structures have been found in this position. The V1 region, in contrast, was chosen as the least conserved of all protein coding regions in the CVB3 genome. We were interested to see if there is any difference in abundance of structurally well-defined RNA elements in comparison to the most conserved regions. Another objective was to find strictly CVB3-specific RNA structures in the V1 coding part.

### 3.1. Modeling of RNA Secondary Structures of RNA Fragments and Searching for the Most Probable Elements, which Remain Unchanged in Multiple Structure Predictions

The structural characterization of selected CVB3 genome fragments was performed in vitro using the SHAPE technique. The obtained experimental data were used as constraints in the process of generating the eight RNA secondary structure models (SM1). The RNAstructure program proposed a set of alternative structural models for each fragment, which lie within 10% of the minimal energy. By comparative analysis of those alternative structure models, the probability of the formation of particular RNA motifs was calculated. Based on such comparisons and calculations, 21 structural motifs were identified within 8 RNA fragments (Figure 2). Their probability of occurrence and forming distinct, relatively stable structures of high probability in the corresponding regions of the viral genome are color-coded in the figure.

Here we present a set of individual structures of newly identified, high probability RNA elements (Figure 2), and the secondary structure models of extended RNA fragments, in which they were found (Figure 3 and Figure S1).

#### 3.1.1. Secondary Structure of Partly Overlapping Fragments: F1 and F1–2

Structure of fragments F1 and F1–2 were analyzed on the basis of the experimental results using the RNAstructure program in two ways. First, the full-length sequence of the fragment was analyzed, and then part of the sequences responsible for coding the I–V domains, mostly belonging to the IRES elements of the 5′-UTR region, were removed. This facilitated the calculation of the secondary structures of the 5′-terminal part of the 5′-UTR region, because the IRES region probably does not interact with the rest of the CVB3 genome, as it is believed to be autonomous. As expected, the presence of a previously described structure, an IRES domain VI-VII, was confirmed in our analysis.

The F1 fragments with and without the IRES region were 801 and 221 nt in length, respectively. Close to the 3′-end of the fragment, the program proposed a hairpin-type structure with one single-nucleotide bulge and four several-nt bulges, ending with a 9 nt apical loop rich in adenosines named SL665-748 (Figure 2 and Figure S1A). In addition, this structure, when extended by a few nucleotides on each side, to form SL652-752 contains an asymmetric inner loop/large bulge rich in potential protein interaction sites. A structural element similar to SL665–748 has previously been proposed on the basis of thermodynamic calculations and is referred to as the K-domain (nts: 688-741) [44]. The reactivity values for individual nucleotides obtained in the SHAPE experiment and incorporated into the RNAstructure program helped to confirm the presence of the K-domain in most CVB3 genomes. Interestingly, the location of the start codon, AUG, is very similar to that of the *TP53* transcript, in that it occurs partly in a bulge of the longer stem, and the second and third letters of the codon remain paired [27,45,46,47]. At this stage it is difficult to draw conclusions from such similarity, but the observation itself seems to be worth noting.

It is also important to emphasize the existence of the SL665-748/652-752 motif in the structure projections for the two overlapping RNA molecules: F1 and F1–2, albeit with slight differences in the apical part and probability calculations. The length of the analyzed fragment F1–2 was 765 nt for the option with the IRES and 585 nt without the I–V domains. For the purpose of the analysis of the individual motifs, a structure with a minimal free energy ΔG = −202.3 (without the domains I–V IRES) was selected (Figure S1B). In both proposals, with or without the IRES domains, the program generated a hairpin motif SL799-854, and confirming the presence and structure of an IRES domain VI-VII, familiar from earlier IRES models [22,48]. Motif SL799–854 is characterized by the occurrence of a large apical loop, a stable stem made up of five repetitions of the G–C nucleotide pair and a single bulge made up of two nucleotides (Figure 2, SM1B). The whole element was characterized by very high probability values 95%, which means that the element appeared in 95% of the RNA structures proposed by the program was found to be within 10% of the minimal free energy.

#### 3.1.2. Secondary Structure of Fragment F4 and Fragment F6–7

The length of the F4 fragment subjected to analysis was 763 nt. Based on the structural models generated by the RNAstructure program, the one with the lowest energy of ΔG = −228.7 was selected (Figure S1C). Within the fragment, one quite extensive, new hairpin-type structural element, SL2664–2719, was identified and two shorter elements: SL2313-2343 and SL2562-2590. The long SL2664–2719 hairpin structure has one single-nucleotide bulge and two multi-nucleotide bulges in its body and a six-nucleotide apical loop rich in adenosine and a closing A-U pair (Figure 2 and Figure S1C). Based on the analysis in RNAstructure, the occurrence of the loop is predicted with a high probability of at least 99%, whilst probability of the whole motif was estimated to be over 95%, which corresponds well with the experimental data from the SHAPE experiment (Figure 2 and Figure S1C). The RNA elements SL2313-2343 and SL2562-2590 were predicted with high probability in a region of the F4 RNA, in which structure probing results were not readable enough to be implemented into the RNAstructure program. They are 31 and 29 nt long, both possess one (SL2562-2590) or two (SL2313-2343) internal mismatches in the stem. Both elements have apical loops rich in adenosines, but the loop found in SL2313-2343 is larger (11 nt) than that of SL2562-2590 (5 nt).

For the F6–7 fragment of the CVB3 virus genome, a secondary structural model was chosen that had the lowest energy level of ΔG = −281.7 (Figure S1D). The length of the analyzed fragment was 777 nt. Analysis of this fragment F6–7 successfully identified the previously described CRE (Figure S1D) and also at least two new structural motifs: SL4558-4656 and SL4765-4802. (Figure 2 and Figure S1D). In both cases, the motifs were characterized by a quite high correspondence to the experimental results with the shown structure. The first one, SL4558-4656 was a long, 101 nt stem-loop structure with bulges and internal loops in the stem, and with a 6-nucleotide apical loop rich in pyrimidines (U and C), which could potentially interact with proteins recognizing polypyrimidine stretches, e.g., PTB (Polypyrimidine Tract Binding Protein). The second motif, the 38-nucleotide-long hairpin SL4765-4802, seemed to be very interesting due to the high probability of the formation of the proposed structures, which is similar to the well-characterized CRE, and an 8-nucleotide apical loop rich in adenosine residues. (Figure S1D). A pair of adenosine residues located in a loop of CRE serves as a template for uridylylation of the Vpg-replication primer [14]. This raises the question of whether SL4761-4798 could act as an auxiliary/helper element in the uridylylation of the Vpg protein, since it also harbored two adenosines in the apical loop and was located in a short distance from CRE (Figure S1D). It would be interesting to check what effect the removal of this motif would have on the virus genome on the viral replication process.

#### 3.1.3. Secondary Structure of Partly Overlapping Fragments: F8–9 and F9

The proposal with the lowest free energy of ΔG = −266.7 was chosen as the secondary structure model for the 756 nt long F8–9 fragment (Figure S1E). Three structural motifs are present within the model (Figure 2, SM1E). The SL6236-6260 and SL6376–6399 motifs adopt stem-loop structures. The SL6236–6260 motif is a 25-nucleotide simple hairpin structure, with a large apical loop, with a high (at least 99%) probability of occurrence, but no clear increase of the reactivity for individual loop nucleotides was observed experimentally. It is possible that they are involved in long-range RNA–RNA interactions. SL6376-6399 is a 24 nucleotide structure with one-nucleotide bulge in the stem and an apical loop rich in adenosines. For this motif, the experimental reactivity values fit well with the proposed secondary structure. Both motifs, together with a short, 20-nt stem-loop element SL6289-6308 are shared with F9 RNA (Figure S1E,F).

The structure of the 288 nt F9 molecule with the lowest energy, ΔG = −120.4, contains five interesting motifs, representing a highly probable RNA secondary structure (SL6236-6260, SL6289-6308, SL6376-6399, SL6476-6495, and SL6689-6722) and one less probable one, SL6581-6597 (Figure S1F). Closest to the 5′ end of the F9 molecule, a regular hairpin structure was proposed, called SL6236-6260 (Figure 2). There are nucleotides with a high degree of reactivity in both the loop and stem. Another motif is the SL6378-6395 hairpin, common with the F8–9 fragment (Figure 2 and Figure S1E,F). A third hairpin structure, SL6581-6597, has a lower probability of occurrence in this fragment than the other structures, but it is highly probable in F9–10 (Figure 2 and Figure S1F,G).

#### 3.1.4. Secondary Structure of Partly Overlapping Fragments: F9–10 and F10

For the F9–10 molecule, a 587 nt fragment was experimentally mapped. This fragment has 127 nucleotides shared with F9 and contains the overlapping F10 region, examined with the 9–10R primer. RNAstructure predicted the structure model F9–10, with a free energy of ΔG = −64.0, which contains four highly probable motifs. The region SL6581–6597 forms a short, simple hairpin also observed within the overlapping fragment F9 (Figure 2 and Figure S1F,G). It is predicted with higher probability in the F9–10 RNA. Another motif is the hairpin SL6689-6722 with an additional two internal loops in the stem (shared with F9, Figure S1F). Two further structural motifs, SL6940-6957 and SL6810-6826, have less complex structures, each of which consists of a simple hairpin with or without a mismatch inside the stem, respectively. Each of the three latter elements is present also in the F10 RNA (Figure 3 and Figure S1H).

The length of the fragment of the F10 molecule analyzed was 736 nt, and the structural model proposed by RNAstructure had an energy of ΔG = −286.4. In this approach a free energy value was calculated for a sequence fragment without the terminal pseudoknot structure (Figure 3). We used several different methods to characterize the structure of F10 fragment, proven to be effective in RNA structure mapping. Experimental results of F10 secondary structure mapping with SHAPE (NMIA reactivities) were used as constraints in the process of generating a set of optional structure models by the RNAstructure program, which lie within 10% of the minimal energy. Subsequently, the experimental data from chemical modification with DMS and Pb^2+^ ions were essential while choosing one of several alternative structural models generated by the computer program. In general, results from structural experiments, such as chemical modifications with DMS and RNA cleavage with lead ions, corresponded well to one of the models proposed by computer program RNAstructure and are displayed on the chosen structure (Figure 3). In the F10 model a set of several interesting structural motifs was identified (Figure 2 and Figure 3). Analyzing the calculated probability of the pairing of particular nucleotides, summed up in Figure S1H, we were able to establish which motifs are structurally well defined and occur in a similar form in alternate models of the F10 RNA fragment. We identified a few highly probable motifs of stem-loop/hairpin type in F10: Among the most structurally ordered motifs were: SL6689-6722, SL6738-6761, SL6810-6826, SL6854-6878, and SL6940-6957. Some motifs were found to be less structurally ordered, such as: SL6891-6922, SL6987-7036, SL7159-7194, and SL7247-7291. The SL6689-6722 motif was found and experimentally confirmed in F9 and F9–10, whereas SL6810-6826 and SL6940-6957 were also found within F9–10 RNA.

We identified a structure similar to E-10 hairpin/WT-β element and named it E-10-like hairpin (SL7159-7194; Figure 2 and Figure 3 and Figure S1H). This structure was slightly modified in a few nucleotides present in its stem, in a way that seemed to shift the pairing by one nucleotide, and the element possessed a smaller loop than those proposed for the E-10 structure. It was, however, different from WT-β and its functional role remained to be elucidated.

### 3.2. Phylogenetic Analysis of Selected Structural Motifs Conserved among CVB3 Strains and Other Enteroviruses

**Figure 4:**
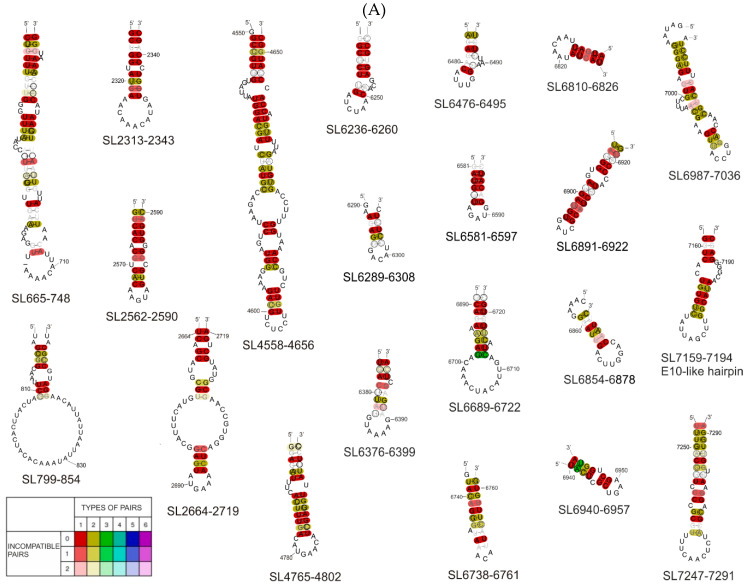

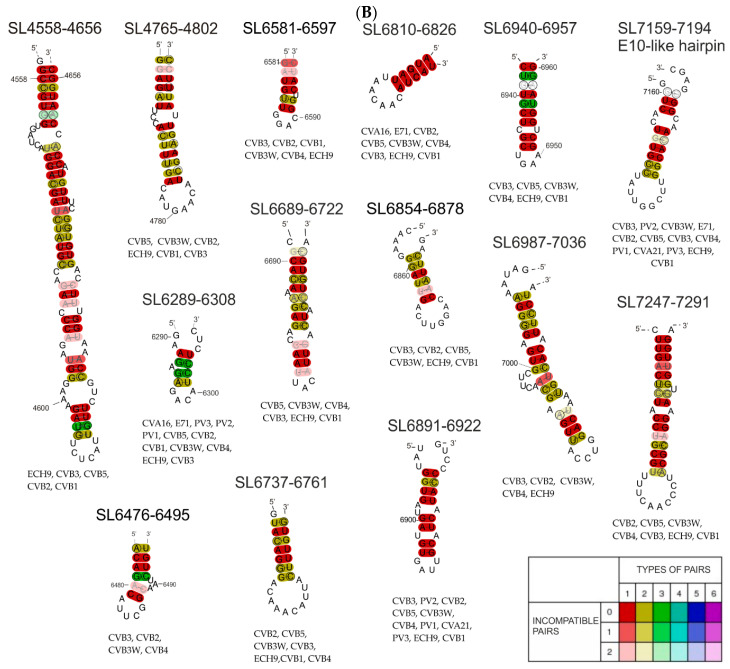
(**A**) Consensus secondary structures generated by the RNAalifold program for particular RNA motifs identified in the coding part of the Coxsackie B3 virus genome, the “Nancy” strain and 19 other CVB3 strains/isolates. The colors indicate structural conservation according to the key in the figure. (**B**) Consensus secondary structures generated by RNAalifold program for particular RNA motifs identified in the coding part of the Coxsackie B3 virus genome, the “Nancy” strain and some of 11 other enterovirus strains we investigated. (CVA16-U05876.1|CAU05876 coxsackievirus A16 G-10; CVA21-D00538.1|CXA21CG Human coxsackievirus A21 (strain Coe); CVB1-M16560.1|CXA1G Coxsackievirus B1; CVB2-AF085363.1|Coxsackievirus B2 strain Ohio-1; CVB3-JX312064.1|Human coxsackievirus B3 strain Nancy; CVB3W-U57056.1|CXU57056 Coxsackievirus B3 Woodruff variant; CVB4-X05690.1|Coxsackievirus B4; CVB5-X67706.1|Coxsackievirus B5; PV1-K01392.1|POL3L37 Poliovirus P3/Leon/37 (type 3; PV2-X00595.1|Poliovirus type 2 genome (strain Sabin 2); PV3-V01150.1|Human poliovirus strain Sabin 1; ECH9-X84981.1|Echovirus 9 (strain Hill); E71-AF176044.1|Enterovirus 71.) The colors indicate structural conservation according to the key in the figure.

Structural motifs found in the analyzed RNA fragments were subjected to phylogenetic analysis in order to establish the degree of their conservation among the other 20 CVB3 virus strains/isolates (Figure 4A and Figure S2), and among other enteroviruses representatives (Figure 4B and Figure S2). The first step in this process was to test if the motifs of interest were specific only to our analyzed virus isolate, or could also be found in a wider range of viruses of the same subtype. For this purpose the Clustal Omega tool was used. Later, results from Clustal Omega were analyzed with RNAalifold, which generated consensus secondary structures.

When combining the SL665-765 motif sequence with the 20 CVB3 isolate sequences, the RNAalifold proposed a slightly different structure than the model proposed in the RNAstructure program (Figure 4A). The difference mainly concerned the sequence of the first and last nucleotides making up the core stem of the entire motif. When the motif sequence was combined with the sequences of representatives of a broader phylogenetic group (11 other enteroviruses), the RNAalifold program was not able to propose a probable common model, which suggests that this hairpin/motif is not shared by all enteroviruses. It is most likely to form in the group of 19 CVB3 isolates closely related to the Nancy strain and could have functional significance only in a very narrow phylogenetic group. In the case of SL799-854, we observed a similar situation.

After the comparison of the SL799-854 motif sequence with the 20 sequences from isolates of CVB3, the RNAalifold program proposed a structural model that differed very slightly from that proposed by the RNAstructure program (Figure 4A). Phylogenetic analysis confirmed that the SL799-854 motif may also occur in other isolates of the CVB3 virus (Figure 4A and Figure S2), but it is not shared with other enteroviruses. A broader analysis in combination with the sequences of 11 enterovirus representatives shows that enough of the motif is present to form the same loop but the stable stem is most likely not possible (Figure 4B).

In both cases of phylogenetic analysis of SL2664-2719 from the F4 fragment, the program proposed a slightly different secondary structure than that found by RNAstructure (Figure 4). In the case of the structure model proposed by RNAalifold, there was one large bulge consisting of 19 nucleotides and another smaller one, consisting of 7 nucleotides and a 6 nucleotide apical loop, while for the same region the RNAstructure program proposed a structure consisting of three bulges ending with the same apical loop. It is also characterized by a large number of compatible nucleotide pairs formed within the motif core. With regard to the sequence analysis of five selected enteroviruses (Coxsackie B1, B2, B4, B5 virus, and echovirus), the sequence also shows the same general structure, differing in the presence of only one small inner loop instead of two, and one bulge proposed by RNAstructure. (Figure 4B). Despite the slight differences in motif formation, the structure of SL2664–2719 shows high evolutionary conservation within CVB3 isolates (Figure 4A). However, only selected representatives of enteroviruses, Coxsackie B1, B2, B4, B5, and echovirus, had the ability to form the proposed structure (Figure 4B).

Phylogenetic analysis using the RNAalifold program of 20 CVB3 isolates showed that the SL4551-4651 motif is quite well conserved, with only slight differences regarding the position of the paired nucleotides in the stem (Figure 4A). Comparing the sequence of this motif within enteroviruses, it was noted that despite slight differences in terms of the location of the nucleotides, and the loops and bulges they form, in these enteroviruses, the pyrimidine-rich loop is preceded by a long double-stranded stem (Figure 4B).

Motif SL4765-4802 in the phylogenetic analysis was found to be conserved within CVB3 isolates and selected enterovirus representatives (echovirus, Coxsackie virus strains B1, B2, B3, and B5; Figure 4B). The same nucleotides interacted in the same way to form the entire structure identical to that proposed by RNAstructure, which indicates a high probability of the proposed motif.

Within the F8–9 fragment, two new motifs were identified: SL6236-6260 and SL6376-6399, however, phylogenetic analysis did not confirm the universal nature of these motifs among enteroviruses. The fact that they are not present in other enteroviruses, and, in addition, they are lacking in many CVB3 isolates (Figure S2), suggesting that the motifs are not of universal significance, although further analysis may find functional significance for the Coxsackie B3 virus of the strain Nancy. In contrast, the phylogenetic analysis carried out for 20 CVB3 isolates shows a high probability, indicating a great degree of conservation, of the SL6236-6260 motif (Figure 4A). Among the analyzed isolates, for the SL6376-6399 motif, the RNAalifold program generated a structure corresponding to that proposed by RNAstructure, but the high variability within the stem interferes with nucleotide base-pairing and makes the probability of this motif, even for CVB3 isolates, very low. Based on the phylogenetic analysis of the sequence of both motifs within selected enteroviruses, no common structure was obtained for them that would correspond to that obtained in the RNAstructure program. This indicates a low level of conservation of these motifs within the enteroviruses (SL6376-6399).

In the case of motifs SL6289-6308, SL6476-6495, and SL6689-6722, the structural model differed slightly from that obtained in the RNAstructure program, this particularly concerned the number of base pairs in double-stranded stems and the number of nucleotides in the loops. These results suggest a high probability of occurrence of RNA motifs proposed in this work in all 20 CVB3 virus isolates analyzed.

Phylogenetic analysis of proposed secondary structural motifs found in F10 RNA among different CVB3 isolates and later for different representatives of enteroviruses was performed. In the first comparison of CVB3 genomes, ten motifs were found to be strongly conserved and capable of creating similar structures, despite the variability of the sequence (Figure 4A). Some of them can also be found in other enteroviruses: SL6689-, SL6737-, SL6810-, SL6854-, SL6891-, SL6940-, SL6987-, SL7247-, and the E-10-like hairpin (Figure 4B). This observation increased the probability of these motifs being important factors in the viral life cycle. One of such motif is the previously mentioned E-10-like hairpin. Despite the fact that this element did not seem to be easily predicted by computer programs within equivalent models of this F10 RNA fragment, it is strongly conserved among enteroviruses (Figure 4). Particularly noteworthy is the structure SL6376-6399 containing a stretch of four adenosines in the apical loop, which can be a motif recognized by RNA binding proteins found in host cells, for example PABP.

Alignments of all analyses, together with virus strain names, are presented in Figure S2.

### 3.3. RNase L Assay-Limited Digestion of 3′-Terminal Fragment (F10) of CVB3 Genome with Ribonuclease L

We carried out another analysis of CVB3 RNA with RNase L digestion assays. Following RNA renaturation, samples were incubated with two different concentrations of RNase L and a sample without this reagent was used for control purposes. Sites of digestion were detected by reverse transcriptase in primer-extension reactions and analyzed electrophoretically. Observed RNase L digestion sites matched single-stranded regions of the structural model of F10 RNA as well, in accordance with specific activity of the enzyme (U/UN) [49].

Early research based on the prediction of RNA structure in silico and bioinformatic analysis found that, apart from the well-characterized non-translated regions, the genome of enteroviruses does not appear to contain conserved structural motifs [12], aside from the CRE identified in the viral protease coding region and one other small element of unknown function, the E-10 hairpin. However, it turns out that these conclusions were drawn based on the analysis of too wide a phylogenetic group. When looking at the narrower subgroup of C enteroviruses, an extremely interesting RNA structure has been found in their coding part, which is responsible for direct RNase L inhibition, a key factor in triggering the cell’s antiviral response [16,17,18,49].

From studies on the hepatitis C virus, which, like Coxsackievirus B, is susceptible to digestion with RNase L, it has been reported that a dozen or so RNA structural motifs are excised from the coding part of its genome during infection, and that at least some of them can serve the host by signaling the presence of an infection through the antivirus defense signal cascade [20]. In turn, somewhat in contrast to these observations, numerous structural motifs present in the genomes of RNA viruses can prevent the recognition of infection at the cellular level and thereby facilitate the spread of the virus [50].

We decided to characterize which fragments of the CVB3 genome are cut out by human ribonuclease L from its terminal region. Observed RNase L digestion sites within the F10 RNA matched single-stranded regions of the structural model well, in accordance with specific activity of the enzyme, providing additional evidence of the accuracy of the model (Figure 3). Some of structural motifs, such as SL6940-6957, SL7159-7194, and SL6891-6922, seem to be cut out of the RNA fragments by RNase L (Figure 3, SM3). They might play an important role in the cellular response to virus infection, as it shown in HCV studies, by acting, for example, as superinductors of the interferon response.

### 3.4. Investigation of the Dimerization Process of F10 Fragment Representing 3′-Terminal Region of the CVB3 Genome

**Figure 5:**
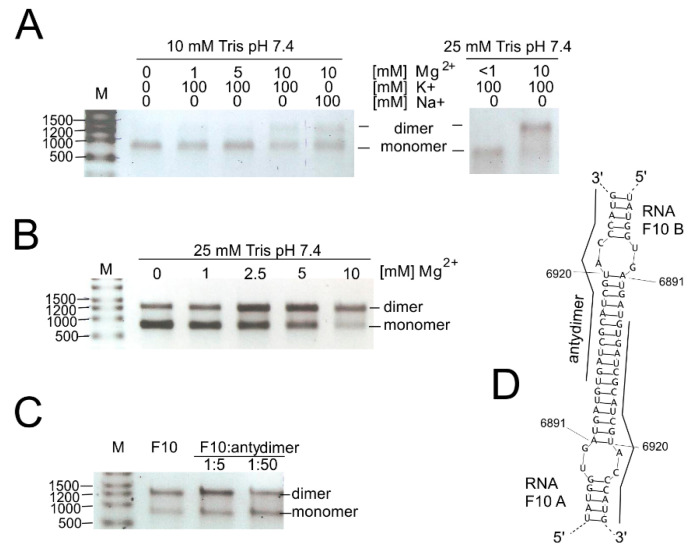
Dimerization of the F10 RNA, the 3′-terminal region of the CVB3 genome. (**A**) Impact of ionic and buffer concentrations; (**B**) impact of divalent magnesium ions on dimerization process; (**C**) inhibition of homodimer formation in the presence of “antydimer” (DNA 18-mer), complementary to the F10 in the region of the potential dimerization site; and (**D**) computer predictions of the region responsible for F10-homodimer formation.

We also investigated the possibility of genome dimerization and RNase L digestion in the 3′-terminal region of the CVB3 genome. Both processes seem to involve the RNA element SL6981-6920. During electrophoresis of RNA products in the RNase L assay, we found that the F10 RNA fragment migrates in the form of two major bands. Moreover, the shorter band was of the expected length of the full-length F10 fragment (ca. 750 nt) and thus could not represent a digestion or degradation product. The upper band migrated close to 1500 nb of the RNA marker (and 1.8S rRNA), suggesting that it represents a double-length F10 fragment (Figure 5). This suggested that it might be a homodimer made up of F10 RNA molecules. Dimerization of viral RNA genomes has been observed in retroviruses, i.e., HIV [51], and proposed for HCV (reviewed in [11]), and may be important in the viral life cycles of CVB3 and other enteroviruses as well.

First, we checked if renaturation conditions, like time, temperature, and ion concentration, influence the dimerization process (Figure 5A–C). It turned out that the stronger Tris buffer, 25 mM, versus 10 mM, promoted dimerization of the F10 fragments. Both Na^+^ and K^+^ ions gave the same results, so we could exclude G-quadruplex formation, which is dependent on the presence of potassium ions [52]. The duplex formation is, however, sensitive to the concentration of Mg^2+^ ions (Figure 5B). Homodimer formation was slightly inhibited in the presence of a short ssDNA oligomer complementary to the dimerization site (SM3B). Computer predictions of F10-dimer formation indicated a region capable of forming a double-stranded stem between two identical F10 molecules (F10 A and F10 B; bimolecular simulation in RNAstructure program; Figure 5D). It is worth mentioning that this region encompassed a hairpin motif (SL6981-6922) formed by single-molecules of RNA and that this motif was excised by RNase L (Figure 2 and Figure 3, and Figure S3).

### 3.5. High Throughput Protein Analysis Reveals Proteins, which are able to Bind to the 3′-Terminal Part of the CVB3 Genomic RNA

We were curious to know whether protein binding sites exist within the examined fragments of the CVB3 genome. Based on data collected in the ATtRACT database (A database of RNA binding proteins and associated motifs, https://attract.cnic.es/index) we did a sequence search for each newly identified RNA motif to find potential protein binding sites (Table 2 and Table S4) [40]. We found many potential protein binding sites for RNA-or DNA/RNA binding proteins, such as heterogeneous nuclear ribonucleoproteins, poly-track-binding proteins (poly-A, poly-C, poly-Y, etc.), splicing factors, RNA stabilizing and destabilizing proteins, transcription regulators; translation regulators, proteins that are known to be involved in host–virus interactions, and some other interesting factors. For some proteins, multiple binding sites were observed within a single motif sequence (Table 2 and Table S4).

In order to search for proteins that are able to bind to the F10 RNA-3ʹ terminus of CVB3 genome, we applied cell extracts and RNA-centric affinity chromatography, combined with mass spectrometry analysis [41,42,43]. As RNA bait we used an RNA oligomer F10, (see the Materials and Methods section: in vitro transcription), which partially corresponded to the RdRP coding region and the 3′UTR of the CVB3 genome. Cytoplasmic fractions were prepared from untreated human MCF-7 cells and following RNA-affinity chromatography, the proteins were identified by MS/MS analysis [43]. To identify and eliminate proteins that were bound non-specifically, we used samples eluted from agarose beads, which were not covered by RNA bait as controls (Figure S5). The Table S5A shows proteins with the number of MS spectra hits ranging from 1 to 67, after correction with the control samples. Approximately 24% of the proteins were classified as ribosomal proteins. The most abundant group, around 36% of the total identified proteins, were considered as part of the proteins that bind RNA with low affinity. In this group, we identified proteins with a high potential for binding to nucleic acids, RNA or both RNA and DNA, such as cytoskeletal tubulins and myosins, translation-, and transcription factors, and histones-10% of the total identified proteins (Table S5B). More than 30% of the total identified proteins were considered as the candidate group, whose affinity to RNA was at least plus (more than) 5 MS spectra hits bigger than the probe with naked beads, without RNA.

**Figure 6:**
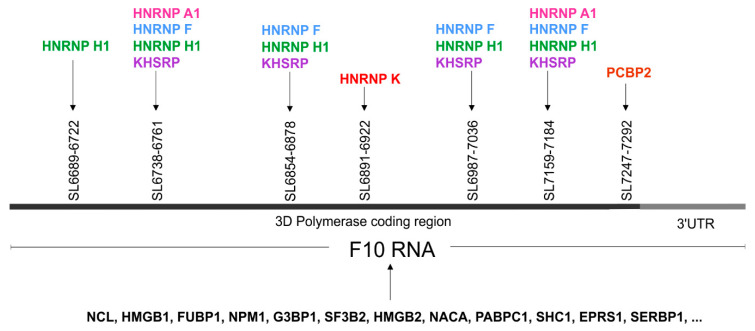
The proteins identified in the RNA-centric affinity chromatography that were found to be interacting with F10 RNA fragment from the 3′-terminus of the CVB3 genome. Possible localizations of the RNA–protein interactions for several proteins is given, based on the presence of a predicted sequence binding site (ATtRACT database) within the F10 sequence. The figure shows the gene names rather than full protein names to minimize the space required. Different colors represent distinct proteins.

The most frequent protein our analyses discovered was nucleolin. We also found high mobility group proteins B1 and B2, Far upstream element-binding proteins 1 and 2, heterogeneous nuclear ribonucleoproteins (K, A1, F, and H1), polyadenylate binding protein 1, and poly(rC) binding protein 2, to name but a few. Several proteins hnRNP K, A1, F, H1; KHSH, and PCBP1, which were found to bind to the 3′-terminal of the CVB3 genome, were also predicted by ATtRACT, and may be involved in viral replication processes (Figure 6). Interestingly, heterogeneous nuclear ribonucleoprotein K (hnRNP K) was one of the highest scoring candidates and is known to be a member of the replication complex of human cytomegalovirus HCMV [53]. Heterogeneous nuclear ribonucleoprotein A1, known also as a helix-destabilizing protein, often inhibits IRES-directed translation that could be important during the switch from translation to replication in the virus life cycle. HNRNP A1 may play a role in HCV RNA replication, and can be digested by enterovirus 71 protease 3C, leading to apoptosis [54,55]. KHSRP binds and destabilizes mRNA [56]. PCBP2 is a single-stranded nucleic acid binding protein that binds preferentially to tracks of oligo(C) and poly(rU) and negatively regulates cellular antiviral responses mediated by MAVS signaling [57]. Another candidate, PABPC1 positively regulates the replication of dengue virus [58]. It is also involved in shutoff of host mRNA translational machinery for viral needs by inhibiting cellular PABPC1 activity using different mechanisms. Picornaviruses encode a protease that cleaves PABPC1 at several defined sites in the proline-rich linker region between RRMs and the C-terminal domain. Rotaviruses, gamma herpesviruses, and bunyamwera virus relocalize PABPC1 from the cytoplasm to the nucleus and thus alter its function. Many of these viruses translate their mRNA in a PABPC1-independent manner and are unaffected by host PABPC1 inhibition.

Taken together, the RNA-centric affinity chromatography approach combined with MS analysis revealed several proteins, which were able to bind to the newly identified RNA structure motifs within the 3ʹ-terminal region of CVB3 RNA, that potentially might have functional implications for the CVB3 propagation (Figure 6 and Figure S5A–C).

## 4. Concluding Remarks

In this study we used both experimental and bioinformatic methods to study the secondary RNA structure and comparative phylogenetic studies to find new stable and conserved structural elements in the CVB3 genome, mainly in its protein coding part. Among twenty one of the RNA secondary structure motifs, we identified many with a potentially significant role for the propagation of the virus due to their high probability of occurring in the genomes of twenty different CVB3 virus isolates. Some of these RNA elements were found in other enteroviruses in similar forms as well. The RNA-centric affinity chromatography approach, combined with MS analysis, revealed several proteins that were able to bind to the newly identified RNA structure motifs within the 3ʹ-terminal region of CVB3 RNA, which potentially have functional implications for the propagation of CVB3. We also investigated the possibility of genome dimerization and RNase L digestion in the fragment corresponding to the 3′-terminal region of the CVB3 genome. Both processes can be important in the viral life cycle of CVB3 and other enteroviruses or in the antiviral response of the infected organism. The results presented in this study contribute to a better understanding of relationships between the structure and function of RNA elements found in the coding region of CVB3 and will serve as a starting point for functional studies of their importance in viral life cycle and host persistence.

## Figures and Tables

**Table 1:** Primers used in this study. F-used as forward primers; R-used as reverse primers, also 5′-fluorescently labeled with VIC, FAM, PET, or NED where indicated.

No.	Primer Name	Nucleotide Sequence
1	1F	5′-TAATACGACTCACTATAGGAAACAGCCTGTGGGTTGATCC-3′
2	1R	5′- CTAGCATTCAGCCTGGTCTC-3′
3	1–2F	5′-TAATACGACTCACTATAGGTCTAATACAGACATGGTGCGAAG-3′
4	1–2R	5′-TCTGCCATTGCACAGAGTCAAG-3′
5	4F	5′-TAATACGACTCACTATAGGTGTTGCTTCAGATGAGTATACCG-3′
6	4R	5′-AAATTCAGACCATCCGTCATAG-3′
7	6–7F	5′-TAATACGACTCACTATAGGCGAGTTCCTGAACAGACTTAAAC-3′
8	6–7R	5′-TAGAGAGTATCTGACCTGTGTTC-3′
10	8–9F	5′-TAATACGACTCACTATAGGCAATTAACACCAGCAAGTTTCC-3′
11	8–9R	5′-TTCCTTTCGCTACCTTCTCTATG-3′
12	9F	5′-TAATACGACTCACTATAGGGTTTCCAGTCATCAACACACC-3′
13	9R	5′-GGAGTTGCACAAGTAGTCAATG-3′
14	9–10F	5′-TAATACGACTCACTATAGGGAACCTACCAATGGTGACTTATG-3′
15	9–10R	5′-GATTCGTGTATGTCTTTCATGG-3′
16	10F	5′-TAATACGACTCACTATAGGCTTGGATACACGCACAAAGAG-3′
17	10R	5′-GCACCGAATGCGGAGAATTTAC-3′
18	10+(R)	5′-TTTTTTTTTTTCCGCACCGAATGC-3′

**Table 2:** Features of the new RNA elements/motifs found in the CVB3 genome. The structure motifs were experimentally characterized with the indicated method/s, (SHAPE) or (SHAPE, Pb^2+^, DMS)—(Figure 2 and Figure 3, and Figure S1); (−)—experimentally uncharacterized; (H)—structure predicted by RNAstructure with high probability; (L)—structure predicted with low or moderate probability (Figure 2 and Figure 3, and Figure S1); (CVB3)—motif specific for different CVB3 strains according to RNAalifold; (E)—motif also conserved in other enteroviruses (Figure 4A (CVB3), Figure 4B(E), and Figure S2); potential interactions with proteins were proposed with help of the ATtRACT database [40]; their gene names are indicated in this table (Table S4). * Interactions with proteins and F10 RNA, which were experimentally confirmed in RNA-centric affinity chromatography (Figure S5); nt-length of a motif/number of nucleotides.

RNA Structure Motif	Length (nt)	Genome Region	RNA Fragment in which the Motif was Experimentally Characterized	RNA Fragment in which the Motif was Thermodynamically Predicted	Motif Structure Conservation	Potential Interactions with Proteins According to ATtRACT Database and RNA-Centric Affinity Chromatography *
SL665-748 SL652-752 (with bulge)	84 101	5′UTR /V4	F1, F1–2 (SHAPE)	F1 (H), F1–2 (L)	(some CVB3)	CELF1, CELF 2, ELAVL2, ENOX1, GRSF1, HNRNP (A1, A2B1, F, H1, H2, H3, K, L), IGF2BP2, IGF2BP3, KHDRBS1, KHSRP, NOVA1, NOVA 2, OAS1, PCBP2, PIWIL1, PPIE, PTBP1, RBMS3, RBMX, RNASEL, SRP (19, 54, 68), SRSF (2, 5, 9, 10), TIA1, TIAL1, XPO5, YTHDC1, ZFP36, ZRANB2
SL799-854	54	V4	-	F1–2 (H)	(CVB3)	A1CF, HNRNPL, IGF2BP3, MBNL1, NOVA1, NOVA2, PABPC1, PPIE, PTBP1, RBMY1A1, SRSF3, SRSF9, TIA1, TIAL1, XPO5, YBX1, YTHDC1,
SL2313-2343	31	V1	-	F4 (H)	(some CVB3)	HNRNPL, HNRNPLL, IGF2BP2, IGF2BP3, NOVA1, PABPC1, PTBP1, RBMX, SAMD4A, SRP14, ZNF346
SL2562-2590	29	V1	-	F4 (H)	(some CVB3)	FUS, HNRNPL, KHSRP, MBNL1, NOVA1, NOVA2, RBMY1A1, SRSF (1, 2, 5, 6), YTHDC1, ZRANB2, RBMX,
SL2664-2719	55	V1	F4 (SHAPE)	F4 (H)	(CVB3)	CELF2, CPEB4, ELAVL2, FUS, IGF2BP2, IGF2BP3, KHDRBS1, LIN28A, MBNL1, NOVA1, NUDT21, PTBP1, RBFOX1, RNASEL, SRP54, SRP68, SRSF (1, 2, 5, 6, 9), SSB, TIA1, TIAL1, YTHDC1, ZRANB2,
SL4558-4656	99	2C	F6–7 (SHAPE)	F6–7 (H)	(E)	ACO1, CELF1, CELF2, CMTR1, ELAVL (1, 2, 4), ESRP2, FUS, GRSF1, HNRNP (F, H1, H2, H3, K, L), KHSRP, MBNL1, NOVA1, NOVA2, NUDT21, NXF1, PTBP1, RBM28, RC3H1, SRP14, SRSF (1, 2, 3, 6, 9), SSB, TIA1, TIAL1, TRA2B, XPO5, YBX1, ZFP36, ZNF346,
SL4765-4802	38	2C	F6–7 (SHAPE)	F6–7 (H)	(E)	CELF2, ELAVL (1, 2, 4), HNRNPL, NOVA1, NOVA2, OAS1, PHAX, PPIE, PTBP1, RC3H1, SRP54, SRSF (2, 3, 9), SSB, TIA1, TIAL1, YBX1, YBX2, ZRANB2
SL6236-6260	25	3D	F8–9 (SHAPE)	F8–9 (H), F9 (H)	(CVB3)	ADAR, CMTR1, DDX58, DHX9, HNRNPL, MBNL1, OAS1, PIWIL1, PTBP1, QKI, SRSF3, YBX1, YBX2, YTHDC1
SL6289-6308	20	3D	F8–9, F9 (SHAPE)	F8–9 (H), F9 (H)	(CVB3) (E)	F2, GRSF1, HNRNP (F, H1, H2, H3, L), KHSRP, NONO, PABPN1, PTBP1, PTBP2, SRP (19, 54, 68), SRSF (10, 2, 3, 5), TRA2B, YBX1, ZFP36,
SL6376-6399	24	3D	F8–9, F9 (SHAPE)	F8–9 (H), F9 (H)	(CVB3)	CELF2, NUDT21, PABPN1, PTBP1, QKI, RNASEL, SF1, SRP19
SL6476-6495,	20	3D	F9 (SHAPE)	F9 (H)	(CVB3)	ELAVL (1, 2, 4), HNRNPL, IGF2BP3, NXF1, PTBP1, SSB, ZRANB2
SL6581-6597	17	3D	F9–10 (SHAPE)	F9–10 (H), F9 (L)	(CVB3)	CELF1, CELF2, HNRNPA1, HNRNPL, KHSRP, SRSF (1, 2, 3, 9), YBX1,
SL6689-6722	34	3D	F9–10 (SHAPE)	F9–10 (H), F10 (H)	(E)	CELF1, FXR2, HNRNP (H1*, H2, L), IGF2BP3, NXF1, OAS1, PTBP1, RBMY1A1, SRSF10, YTHDC1, ZFP36,
SL6738-6761	24	3D	-	F10 (H)	(E)	CELF1, ESRP1, GRSF1, HNRNP (A1*, F*, H1*, H2, H3, L), IGF2BP3, KHDRBS2, KHDRBS3, KHSRP*, NONO, PTBP1, RBM41, SRSF5, TIA1, QKI, SF1, TIAL1
SL6810-6826	17	3D	F9–10, F10 (SHAPE, Pb^2+^, DMS)	F9–10 (H), F10 (H)	(E)	HNRNPL, NOVA1, NOVA2, QKI, SF1, TIAL1
SL6854-6878	25	3D	F10 (SHAPE, Pb^2+^, DMS)	F10 (H)	(E)	EIF4B, ESRP1, GRSF1, HNRNP (F*, H1*, H2, H3), KHSRP*, NONO, OAS1, PTBP1, SRSF (10, 5, 9), TIA1, TIAL1
SL6891–6922	32	3D	F10 (SHAPE, Pb^2+^, DMS)	F10 (L)	(E)	CELF2, CMTR1, FUS, HNRNPK*, NOVA1, PHAX, RBM46, SRSF (1, 3, 5, 6), YBX1, YTHDC1
SL6940-6957	18	3D	F9–10, F10 (SHAPE, Pb^2+^, DMS)	F9–10 (H), F10 (H)	(E)	F2, MBNL1, PTBP1, SAMD4A, SRSF1
SL6987-7036	50	3D	F10 (SHAPE, Pb^2+^, DMS)	F10 (L)	(E)	CPEB4, ELAVL2, F2, GRSF1, HNRNP (F*, H1*, H2, H3, L), KHSRP*, MBNL1, NONO, NOVA1, NXF1, PIWIL1, PTBP1, RC3H1, SRP68, SRSF (1, 2, 9), TIA1, TIAL1, YTHDC1, ZFP36, RBMX
E-10 like/ SL7159-7194	36	3D	F10 (SHAPE, Pb^2+^, DMS)	F10 (L)	(CVB3) (E)	CELF1, CELF2, GRSF1, HNRNP (A1*, F*, H1*, H2, H3), KHSRP*, NOVA1, NOVA2, OAS1, PIWIL1, PTBP1, SRP (19, 54, 68), SRSF2, SRSF5, TIAL1
SL7247-7291	45	3D	F10 (SHAPE, Pb^2+^, DMS)	F10 (L)	(E)	AGO1, DHX9, ELAVL1, ELAVL2, F2, OAS1, PCBP1, PCBP2*, PTBP1, RC3H1, SFPQ, SRP (14, 19, 54, 68), SRSF (1, 5, 9), SSB, TIA1, TIAL1, TRA2B, XPO5, ZFP36, ZNF346, RBMX

## Supplementary Materials

The following are available online at https://www.mdpi.com/1999-4915/12/11/1232/s1, Figure S1. Secondary structure models of the analyzed RNA fragments. The models display SHAPE reactivities, according to legend. (**A**). Model of the F1 RNA (without domains I–V of IRES); (**B**) model of the F1-2; (**C**) model of the F4 RNA; (**D**) model of the F6-7 RNA; (**E**) model of the F8-9 RNA; F) model of the F9 RNA; (**G**) model of the F9-10 RNA; and (**H**) model of the F10 RNA (without a terminal pseudoknot). Figure S2. Consensus secondary structures and alignments generated by the RNAalifold program for particular RNA motifs identified in the coding part of the Coxsackie B3 virus genome, the "Nancy" strain and 19 other CVB3 isolates, or for “Nancy” and other enterovirus strains. The colors indicate structural conservation according to the key. Figure S3. RNase L assay. (**A**) The viral RNA (F10 fragment of CVB3) or cellular (MCF7) total RNA was subjected to increasing amounts of RNase L: 0.25 nM and 830 nM. Reaction products were electrophoretically separated on agarose gel (**B**–**C**). (**A**) Of the CVB3 genome 750-nt-long 3′-terminal fragment was digested with RNase L at the given concentrations: 0, 125 nM, and 500 nM. Reaction products were separated on a 12% PAA gel (**B**) or in the capillaries (**C**) in parallel to sequencing lines. Blue peaks—RNase L cleavages; green peaks—control reaction. (**D**) RNase L cleavage sites are displayed on a secondary structure model of the viral RNA fragment, the SL6891-6922 motif, as blue arrows. Table S4. Potential interactions with proteins of the newly identified RNA motifs in CVB3 genome fragments. Content based on data collected in the ATtRACT database: (**A**) database of RNA binding proteins and associated motifs, https://attract.cnic.es/index) [40]; mode-sequence search. Figure S5. RNA affinity chromatography and MS analysis. Table S5A. The top candidate protein list. The MS data from the RNA-centric affinity chromatography experiment is included in the table. The table shows the total numbers of MS matches/hits for each candidate protein and the number of hits after correction with the control reaction (red font); S5B. Distribution of proteins identified by F10-CVB3 RNA affinity chromatography and MS analysis for untreated MCF-7 cells; S5C. F10-CVB3 RNA affinity chromatography for untreated MCF-7 cells. Total cytoplasmic fractions and the protein eluates were incubated for 5 min at 95 °C and then loaded on a 10% SDS-PAGE gel. After electrophoresis the gel was silver stained according to the manufacturer’s protocol (Pierce Silver Stain Kit).
