# Supplementary material for: New RNA Structural Elements Identified in the Coding Region of the Coxsackie B3 Virus Genome
Source: Viruses. 2020 Oct 30;12(11):1232. doi: 10.3390/v12111232 (PMC7692623; doi:10.3390/v12111232)
Supplement: Supplementary file 1 [file viruses-12-01232-s001.zip › SM1.pdf]

Figure SM1. Secondary structure models of the analyzed RNA fragments. The models display SHAPE reactvities, according to legend. A). Model of the F1 RNA (without domains I-V of IRES); B) Model of the F1-2 RNA; C) Model of the F4 RNA; D) Model of the F6-7 RNA; E) Model of the F8-9 RNA; F) Model of the F9 RNA; G) Model of the F9-10 RNA; H) Model of the F10 RNA (without terminal pseudoknot).

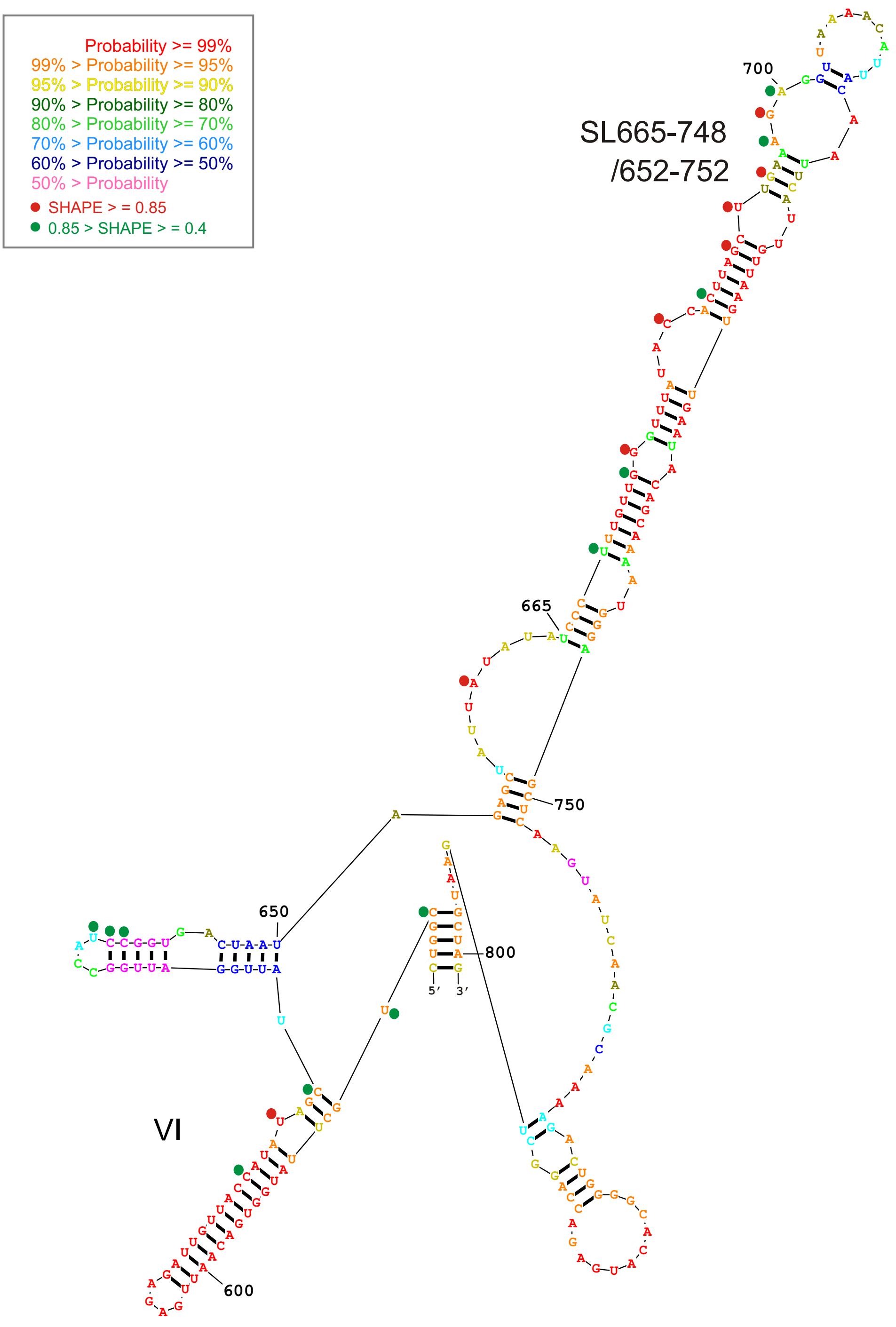

A. The structural model of F1 RNA without cloverleaf and IRES (domains I-V)

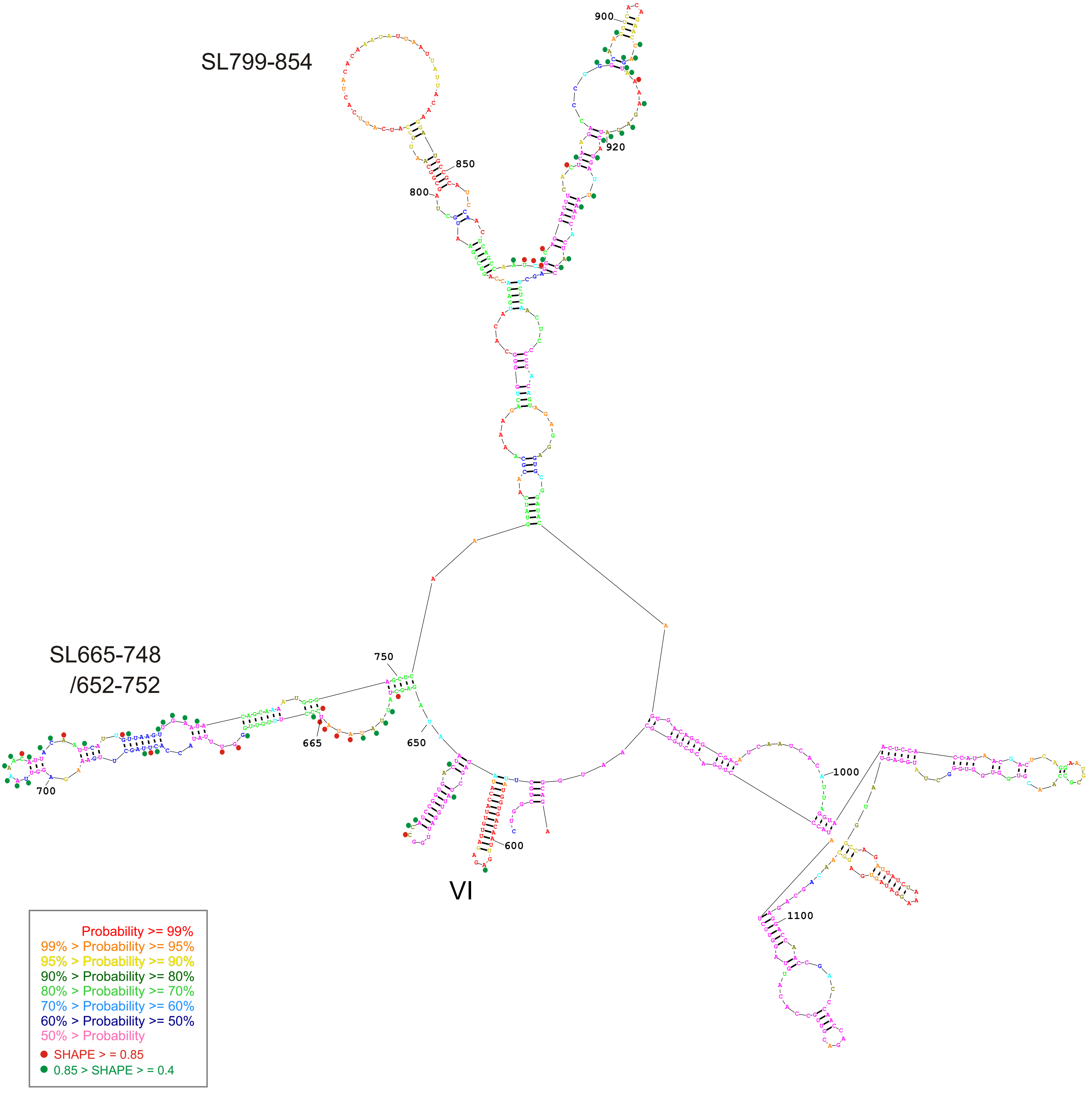

B. The structural model of F1-2 RNA without IRES (domains III-V)

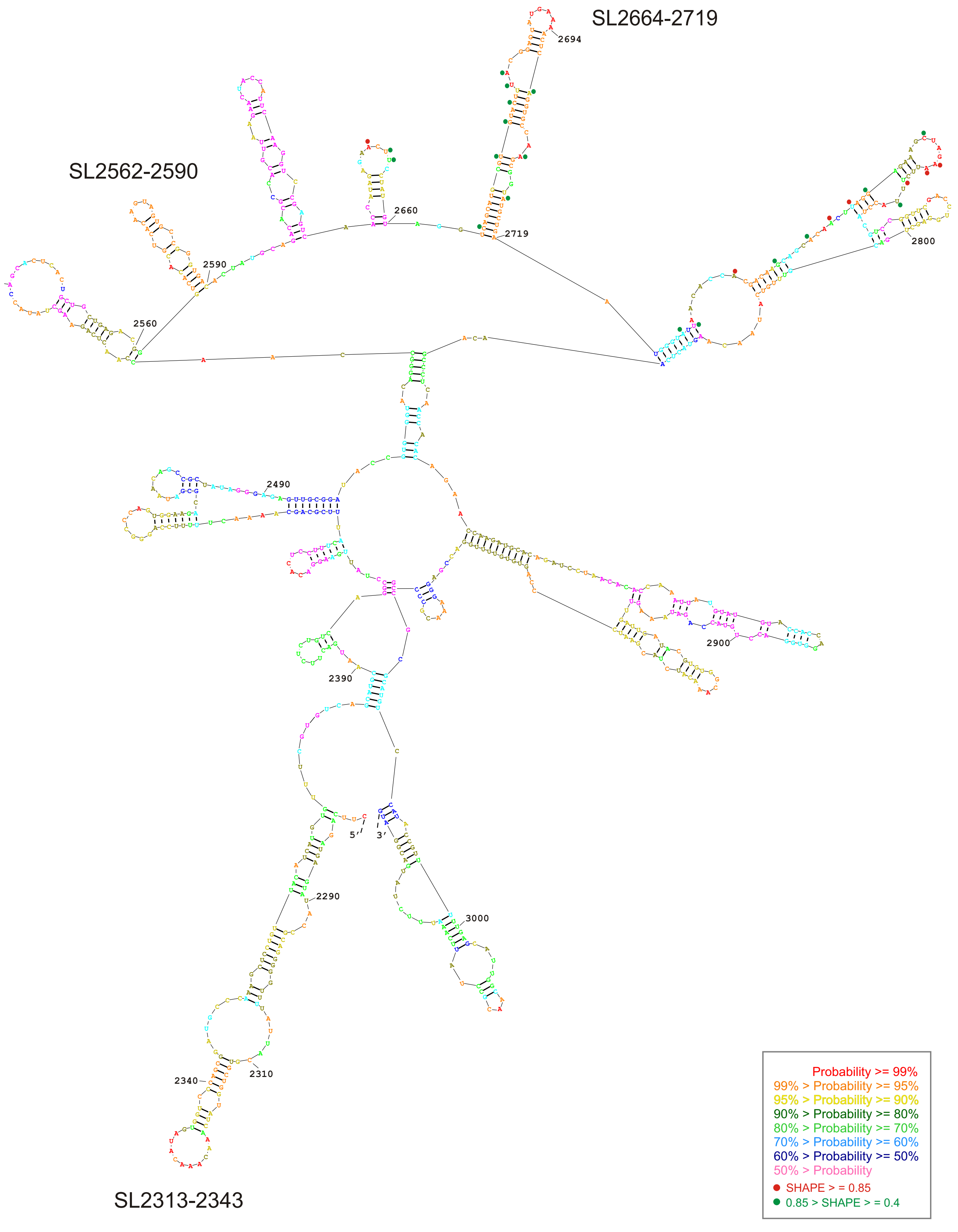

C. The structural model of F4 RNA

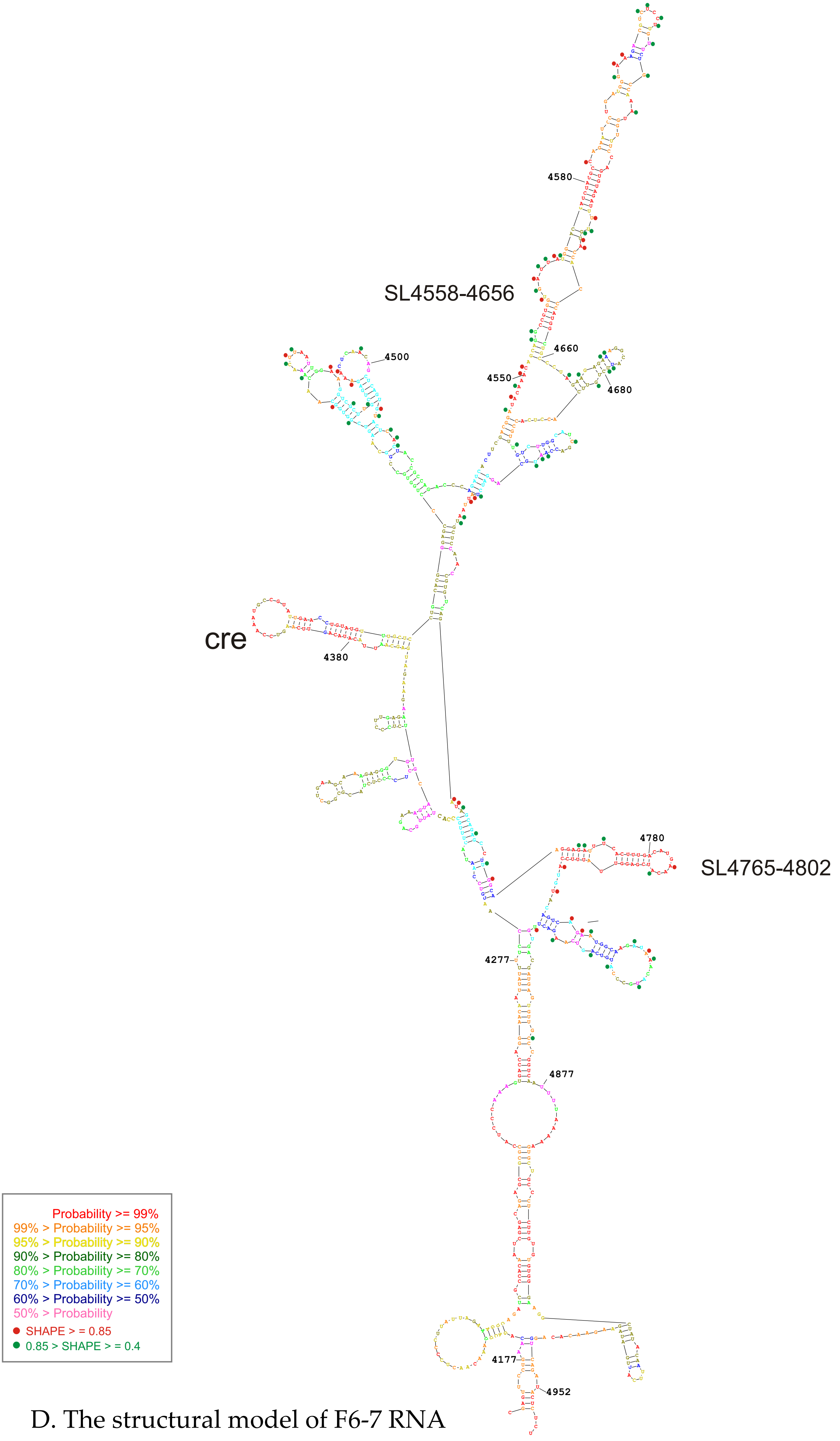

D. The structural model of F6-7 RNA

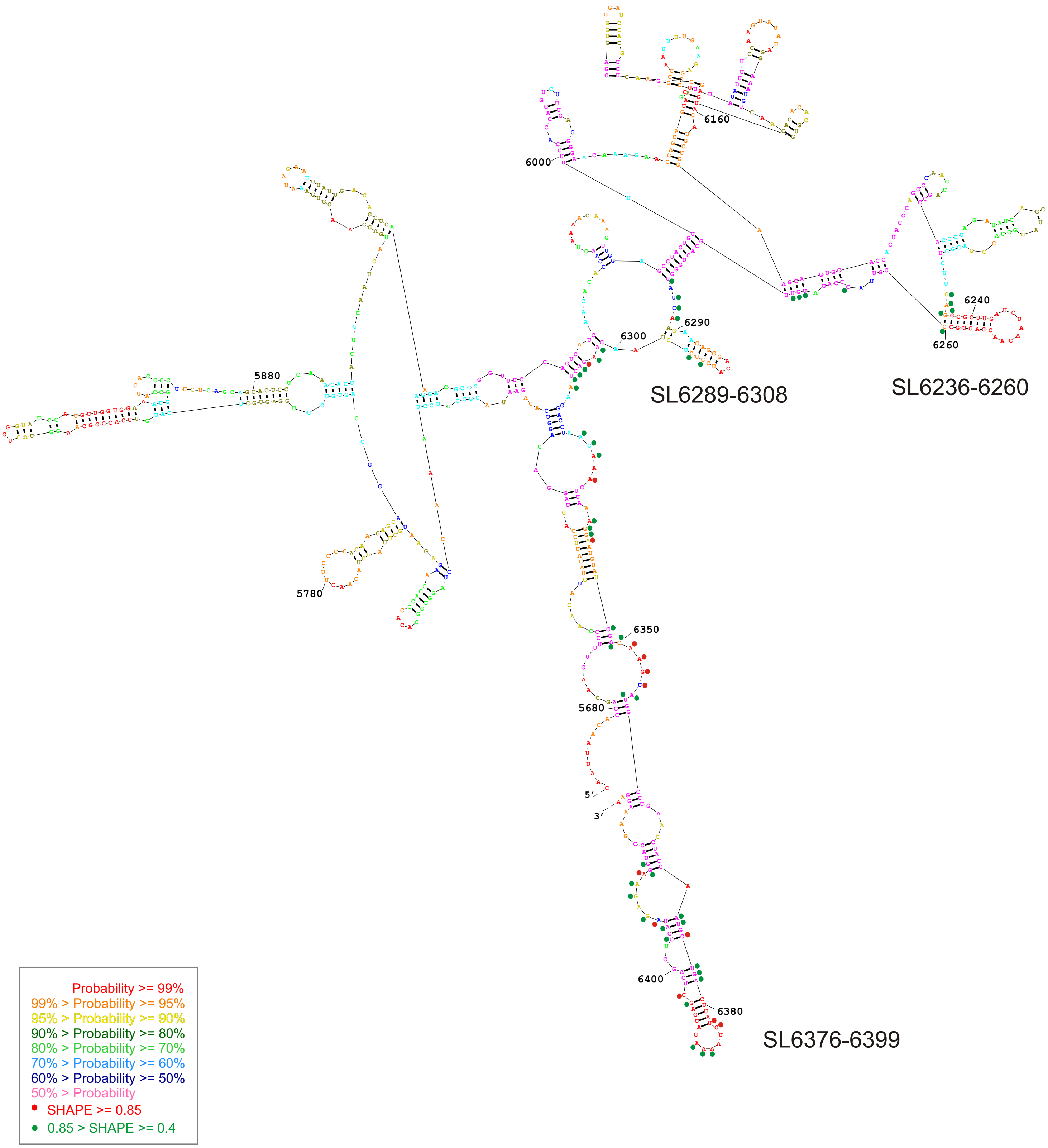

E. The structural model of F8-9 RNA

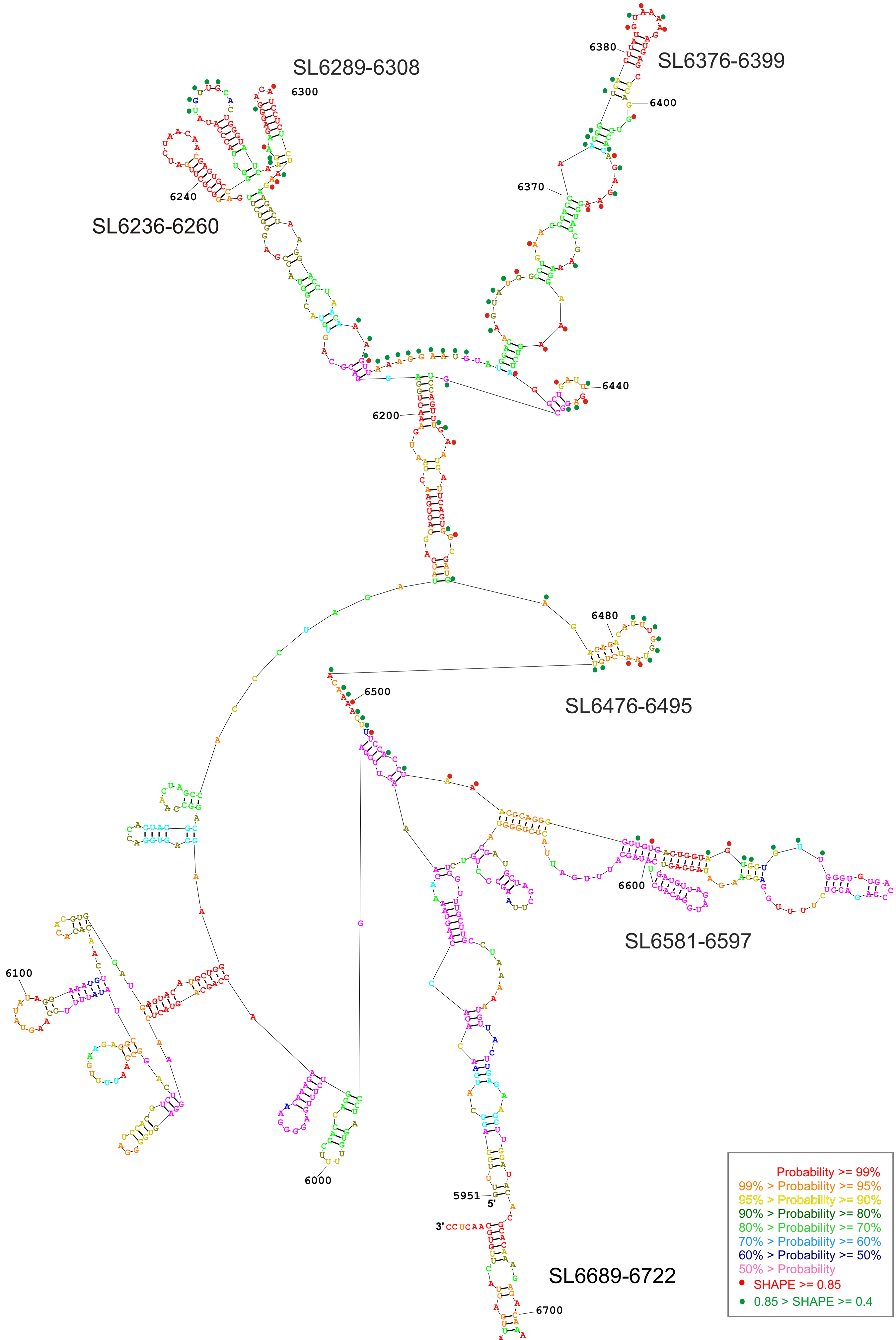

F. The structural model of F9 RNA

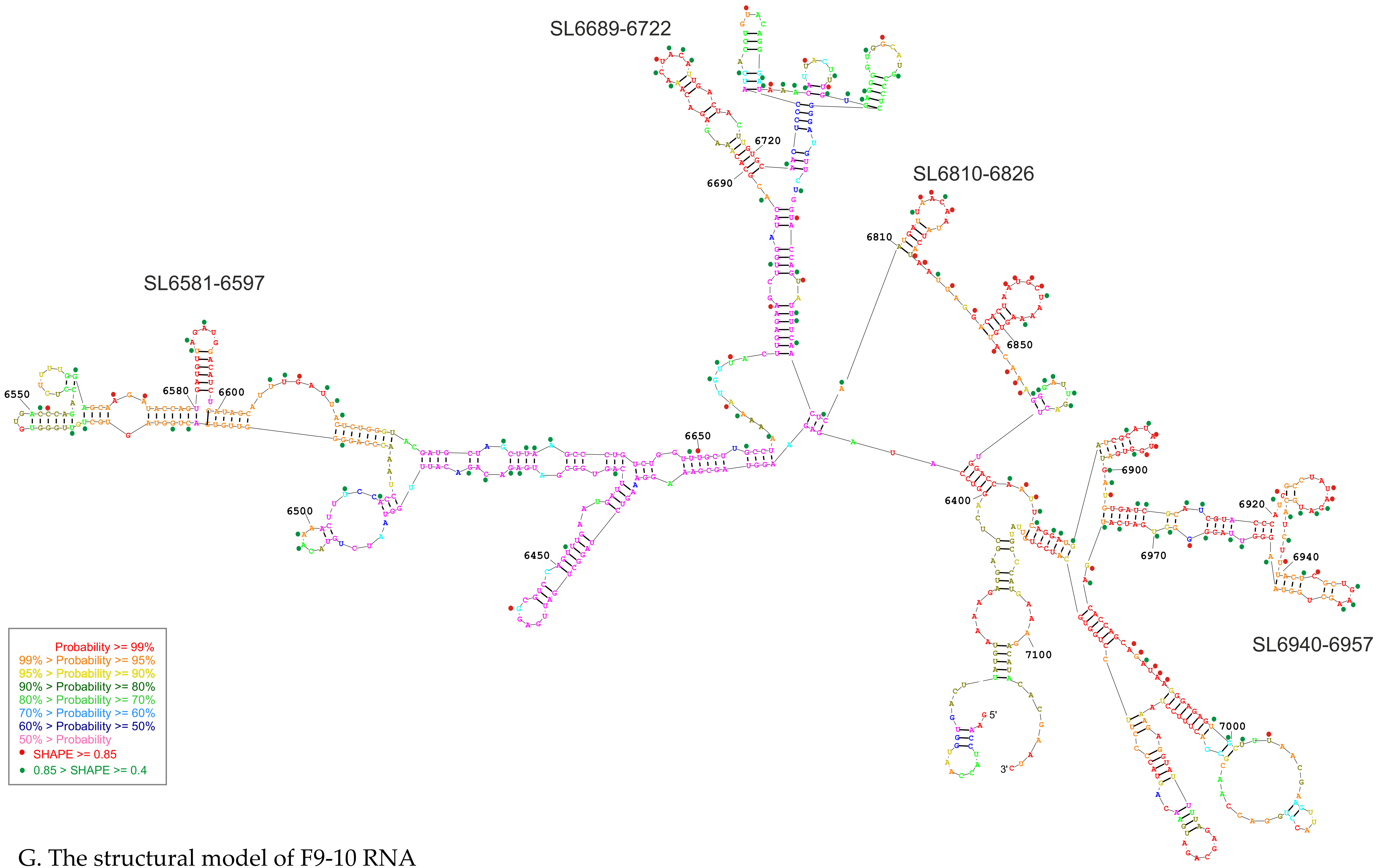

G. The structural model of F9-10 RNA

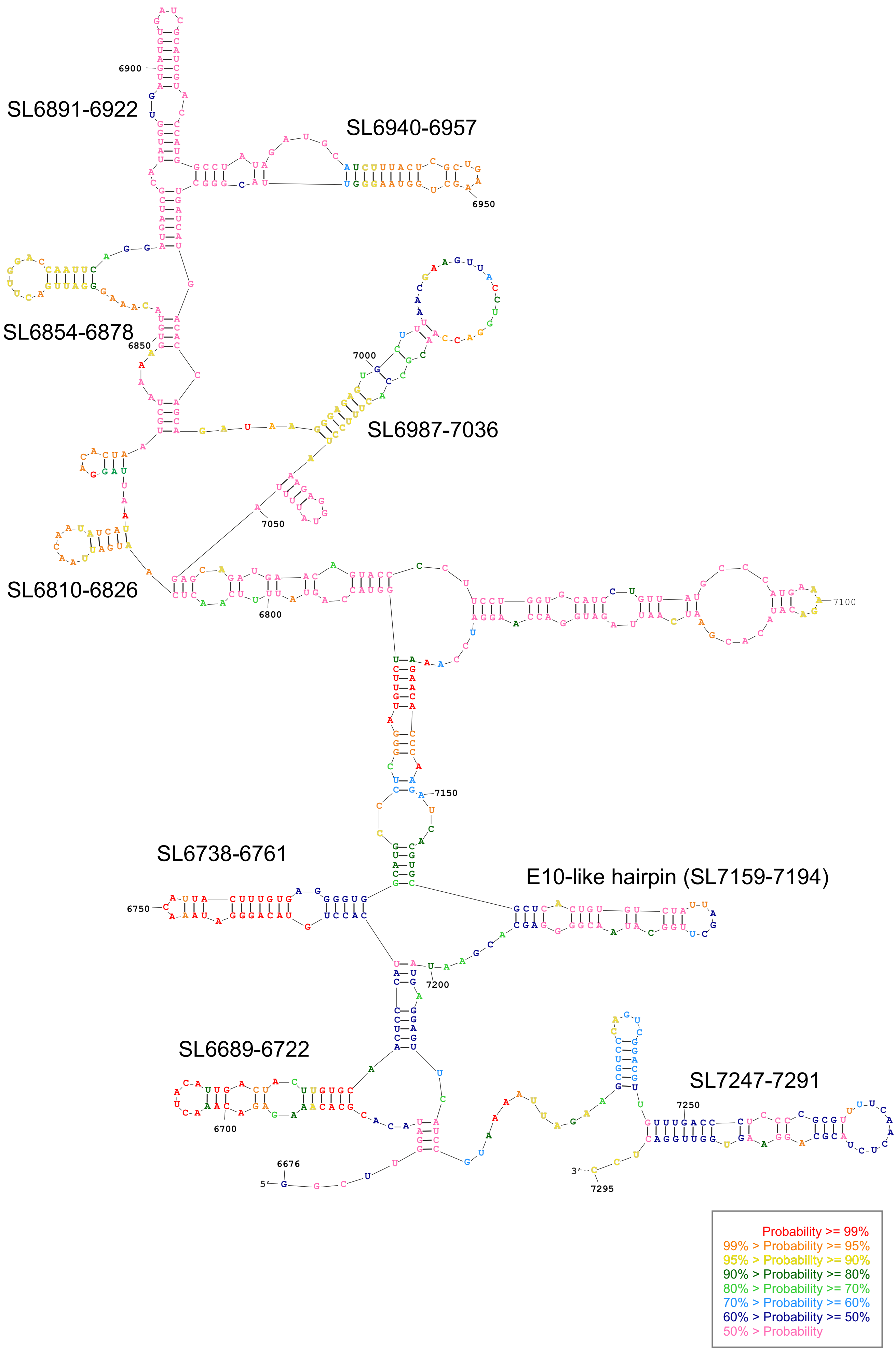

H. The structural model of F10 RNA (without terminal pseudoknot)
