# Supplementary material for: New RNA Structural Elements Identified in the Coding Region of the Coxsackie B3 Virus Genome
Source: Viruses. 2020 Oct 30;12(11):1232. doi: 10.3390/v12111232 (PMC7692623; doi:10.3390/v12111232)

Figure SM2. Consensus secondary structures and alingments generated by the *RNAalifold* program for particular RNA motifs identified in the coding part of the Coxsackie B3 virus genome, the "Nancy" strain and 19 other CVB3 isolates or for “Nancy” and other enterovirus strains. The colors annotate structure conservation according to the legend.

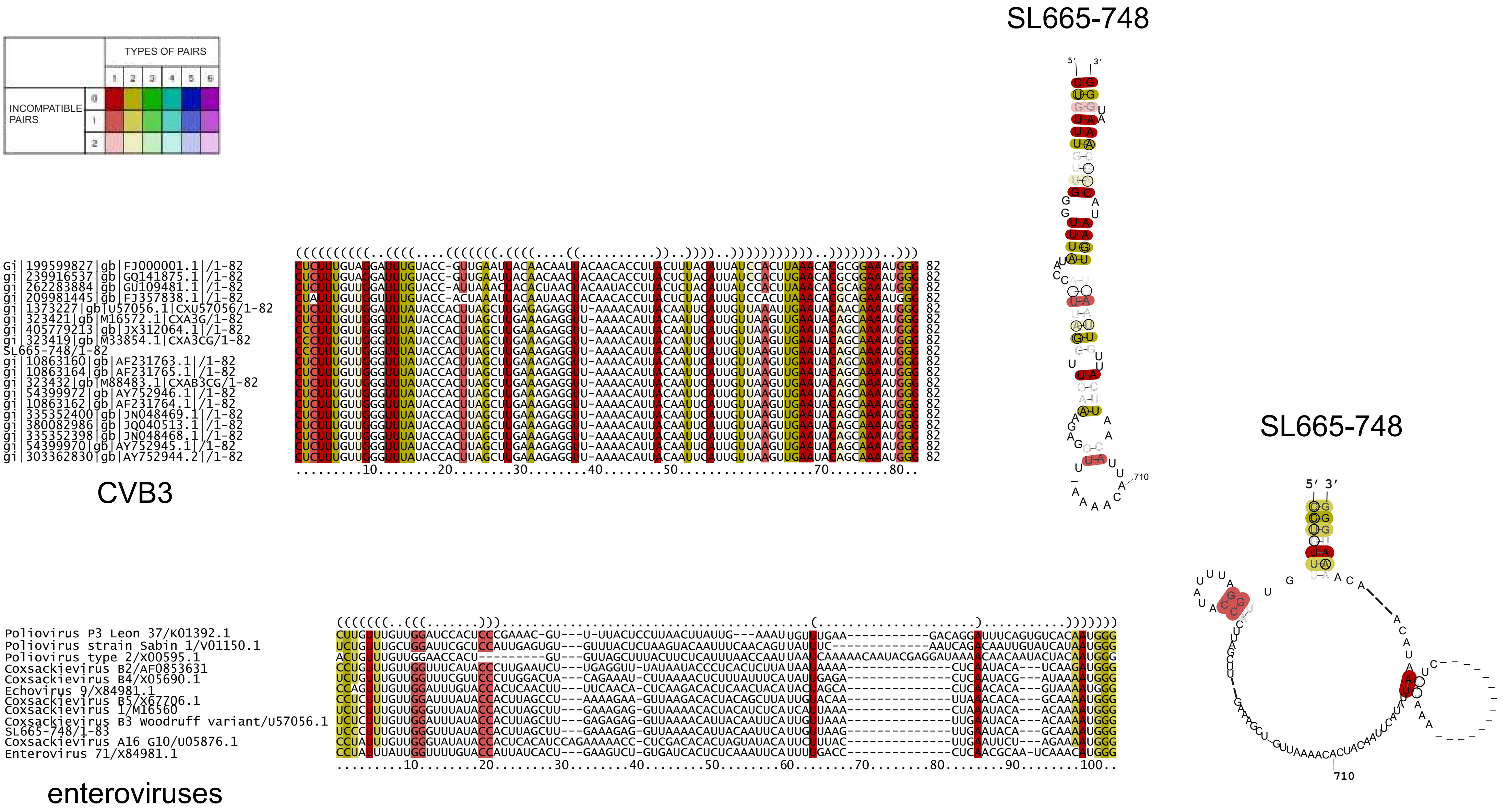

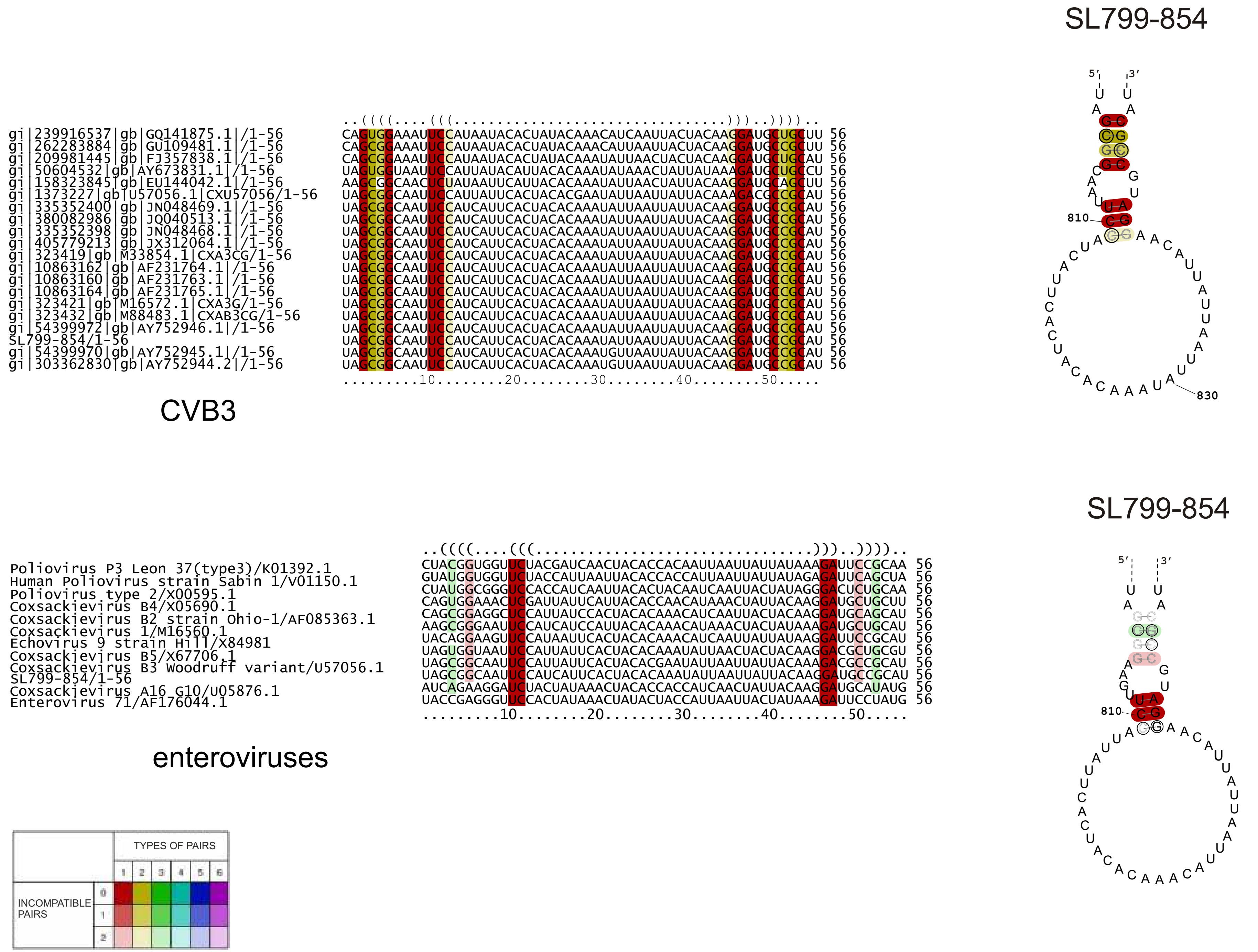
SL799-854
..((((....(((................................)))..))))..
gi|239916537|gb|GQ141875.1|/1-56 CAGUGGAAAUUCCAUAAUACACUAUACAAACAUCAAUUACUACAAGGAUGCUGCUU 56
gi|262283884|gb|GU109481.1|/1-56 CAGCGGAAAUUCCAUAAUACACUAUACAAACAUUAAUUACUACAAGGAUGCCGCUU 56
gi|209981445|gb|FJ357838.1|/1-56 CAGCGGAAAUUCCAUAAUACACUAUACAAAUAUUAACUACUACAAGGAUGCUGCAU 56
gi|50604532|gb|AY673831.1|/1-56 UAGUGGUAAUUCCAUUAUACAUUACACAAAUAUAAACUAUUAUAAAGAUGCUGCCU 56
gi|158323845|gb|EU144042.1|/1-56 AAGCGGCAACUCUAUAAUUCAUUACACAAAUAUUAACUAUUACAAGGAUGCAGCUU 56
gi|1373227|gb|U57056.1|CXU57056/1-56 UAGCGGCAAUUCCAUUAUUCACUACACGAAUAUUAAUUAUUACAAAGACGCCGCAU 56
gi|335352400|gb|JN048469.1|/1-56 UAGCGGCAAUUCCAUCAUUCACUACACAAAUAUUAAUUAUUACAAGGAUGCCGCAU 56
gi|380082986|gb|JQ040513.1|/1-56 UAGCGGCAAUUCCAUCAUUCACUACACAAAUAUUAAUUAUUACAAGGAUGCCGCAU 56
gi|335352398|gb|JN048468.1|/1-56 UAGCGGCAAUUCCAUCAUUCACUACACAAAUAUUAAUUAUUACAAGGAUGCCGCAU 56
gi|405779213|gb|JX312064.1|/1-56 UAGCGGCAAUUCCAUCAUUCACUACACAAAUAUUAAUUAUUACAAGGAUGCCGCAU 56
gi|323419|gb|M33854.1|CXA3CG/1-56 UAGCGGCAAUUCCAUCAUUCACUACACAAAUAUUAAUUAUUACAAGGAUGCCGCAU 56
gi|10863162|gb|AF231764.1|/1-56 UAGCGGCAAUUCCAUCAUUCACUACACAAAUAUUAAUUAUUACAAGGAUGCCGCAU 56
gi|10863160|gb|AF231763.1|/1-56 UAGCGGCAAUUCCAUCAUUCACUACACAAAUAUUAAUUAUUACAAGGAUGCCGCAU 56
gi|10863164|gb|AF231765.1|/1-56 UAGCGGCAAUUCCAUCAUUCACUACACAAAUAUUAAUUAUUACAAGGAUGCCGCAU 56
gi|323421|gb|M16572.1|CXA3G/1-56 UAGCGGCAAUUCCAUCAUUCACUACACAAAUAUUAAUUAUUACAAGGAUGCCGCAU 56
gi|323432|gb|M88483.1|CXAB3CG/1-56 UAGCGGCAAUUCCAUCAUUCACUACACAAAUAUUAAUUAUUACAAGGAUGCCGCAU 56
gi|54399972|gb|AY752946.1|/1-56 UAGCGGCAAUUCCAUCAUUCACUACACAAAUAUUAAUUAUUACAAGGAUGCCGCAU 56
SL799-854/1-56 UAGCGGCAAUUCCAUCAUUCACUACACAAAUAUUAAUUAUUACAAGGAUGCCGCAU 56
gi|54399970|gb|AY752945.1|/1-56 UAGCGGCAAUUCCAUCAUUCACUACACAAAUGUUAAUUAUUACAAGGAUGCCGCAU 56
gi|303362830|gb|AY752944.2|/1-56 UAGCGGCAAUUCCAUCAUUCACUACACAAAUGUUAAUUAUUACAAGGAUGCCGCAU 56
.........10........20........30........40........50.....
CVB3
5′ 3′
810
830
SL799-854
..((((....(((................................)))..))))..
Poliovirus P3 Leon 37(type3)/K01392.1 CUACGGUGGUUCUACGAUCAACUACACCACAAUUAAUUAUUAUAAAGAUUCCGCAA 56
Human Poliovirus strain Sabin 1/V01150.1 GUAUGGUGGUUCUACCAUUAAUUACACCACCAUUAAUUAUUAUAGAGAUUCAGCUA 56
Poliovirus type 2/X00595.1 CUAUGGCGGGUCCACCAUCAAUUACACUACAAUCAAUUACUAUAGGGACUCUGCAA 56
Coxsackievirus B4/X05690.1 CAGUGGAAACUCGAUUAUUCAUUACACCAACAUAAACUAUUACAAGGAUGCUGCUU 56
Coxsackievirus B2 strain Ohio-1/AF085363.1 CAGCGGAGGCUCCAUUAUCCACUACACAAACAUCAAUUACUACAAGGAUGCAGCAU 56
Coxsackievirus 1/M16560.1 AAGCGGGAAUUCCAUCAUCCAUUACACAAACAUAAACUACUAUAAAGAUGCUGCAU 56
Echovirus 9 strain Hill/X84981 UACAGGAAGUUCCAUAAUUCACUACACAAACAUCAAUUAUUAUAAGGAUUCCGCAU 56
Coxsackievirus B5/X67706.1 UAGUGGUAAUUCCAUUAUUCACUACACAAAUAUUAACUACUACAAGGACGCUGCGU 56
Coxsackievirus B3 Woodruff variant/U57056.1 UAGCGGCAAUUCCAUUAUUCACUACACGAAUAUUAAUUAUUACAAAGACGCCGCAU 56
SL799-854/1-56 UAGCGGCAAUUCCAUCAUUCACUACACAAAUAUUAAUUAUUACAAGGAUGCCGCAU 56
Coxsackievirus A16 G10/U05876.1 AUCAGAAGGAUCUACUAUAAACUACACCACCAUCAACUAUUACAAGGAUGCAUAUG 56
Enterovirus 71/AF176044.1 UACCGAGGGUUCCACUAUAAACUAUACUACCAUUAAUUACUAUAAAGAUUCCUAUG 56
.........10........20........30........40........50.....
enteroviruses
5′ 3′
810
TYPES OF PAIRS
1 2 3 4 5 6
INCOMPATIBLE PAIRS
0
1
2

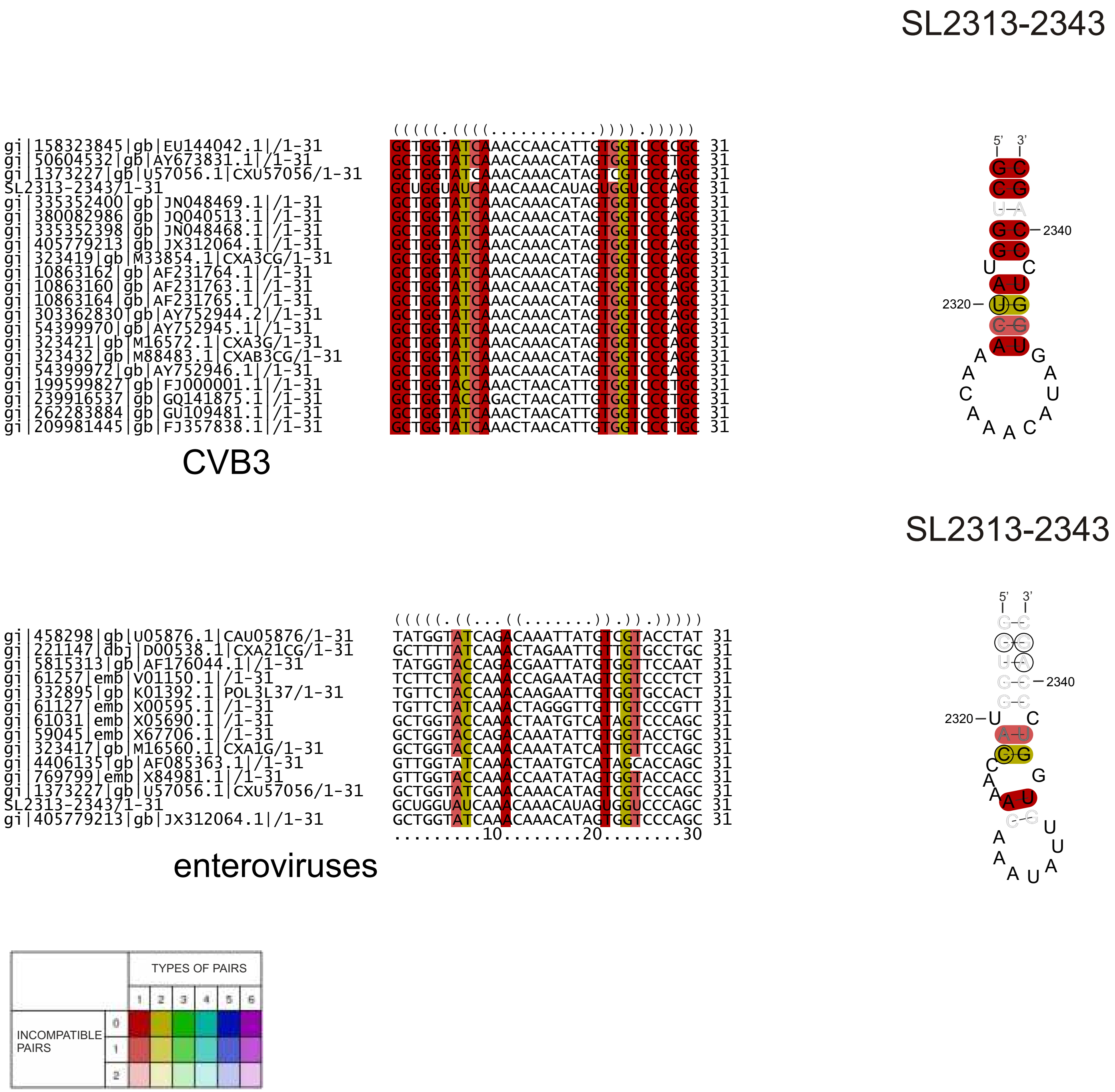

SL2313-2343
(((((.((((...........)))).)))))
gi|158323845|gb|EU144042.1|/1-31 GCTGGTATCAAACCAACATTGTGGTCCCCGC 31
gi|50604532|gb|AY673831.1|/1-31 GCTGGTATCAAACAAACATAGTGGTGCCTGC 31
gi|1373227|gb|U57056.1|CXU57056/1-31 GCTGGTATCAAACAAACATAGTCGTCCCAGC 31
SL2313-2343/1-31 GCUGGUAUCAAACAAACAUAGUGGUCCCAGC 31
gi|335352400|gb|JN048469.1|/1-31 GCTGGTATCAAACAAACATAGTGGTCCCAGC 31
gi|380082986|gb|JQ040513.1|/1-31 GCTGGTATCAAACAAACATAGTGGTCCCAGC 31
gi|335352398|gb|JN048468.1|/1-31 GCTGGTATCAAACAAACATAGTGGTCCCAGC 31
gi|405779213|gb|JX312064.1|/1-31 GCTGGTATCAAACAAACATAGTGGTCCCAGC 31
gi|323419|gb|M33854.1|CXA3CG/1-31 GCTGGTATCAAACAAACATAGTGGTCCCAGC 31
gi|10863162|gb|AF231764.1|/1-31 GCTGGTATCAAACAAACATAGTGGTCCCAGC 31
gi|10863160|gb|AF231763.1|/1-31 GCTGGTATCAAACAAACATAGTGGTCCCAGC 31
gi|10863164|gb|AF231765.1|/1-31 GCTGGTATCAAACAAACATAGTGGTCCCAGC 31
gi|303362830|gb|AY752944.2|/1-31 GCTGGTATCAAACAAACATAGTGGTCCCAGC 31
gi|54399970|gb|AY752945.1|/1-31 GCTGGTATCAAACAAACATAGTGGTCCCAGC 31
gi|323421|gb|M16572.1|CXA3G/1-31 GCTGGTATCAAACAAACATAGTGGTCCCAGC 31
gi|323432|gb|M88483.1|CXAB3CG/1-31 GCTGGTATCAAACAAACATAGTGGTCCCAGC 31
gi|54399972|gb|AY752946.1|/1-31 GCTGGTATCAAACAAACATAGTGGTCCCAGC 31
gi|199599827|gb|FJ000001.1|/1-31 GCTGGTACCAAACTAACATTGTGGTCCCTGC 31
gi|239916537|gb|GQ141875.1|/1-31 GCTGGTACCAGACTAACATTGTGGTCCCTGC 31
gi|262283884|gb|GU109481.1|/1-31 GCTGGTATCAAACTAACATTGTGGTCCCTGC 31
gi|209981445|gb|FJ357838.1|/1-31 GCTGGTATCAAACTAACATTGTGGTCCCTGC 31
CVB3
5' 3'
2340
2320
SL2313-2343
(((((.((...((.......)).)).)))))
gi|458298|gb|U05876.1|CAU05876/1-31 TATGGTATCAGACAAATTATGTCGTACCTAT 31
gi|221147|dbj|D00538.1|CXA21CG/1-31 GCTTTTATCAAACTAGAATTGTTGTGCCTGC 31
gi|5815313|gb|AF176044.1|/1-31 TATGGTACCAGACGAATTATGTGGTTCCAAT 31
gi|61257|emb|V01150.1|/1-31 TCTTCTACCAAACCAGAATAGTCGTCCCTCT 31
gi|332895|gb|K01392.1|POL3L37/1-31 TGTTCTACCAAACAAGAATTGTGGTGCCACT 31
gi|61127|emb|X00595.1|/1-31 TGTTCTATCAAACTAGGGTTGTTGTCCCGTT 31
gi|61031|emb|X05690.1|/1-31 GCTGGTACCAAACTAATGTCATAGTCCCAGC 31
gi|59045|emb|X67706.1|/1-31 GCTGGTACCAGACAAATATTGTGGTACCTGC 31
gi|323417|gb|M16560.1|CXA1G/1-31 GCTGGTACCAAACAAATATCATTGTTCCAGC 31
gi|4406135|gb|AF085363.1|/1-31 GTTGGTATCAAACTAATGTCATAGCACCAGC 31
gi|769799|emb|X84981.1|/1-31 GTTGGTACCAAACCAATATAGTGGTACCACC 31
gi|1373227|gb|U57056.1|CXU57056/1-31 GCTGGTATCAAACAAACATAGTCGTCCCAGC 31
SL2313-2343/1-31 GCUGGUAUCAAACAAACAUAGUGGUCCCAGC 31
gi|405779213|gb|JX312064.1|/1-31 GCTGGTATCAAACAAACATAGTGGTCCCAGC 31
.........10........20........30
enteroviruses
5' 3'
2340
2320
TYPES OF PAIRS
1 2 3 4 5 6
INCOMPATIBLE PAIRS
0
1
2

SL2562-2590

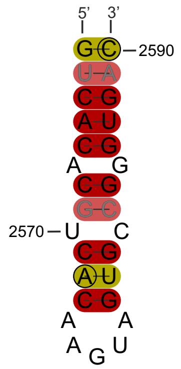


```
                                              (((((.((.(((.....))).)).)))))
gi|50604532|gb|AY673831.1|/1-29               GGCACACTTCACAGGTGGTGCCGAGTGAT 29
gi|1373227|gb|U57056.1|CXU57056/1-29          GTCACACGTCGCAAGTAGTGCCGAGTGAC 29
gi|335352400|gb|JN048469.1|/1-29              GTCACACGTCACAAGTAGTGCCGGGTGAC 29
gi|380082986|gb|JQ040513.1|/1-29              GTCACACGTCACAAGTAGTGCCGGGTGAC 29
gi|335352398|gb|JN048468.1|/1-29              GTCACACGTCACAAGTAGTGCCGGGTGAC 29
gi|405779213|gb|JX312064.1|/1-29              GTCACACGTCACAAGTAGTGCCGGGTGAC 29
gi|323419|gb|M33854.1|CXA3CG/1-29             GTCACACGTCACAAGTAGTGCCGGGTGAC 29
gi|10863162|gb|AF231764.1|/1-29               GTCACACGTCACAAGTAGTGCCGGGTGAC 29
gi|10863160|gb|AF231763.1|/1-29               GTCACACGTCACAAGTAGTGCCGGGTGAC 29
gi|10863164|gb|AF231765.1|/1-29               GTCACACGTCACAAGTAGTGCCGGGTGAC 29
gi|303362830|gb|AY752944.2|/1-29              GTCACACGTCACAAGTAGTGCCGGGTGAC 29
gi|54399970|gb|AY752945.1|/1-29               GTCACACGTCACAAGTAGTGCCGGGTGAC 29
gi|323421|gb|M16572.1|CXA3G/1-29              GTCACACGTCACAAGTAGTGCCGGGTGAC 29
gi|323432|gb|M88483.1|CXAB3CG/1-29            GTCACACGTCACAAGTAGTGCCGGGTGAC 29
gi|54399972|gb|AY752946.1|/1-29               GTCACACGTCACAAGTAGTGCCGGGTGAC 29
SL2562-2590/1-29                              GUCACACGUCACAAGUAGUGCCGGGUGAC 29
                                              .........10........20........
```

CVB3

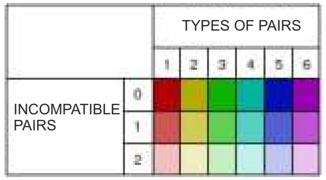

SL2664-2719

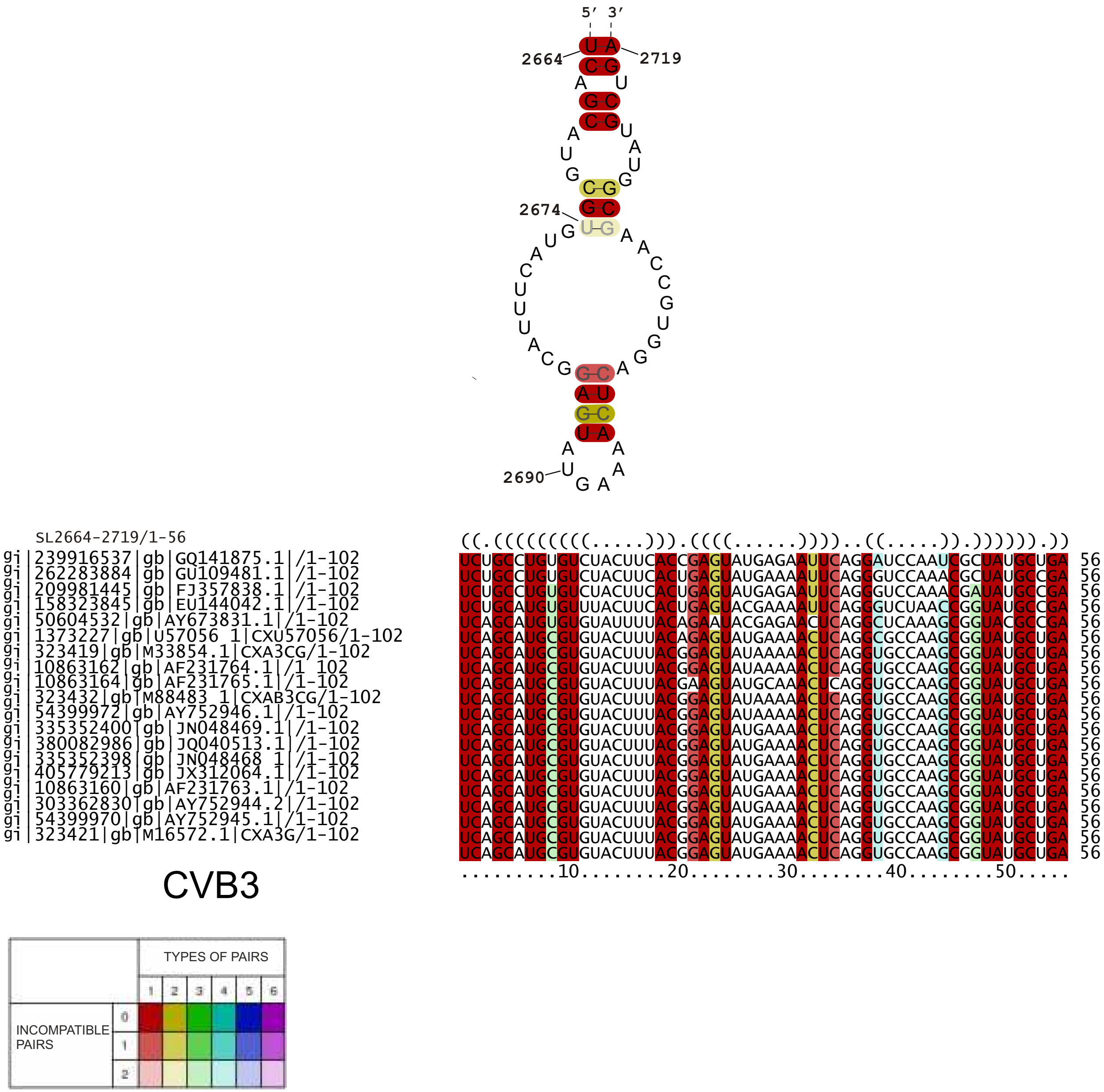

CVB3

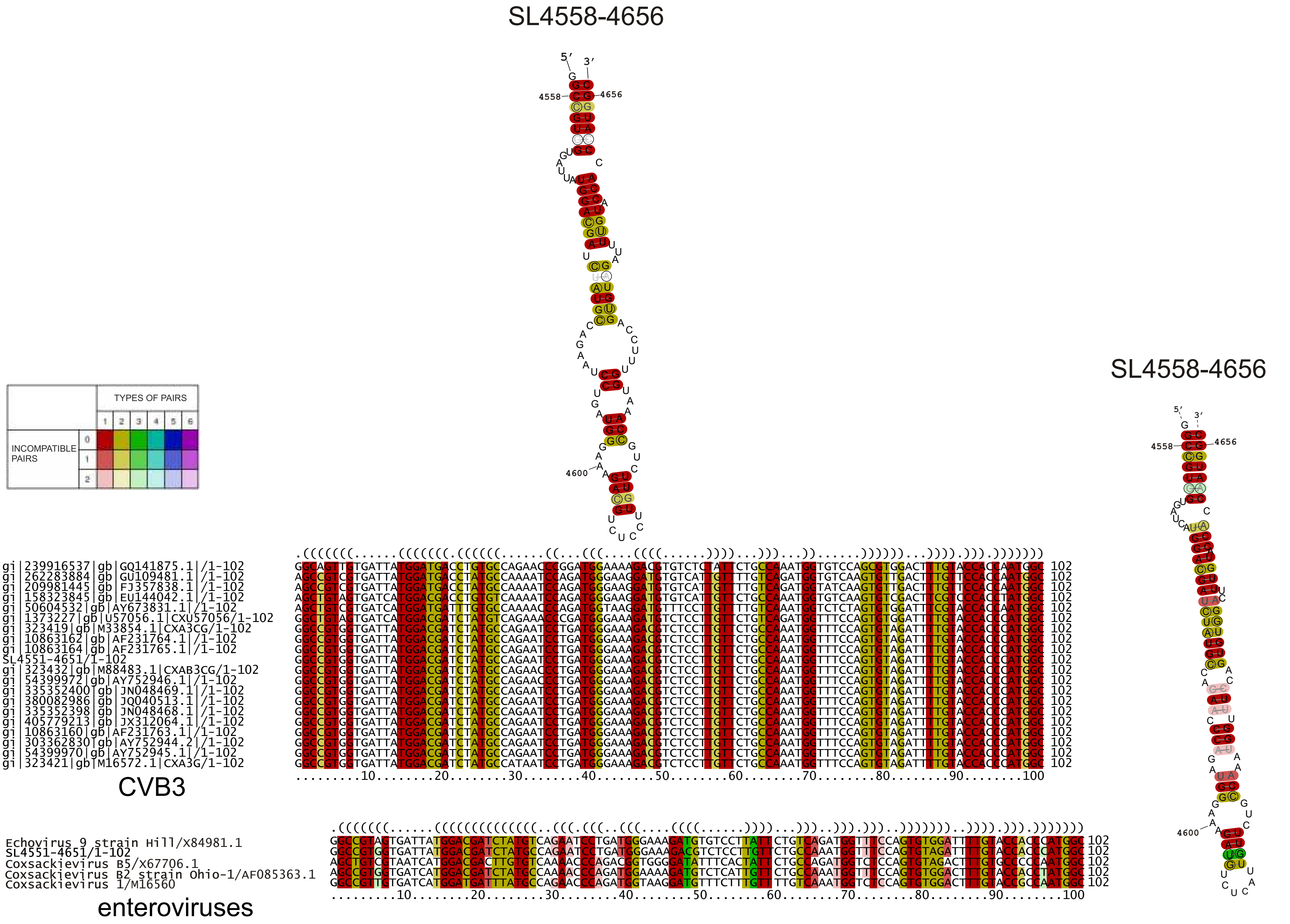

SL4558-4656
SL4558-4656
TYPES OF PAIRS
INCOMPATIBLE PAIRS
gi|239916537|gb|GQ141875.1|/1-102
gi|262283884|gb|GU109481.1|/1-102
gi|209981445|gb|FJ357838.1|/1-102
gi|158323845|gb|EU144042.1|/1-102
gi|50604532|gb|AY673831.1|/1-102
gi|1373227|gb|U57056.1|CXU57056/1-102
gi|323419|gb|M33854.1|CXA3CG/1-102
gi|10863162|gb|AF231764.1|/1-102
gi|10863164|gb|AF231765.1|/1-102
SL4551-4651/1-102
gi|323432|gb|M88483.1|CXAB3CG/1-102
gi|54399972|gb|AY752946.1|/1-102
gi|335352400|gb|JN048469.1|/1-102
gi|380082986|gb|JQ040513.1|/1-102
gi|335352398|gb|JN048468.1|/1-102
gi|405779213|gb|JX312064.1|/1-102
gi|10863160|gb|AF231763.1|/1-102
gi|303362830|gb|AY752944.2|/1-102
gi|54399970|gb|AY752945.1|/1-102
gi|323421|gb|M16572.1|CXA3G/1-102
CVB3
Echovirus 9 strain Hill/X84981.1
SL4551-4651/1-102
Coxsackievirus B5/X67706.1
Coxsackievirus B2 strain Ohio-1/AF085363.1
Coxsackievirus 1/M16560
enteroviruses

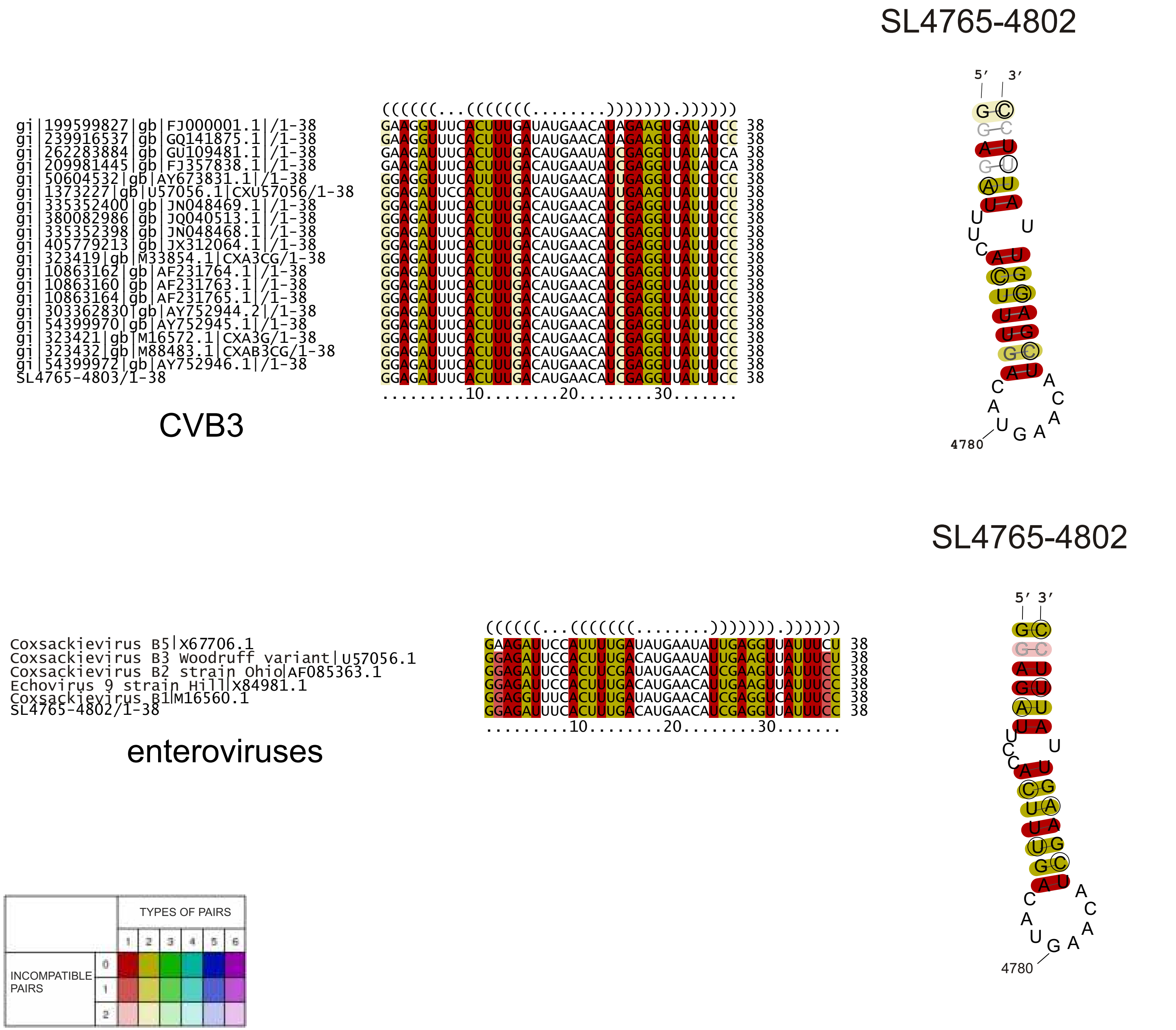
SL4765-4802
5′ 3′
4780
((((((...(((((((........))))))).))))))
gi|199599827|gb|FJ000001.1|/1-38 GAAGGUUUCACUUUGAUAUGAACAUAGAAGUGAUAUCC 38
gi|239916537|gb|GQ141875.1|/1-38 GAAGGUUUCACUUUGAUAUGAACAUAGAAGUGAUAUCC 38
gi|262283884|gb|GU109481.1|/1-38 GAAGAUUUCACUUUGACAUGAAUAUCGAGGUUAUAUCA 38
gi|209981445|gb|FJ357838.1|/1-38 GAAGAUUUCACUUUGACAUGAAUAUCGAGGUUAUAUCA 38
gi|50604532|gb|AY673831.1|/1-38 GGAGGUUUCAUUUUGAUAUGAACAUUGAGGUCAUCUCC 38
gi|1373227|gb|U57056.1|CXU57056/1-38 GGAGAUUCCACUUUGACAUGAAUAUUGAAGUUAUUUCU 38
gi|335352400|gb|JN048469.1|/1-38 GGAGAUUUCACUUUGACAUGAACAUCGAGGUUAUUUCC 38
gi|380082986|gb|JQ040513.1|/1-38 GGAGAUUUCACUUUGACAUGAACAUCGAGGUUAUUUCC 38
gi|335352398|gb|JN048468.1|/1-38 GGAGAUUUCACUUUGACAUGAACAUCGAGGUUAUUUCC 38
gi|405779213|gb|JX312064.1|/1-38 GGAGAUUUCACUUUGACAUGAACAUCGAGGUUAUUUCC 38
gi|323419|gb|M33854.1|CXA3CG/1-38 GGAGAUUUCACUUUGACAUGAACAUCGAGGUUAUUUCC 38
gi|10863162|gb|AF231764.1|/1-38 GGAGAUUUCACUUUGACAUGAACAUCGAGGUUAUUUCC 38
gi|10863160|gb|AF231763.1|/1-38 GGAGAUUUCACUUUGACAUGAACAUCGAGGUUAUUUCC 38
gi|10863164|gb|AF231765.1|/1-38 GGAGAUUUCACUUUGACAUGAACAUCGAGGUUAUUUCC 38
gi|303362830|gb|AY752944.2|/1-38 GGAGAUUUCACUUUGACAUGAACAUCGAGGUUAUUUCC 38
gi|54399970|gb|AY752945.1|/1-38 GGAGAUUUCACUUUGACAUGAACAUCGAGGUUAUUUCC 38
gi|323421|gb|M16572.1|CXA3G/1-38 GGAGAUUUCACUUUGACAUGAACAUCGAGGUUAUUUCC 38
gi|323432|gb|M88483.1|CXAB3CG/1-38 GGAGAUUUCACUUUGACAUGAACAUCGAGGUUAUUUCC 38
gi|54399972|gb|AY752946.1|/1-38 GGAGAUUUCACUUUGACAUGAACAUCGAGGUUAUUUCC 38
SL4765-4803/1-38 GGAGAUUUCACUUUGACAUGAACAUCGAGGUUAUUUCC 38
.........10........20........30.......
CVB3
SL4765-4802
5′ 3′
4780
((((((...(((((((........))))))).))))))
Coxsackievirus B5|X67706.1 GAAGAUUCCAUUUUGAUAUGAAUAUUGAGGUUAUUUCU 38
Coxsackievirus B3 Woodruff variant|U57056.1 GGAGAUUCCACUUUGACAUGAAUAUUGAAGUUAUUUCU 38
Coxsackievirus B2 strain Ohio|AF085363.1 GGAGAUUCCACUUCGAUAUGAACAUCGAAGUUAUUUCC 38
Echovirus 9 strain Hill|X84981.1 GGAGAUUCCACUUUGACAUGAACAUUGAAGUUAUUUCC 38
Coxsackievirus B1|M16560.1 GGAGGUUUCACUUUGAUAUGAACAUCGAGGUCAUUUCC 38
SL4765-4802/1-38 GGAGAUUUCACUUUGACAUGAACAUCGAGGUUAUUUCC 38
.........10........20........30.......
enteroviruses
TYPES OF PAIRS
1 2 3 4 5 6
INCOMPATIBLE PAIRS
0
1
2

SL6236-6260

```
                                            ((((((((......))...))))))
gi|239916537|gb|GQ141875.1|/1-19            GGCCCTCGATCTGACGACTAGCGCA
gi|262283884|gb|GU109481.1|/1-19            AGCGCTTGACCTGACCACCAGTGCA
gi|209981445|gb|FJ357838.1|/1-19            AGCGCTTGACCTGACCACCAGTGCA
gi|50604532|gb|AY673831.1|/1-19             AGCCCTCGACCTGACAACAAGTGCA
gi|158323845|gb|EU144042.1|/1-19            GGCACTAGATTTGACTACAAGTGCA
gi|1373227|gb|U57056.1|CXU57056/1-19        GGCTCTTGATCTAACAACGAGTGCA
gi|323421|gb|M16572.1|CXA3G/1-19            GGCGCTTGATCTTACAACGAGTGCC
SL6236-6260/1-25                            GGCGCTTGATCTAACAACGAGTGCC
gi|335352400|gb|JN048469.1|/1-19            GGCGCTTGATCTAACAACGAGTGCC
gi|380082986|gb|JQ040513.1|/1-19            GGCGCTTGATCTAACAACGAGTGCC
gi|335352398|gb|JN048468.1|/1-19            GGCGCTTGATCTAACAACGAGTGCC
gi|405779213|gb|JX312064.1|/1-19            GGCGCTTGATCTAACAACGAGTGCC
gi|323419|gb|M33854.1|CXA3CG/1-19           GGCGCTTGATCTAACAACGAGTGCC
gi|10863162|gb|AF231764.1|/1-19             GGCGCTTGATCTAACAACGAGTGCC
gi|10863160|gb|AF231763.1|/1-19             GGCGCTTGATCTAACAACGAGTGCT
gi|10863164|gb|AF231765.1|/1-19             GGCGCTTGATCTAACAACGAGTGCT
gi|303362830|gb|AY752944.2|/1-19            GGCGCTTGATCTAACAACGAGTGCC
gi|54399970|gb|AY752945.1|/1-19             GGCGCTTGATCTAACAACGAGTGCC
gi|323432|gb|M88483.1|CXAB3CG/1-19          GGCGCTTGATCTAACAACGAGTGCC
gi|54399972|gb|AY752946.1|/1-19             GGCGCTTGATCTAACAACGAGTGCC
                                            .........10........20....
```

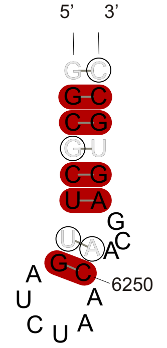

CVB3

SL6236-6260

```
                                            .((.((.............)).)).
gi|221147|dbj|D00538.1|CXA21CG/1-25         AGCTCTGGATCTTAGCACTAGTGCT 25
gi|332895|gb|K01392.1|POL3L37/1-25          GGCGCTAGATCTGTCTACCAGTGCC 25
gi|61127|emb|X00595.1|/1-25                 AGCACTTGACTTGACCACTAGTGCT 25
gi|61257|emb|V01150.1|/1-25                 AGCACTTGATTTGTCCACCAGTGCT 25
gi|59045|emb|X67706.1|/1-25                 AGCTCTCGATTTAACAACAAGCGCA 25
gi|4406135|gb|AF085363.1|/1-25              AGCTCTTGATTTAACAACGAGTGCA 25
gi|61031|emb|X05690.1|/1-25                 GGCTCTTGATTTAACAACAAGTGCG 25
gi|323417|gb|M16560.1|CXA1G/1-25            GGCTCTTGATTTAACAACGAGTGCG 25
gi|769799|emb|X84981.1|/1-25                GGCTCTTGATTTAACAACGAGTGCG 25
gi|1373227|gb|U57056.1|CXU57056/1-25        GGCTCTTGATCTAACAACGAGTGCA 25
gi|405779213|gb|JX312064.1|/1-25            GGCGCTTGATCTAACAACGAGTGCC 25
gi|5815313|gb|AF176044.1|/1-25              AGCTATAGATCTTCACACAAGTGCA 25
gi|458298|gb|U05876.1|CAU05876/1-25         GGCCATTGATCTCCACACCAGCGCG 25
                                            .........10........20....
```

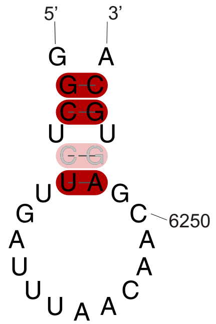

enteroviruses

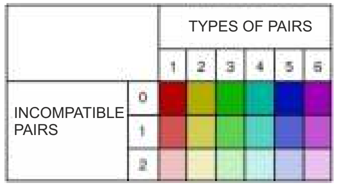

SL6289-6308

```
                                          ..(((((.....))))).
gi|239916537|gb|GQ141875.1|/1-18          AAAGAGAGACATTTTGTC 18
gi|262283884|gb|GU109481.1|/1-18          AAAGAGAGACATATTGTC 18
gi|209981445|gb|FJ357838.1|/1-18          AAAGAGAGACATATTGTC 18
gi|158323845|gb|EU144042.1|/1-18          GAAGAGAGACATTCTGTC 18
gi|50604532|gb|AY673831.1|/1-18           GAAGAGGGACATCCTTTC 18
gi|1373227|gb|U57056.1|CXU57056/1-18      GAAGAGAGACATCCTCTC 18
gi|335352400|gb|JN048469.1|/1-18          GAAGAGGGACATCCTCTC 18
gi|380082986|gb|JQ040513.1|/1-18          GAAGAGGGACATCCTCTC 18
gi|335352398|gb|JN048468.1|/1-18          GAAGAGGGACATCCTCTC 18
gi|405779213|gb|JX312064.1|/1-18          GAAGAGGGACATCCTCTC 18
gi|323419|gb|M33854.1|CXA3CG/1-18         GAAGAGGGACATCCTCTC 18
gi|10863162|gb|AF231764.1|/1-18           GAAGAGGGACATCCTCTC 18
gi|10863160|gb|AF231763.1|/1-18           GAAGAGGGACATCCTCTC 18
gi|10863164|gb|AF231765.1|/1-18           GAAGAGGGACATCCTCTC 18
gi|303362830|gb|AY752944.2|/1-18          GAAGAGGGACATCCTCTC 18
gi|54399970|gb|AY752945.1|/1-18           GAAGAGGGACATCCTCTC 18
gi|323421|gb|M16572.1|CXA3G/1-18          GAAGAGGGACATCCTCTC 18
gi|323432|gb|M88483.1|CXAB3CG/1-18        GAAGAGGGACATCCTCTC 18
gi|54399972|gb|AY752946.1|/1-18           GAAGAGGGACATCCTCTC 18
SL6290-6307                               GAAGAGGGACATCCTCTC 18
                                          .........10.......
```

CVB3

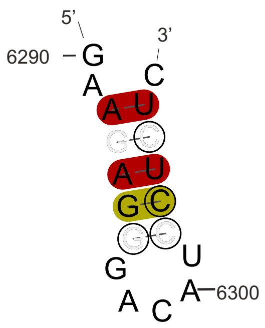

SL6289-6308

```
                                          ..((.((....))))...
gi|458298|gb|U05876.1|CAU05876/1-18       GAAGAGAGATATTTTTAGA 18
gi|5815313|gb|AF176044.1|/1-18            GAAAAAGGACATTTTTGGA 18
gi|332895|gb|K01392.1|POL3L37/1-18        GAAGAGAGATATCCTAAA 18
gi|61127|emb|X00595.1|/1-18               AAAGAGAGACATCTTGAA 18
gi|61257|emb|V01150.1|/1-18               GAAGAGAGATATCTTGAA 18
gi|59045|emb|X67706.1|/1-18               GAAGAGAGATATCCTCTC 18
gi|4406135|gb|AF085363.1|/1-18            GAAGAGAGATATCCTCTC 18
gi|323417|gb|M16560.1|CXA1G/1-18          AAAGAGGGACATCCTCTC 18
gi|1373227|gb|U57056.1|CXU57056/1-18      GAAGAGAGACATCCTCTC 18
gi|61031|emb|X05690.1|/1-18               GAAGAGGGACATCCTATC 18
gi|769799|emb|X84981.1|/1-18              AAAGAGGGACATCCTCTC 18
gi|405779213|gb|JX312064.1|/1-18          GAAGAGGGACATCCTCTC 18
                                          .........10.......
```

enteroviruses

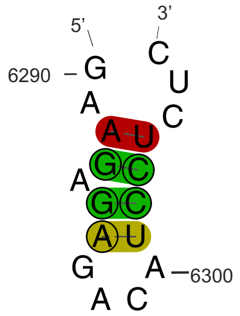

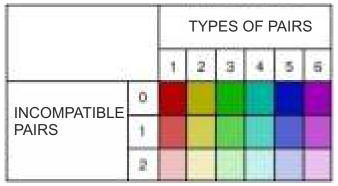

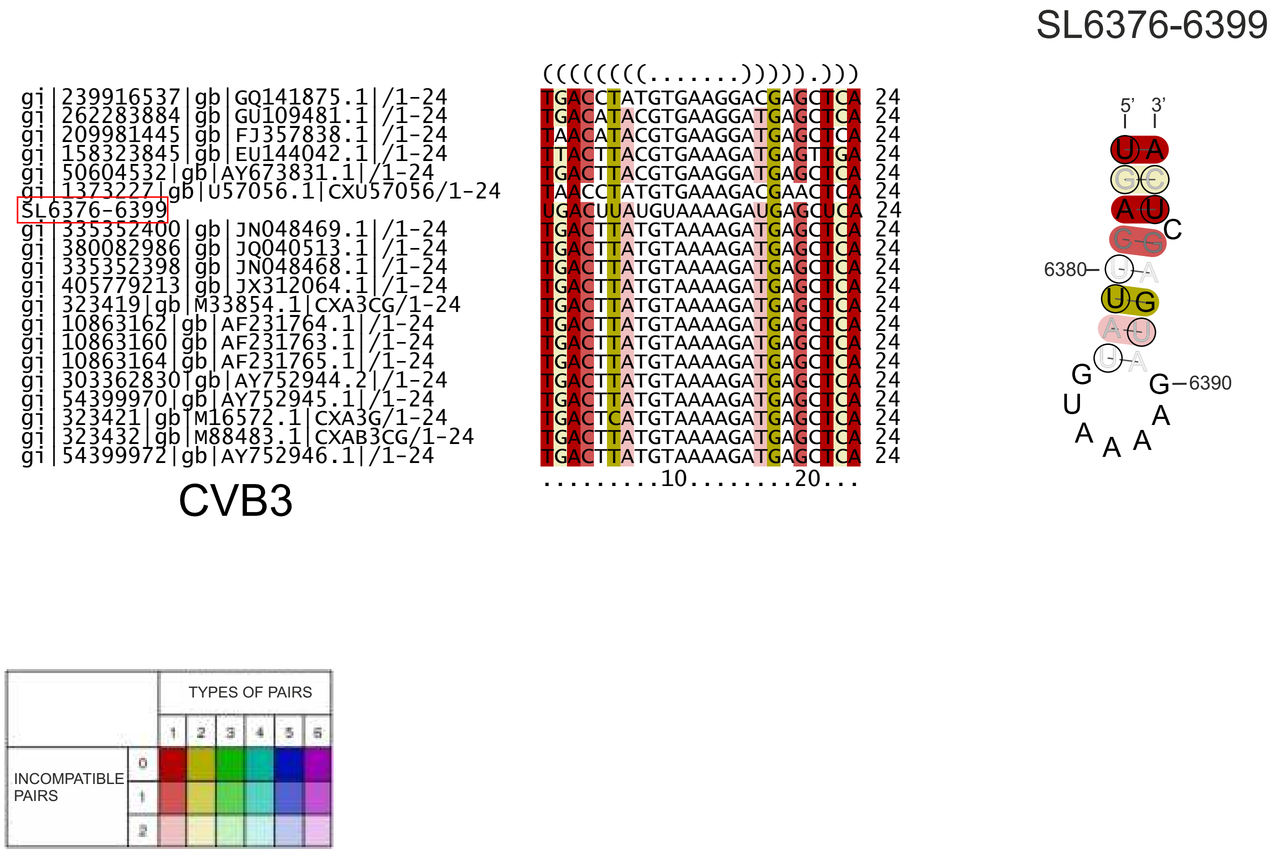

SL6376-6399
((((((((.......))))).)))
gi|239916537|gb|GQ141875.1|/1-24 TGACCTATGTGAAGGACGAGCTCA 24
gi|262283884|gb|GU109481.1|/1-24 TGACATACGTGAAGGATGAGCTCA 24
gi|209981445|gb|FJ357838.1|/1-24 TAACATACGTGAAGGATGAGCTCA 24
gi|158323845|gb|EU144042.1|/1-24 TTACTTACGTGAAAGATGAGTTGA 24
gi|50604532|gb|AY673831.1|/1-24 TGACTTACGTGAAGGATGAGCTCA 24
gi|1373227|gb|U57056.1|CXU57056/1-24 TAACCTATGTGAAAGACGAACTCA 24
SL6376-6399 UGACUUAUGUAAAAGAUGAGCUCA 24
gi|335352400|gb|JN048469.1|/1-24 TGACTTATGTAAAAGATGAGCTCA 24
gi|380082986|gb|JQ040513.1|/1-24 TGACTTATGTAAAAGATGAGCTCA 24
gi|335352398|gb|JN048468.1|/1-24 TGACTTATGTAAAAGATGAGCTCA 24
gi|405779213|gb|JX312064.1|/1-24 TGACTTATGTAAAAGATGAGCTCA 24
gi|323419|gb|M33854.1|CXA3CG/1-24 TGACTTATGTAAAAGATGAGCTCA 24
gi|10863162|gb|AF231764.1|/1-24 TGACTTATGTAAAAGATGAGCTCA 24
gi|10863160|gb|AF231763.1|/1-24 TGACTTATGTAAAAGATGAGCTCA 24
gi|10863164|gb|AF231765.1|/1-24 TGACTTATGTAAAAGATGAGCTCA 24
gi|303362830|gb|AY752944.2|/1-24 TGACTTATGTAAAAGATGAGCTCA 24
gi|54399970|gb|AY752945.1|/1-24 TGACTTATGTAAAAGATGAGCTCA 24
gi|323421|gb|M16572.1|CXA3G/1-24 TGACTCATGTAAAAGATGAGCTCA 24
gi|323432|gb|M88483.1|CXAB3CG/1-24 TGACTTATGTAAAAGATGAGCTCA 24
gi|54399972|gb|AY752946.1|/1-24 TGACTTATGTAAAAGATGAGCTCA 24
.........10........20...
CVB3
5'
3'
6380
6390
TYPES OF PAIRS
INCOMPATIBLE PAIRS

```
                                                .((((((.....))...)))).
gi|239916537|gb|GQ141875.1|/1-22                GACAAACATTTGGTAACCTATA 22
gi|262283884|gb|GU109481.1|/1-22                GGCAAACCTTTGGTAACCTGTA 22
gi|209981445|gb|FJ357838.1|/1-22                GGCAGACCTTTGGCAACCTATA 22
gi|158323845|gb|EU144042.1|/1-22                GACAGACCTTTGGGAATTTATA 22
gi|50604532|gb|AY673831.1|/1-22                 GGCAGACATTCGGTAACCTATA 22
gi|1373227|gb|U57056.1|CXU57056/1-22            GACAGACATTCGGCAACTTGTA 22
SL6476-6495                                     GACAGACAUUUGGUAAUCUGUA 22
gi|335352400|gb|JN048469.1|/1-22                GACAAACATTTGGTAATCTGTA 22
gi|380082986|gb|JQ040513.1|/1-22                GACAGACATTTGGTAATCTGTA 22
gi|335352398|gb|JN048468.1|/1-22                GACAGACATTTGGTAATCTGTA 22
gi|405779213|gb|JX312064.1|/1-22                GACAGACATTTGGTAATCTGTA 22
gi|323419|gb|M33854.1|CXA3CG/1-22               GACAGACATTTGGTAATCTGTA 22
gi|10863162|gb|AF231764.1|/1-22                 GACAGACATTTGGTAATCTGTA 22
gi|10863160|gb|AF231763.1|/1-22                 GACAGACATTTGGTAATCTGTA 22
gi|10863164|gb|AF231765.1|/1-22                 GACAGACATTTGGTAATCTGTA 22
gi|303362830|gb|AY752944.2|/1-22                GACAGACATTTGGTAATCTGTA 22
gi|54399970|gb|AY752945.1|/1-22                 GACAGACATTTGGTAATCTGTA 22
gi|323421|gb|M16572.1|CXA3G/1-22                GACAGACATTTGGTAATCTGTA 22
gi|323432|gb|M88483.1|CXAB3CG/1-22              GACAGACATTTGGTAATCTGTA 22
gi|54399972|gb|AY752946.1|/1-22                 GACAGACATTTGGTAATCTGTA 22
                                                .........10........20.
```

CVB3

SL6476-6495

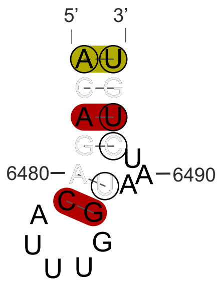


```
                                                .((((((.....))...)))).
gi|405779213|gb|JX312064.1|/1-22                GACAGACATTTGGTAATCTGTA 22
gi|4406135|gb|AF085363.1|/1-22                  GGCAGACGTTCGGCAATCTGTA 22
gi|1373227|gb|U57056.1|CXU57056/1-22            GACAGACATTCGGCAACTTGTA 22
gi|61031|emb|X05690.1|/1-22                     GGCAAACATTTGGTAATTTGTA 22
                                                .........10........20.
```

enteroviruses

SL6476-6495

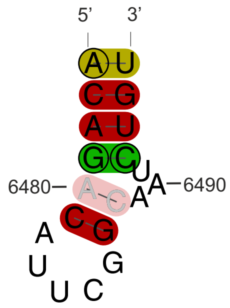

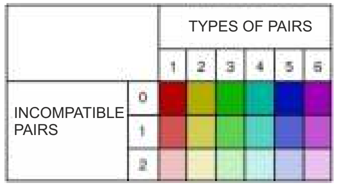

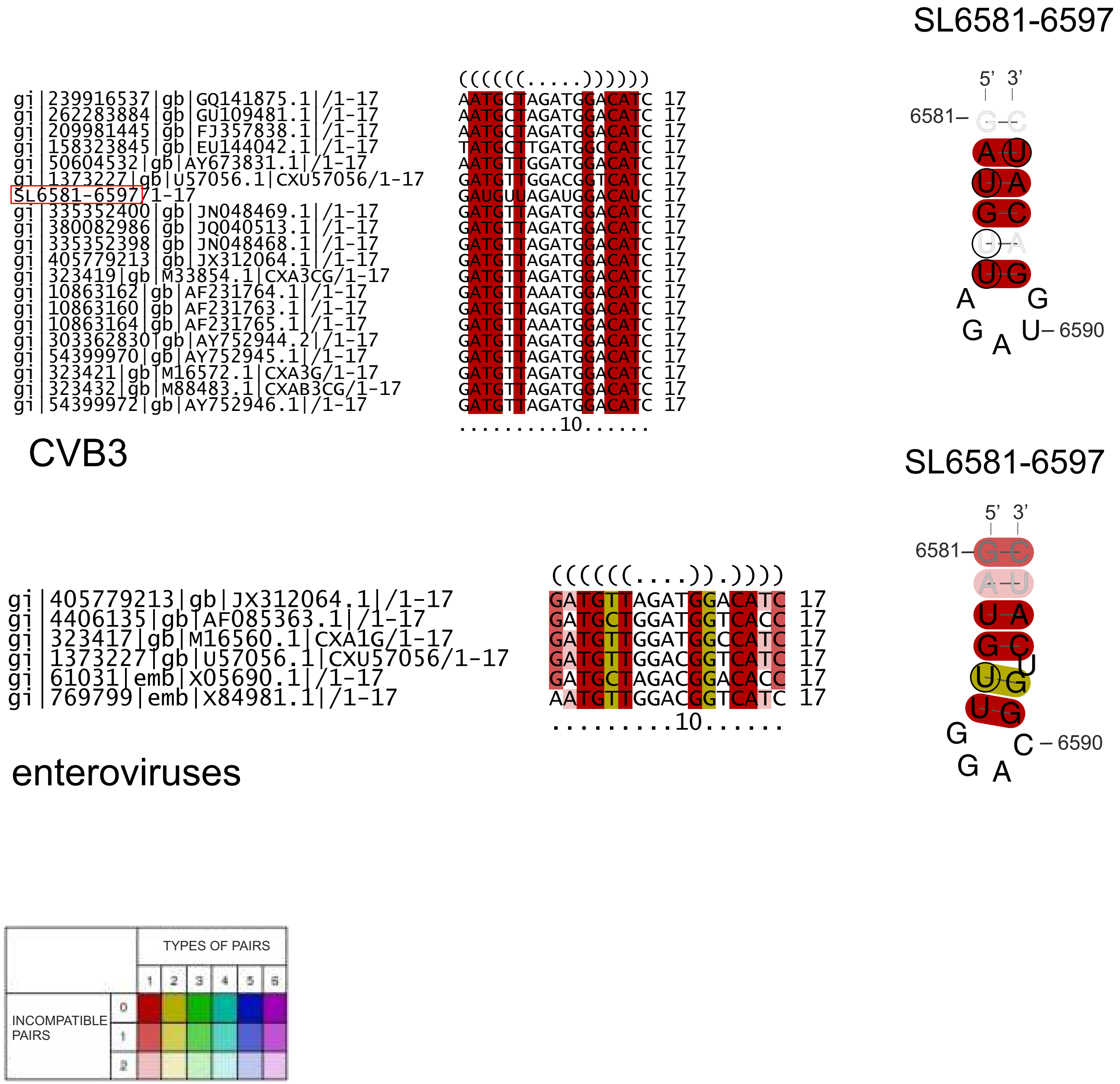
SL6581-6597
CVB3
((((((.....))))))
gi|239916537|gb|GQ141875.1|/1-17 AATGCTAGATGGACATC 17
gi|262283884|gb|GU109481.1|/1-17 AATGCTAGATGGACATC 17
gi|209981445|gb|FJ357838.1|/1-17 AATGCTAGATGGACATC 17
gi|158323845|gb|EU144042.1|/1-17 TATGCTTGATGGCCATC 17
gi|50604532|gb|AY673831.1|/1-17 AATGTTGGATGGACATC 17
gi|1373227|gb|U57056.1|CXU57056/1-17 GATGTTGGACGGTCATC 17
SL6581-6597/1-17 GAUGUUAGAUGGACAUC 17
gi|335352400|gb|JN048469.1|/1-17 GATGTTAGATGGACATC 17
gi|380082986|gb|JQ040513.1|/1-17 GATGTTAGATGGACATC 17
gi|335352398|gb|JN048468.1|/1-17 GATGTTAGATGGACATC 17
gi|405779213|gb|JX312064.1|/1-17 GATGTTAGATGGACATC 17
gi|323419|gb|M33854.1|CXA3CG/1-17 GATGTTAGATGGACATC 17
gi|10863162|gb|AF231764.1|/1-17 GATGTTAAATGGACATC 17
gi|10863160|gb|AF231763.1|/1-17 GATGTTAGATGGACATC 17
gi|10863164|gb|AF231765.1|/1-17 GATGTTAAATGGACATC 17
gi|303362830|gb|AY752944.2|/1-17 GATGTTAGATGGACATC 17
gi|54399970|gb|AY752945.1|/1-17 GATGTTAGATGGACATC 17
gi|323421|gb|M16572.1|CXA3G/1-17 GATGTTAGATGGACATC 17
gi|323432|gb|M88483.1|CXAB3CG/1-17 GATGTTAGATGGACATC 17
gi|54399972|gb|AY752946.1|/1-17 GATGTTAGATGGACATC 17
.........10......
5' 3'
6581–
6590
SL6581-6597
enteroviruses
((((((....)).))))
gi|405779213|gb|JX312064.1|/1-17 GATGTTAGATGGACATC 17
gi|4406135|gb|AF085363.1|/1-17 GATGCTGGATGGTCACC 17
gi|323417|gb|M16560.1|CXA1G/1-17 GATGTTGGATGGCCATC 17
gi|1373227|gb|U57056.1|CXU57056/1-17 GATGTTGGACGGTCATC 17
gi|61031|emb|X05690.1|/1-17 GATGCTAGACGGACACC 17
gi|769799|emb|X84981.1|/1-17 AATGTTGGACGGTCATC 17
.........10......
5' 3'
6581–
6590
TYPES OF PAIRS
1 2 3 4 5 6
INCOMPATIBLE PAIRS
0
1
2

```
                                                .(((((.((((..............)).))))))).
gi|158323845|gb|EU144042.1|/1-36                CCCACAAAGAAACCAACTACATAGATTACCTATGTA 36
gi|199599827|gb|FJ000001.1|/1-36                CACACAAAGAGACAAATTACATCGACTACTTGTGTA 36
gi|239916537|gb|GQ141875.1|/1-36                CACACAAAGAGACAAATTACATCGACTACTTGTGTA 36
gi|50604532|gb|AY673831.1|/1-36                 CACATAAGGAGACAAATTATATAGACTATCTCTGCA 36
gi|1373227|gb|U57056.1|CXU57056/1-36            CGCACAAGGAGACCAATTACATTGATTACCTGTGCA 36
gi|262283884|gb|GU109481.1|/1-36                CACACAAGGAGACTAATTACATCGATTATCTATGCA 36
gi|209981445|gb|FJ357838.1|/1-36                CACACAAGGAGACTAATTACATCGATTATCTATGCA 36
gi|10863164|gb|AF231765.1|/1-36                 CGCACAAAGAGACAAACTACATTGACTACTTGTGTA 36
gi|335352400|gb|JN048469.1|/1-36                CGCACAAAGAGACAAACTACATTGACTACTTGTGCA 36
gi|380082986|gb|JQ040513.1|/1-36                CGCACAAAGAGACAAACTACATTGACTACTTGTGCA 36
gi|335352398|gb|JN048468.1|/1-36                CGCACAAAGAGACAAACTACATTGACTACTTGTGCA 36
gi|405779213|gb|JX312064.1|/1-36                CGCACAAAGAGACAAACTACATTGACTACTTGTGCA 36
gi|323419|gb|M33854.1|CXA3CG/1-36               CGCACAAAGAGACAAACTACATTGACTACTTGTGCA 36
gi|10863162|gb|AF231764.1|/1-36                 CGCACAAAGAGACAAACTACATTGACTACTTGTGCA 36
gi|10863160|gb|AF231763.1|/1-36                 CGCACAAAGAGACAAACTACATTGACTACTTGTGCA 36
gi|303362830|gb|AY752944.2|/1-36                CGCACAAAGAGACAAACTACATTGACTACTTGTGCA 36
gi|54399970|gb|AY752945.1|/1-36                 CGCACAAAGAGACAAACTACATTGACTACTTGTGCA 36
gi|323421|gb|M16572.1|CXA3G/1-36                CGCACAAAGAGACAAACTACATTGACTACTTGTGCA 36
gi|323432|gb|M88483.1|CXAB3CG/1-36              CGCACAAAGAGACAAACTACATTGACTACTTGTGCA 36
gi|54399972|gb|AY752946.1|/1-36                 CGCACAAAGAGACAAACTACATTGACTACTTGTGCA 36
                                                .........10........20........30.....
```

CVB3

SL6689-6722

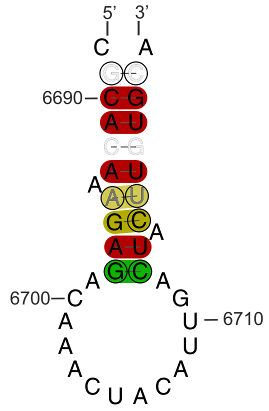


```
                                                .(((((.((((..((((...)))).)).))))))).
gi|59045|emb|X67706.1|/1-36                     CUCACAAAGAGACCAAUUAUAUUGACUAUUUGUGCA 36
gi|1373227|gb|U57056.1|CXU57056/1-36            CGCACAAGGAGACCAAUUACAUUGAUUACCUGUGCA 36
gi|61031|emb|X05690.1|/1-36                     CACAUAAAGAGACAAACUACAUUGACUACUUAUGCA 36
gi|405779213|gb|JX312064.1|/1-36                CGCACAAAGAGACAAACUACAUUGACUACUUGUGCA 36
gi|769799|emb|X84981.1|/1-36                    CGCACAAAGAGACCAAUUACAUUGACUACCUGUGUA 36
gi|323417|gb|M16560.1|CXA1G/1-36                CGCACAAGGAGACCAAUUACAUUGACUACCUGUGCA 36
                                                .........10........20........30.....
```

enteroviruses

SL6689-6722

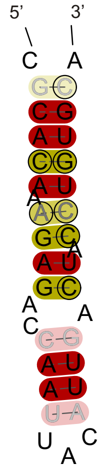

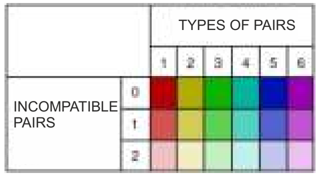

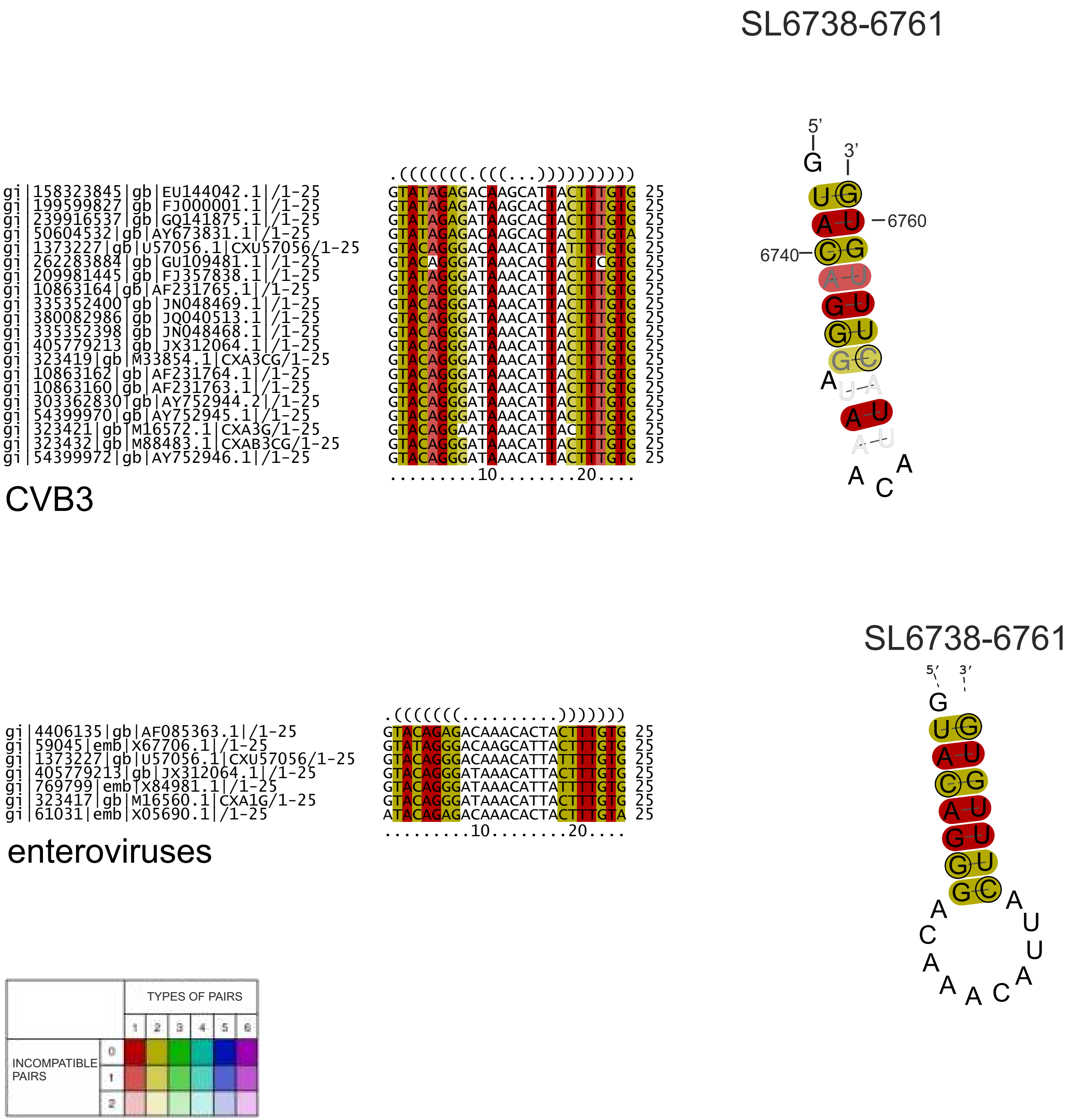
SL6738-6761
.(((((((.(((...))))))))))
gi|158323845|gb|EU144042.1|/1-25 GTATAGAGACAAGCATTACTTTGTG 25
gi|199599827|gb|FJ000001.1|/1-25 GTATAGAGATAAGCACTACTTTGTG 25
gi|239916537|gb|GQ141875.1|/1-25 GTATAGAGATAAGCACTACTTTGTG 25
gi|50604532|gb|AY673831.1|/1-25 GTATAGAGACAAGCACTACTTTGTA 25
gi|1373227|gb|U57056.1|CXU57056/1-25 GTACAGGGACAAACATTATTTTGTG 25
gi|262283884|gb|GU109481.1|/1-25 GTACAGGGATAAACACTACTTCGTG 25
gi|209981445|gb|FJ357838.1|/1-25 GTATAGGGATAAACATTACTTTGTG 25
gi|10863164|gb|AF231765.1|/1-25 GTACAGGGATAAACATTACTTTGTG 25
gi|335352400|gb|JN048469.1|/1-25 GTACAGGGATAAACATTACTTTGTG 25
gi|380082986|gb|JQ040513.1|/1-25 GTACAGGGATAAACATTACTTTGTG 25
gi|335352398|gb|JN048468.1|/1-25 GTACAGGGATAAACATTACTTTGTG 25
gi|405779213|gb|JX312064.1|/1-25 GTACAGGGATAAACATTACTTTGTG 25
gi|323419|gb|M33854.1|CXA3CG/1-25 GTACAGGGATAAACATTACTTTGTG 25
gi|10863162|gb|AF231764.1|/1-25 GTACAGGGATAAACATTACTTTGTG 25
gi|10863160|gb|AF231763.1|/1-25 GTACAGGGATAAACATTACTTTGTG 25
gi|303362830|gb|AY752944.2|/1-25 GTACAGGGATAAACATTACTTTGTG 25
gi|54399970|gb|AY752945.1|/1-25 GTACAGGGATAAACATTACTTTGTG 25
gi|323421|gb|M16572.1|CXA3G/1-25 GTACAGGAATAAACATTACTTTGTG 25
gi|323432|gb|M88483.1|CXAB3CG/1-25 GTACAGGGATAAACATTACTTTGTG 25
gi|54399972|gb|AY752946.1|/1-25 GTACAGGGATAAACATTACTTTGTG 25
.........10.........20....
CVB3
5'
3'
6760
6740
SL6738-6761
.(((((((...........)))))))
gi|4406135|gb|AF085363.1|/1-25 GTACAGAGACAAACACTACTTTGTG 25
gi|59045|emb|X67706.1|/1-25 GTATAGGGACAAGCATTACTTTGTG 25
gi|1373227|gb|U57056.1|CXU57056/1-25 GTACAGGGACAAACATTATTTTGTG 25
gi|405779213|gb|JX312064.1|/1-25 GTACAGGGATAAACATTACTTTGTG 25
gi|769799|emb|X84981.1|/1-25 GTACAGGGATAAACATTATTTTGTG 25
gi|323417|gb|M16560.1|CXA1G/1-25 GTACAGGGATAAACATTACTTTGTG 25
gi|61031|emb|X05690.1|/1-25 ATACAGAGACAAACACTACTTTGTA 25
.........10.........20....
enteroviruses
5'
3'
TYPES OF PAIRS
1 2 3 4 5 6
INCOMPATIBLE PAIRS
0
1
2

SL6809-6839

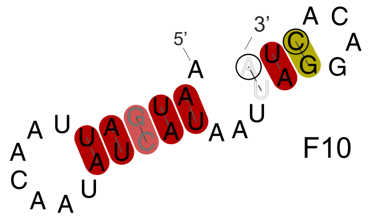

SL6810-6826

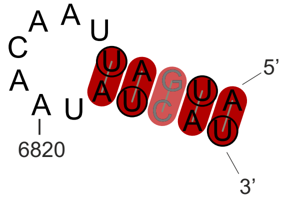


```
                                                .(((((.......)))))...(((....)))
gi|158323845|gb|EU144042.1|/1-30               CATGATT-ATAACATAATCATTAGAACATTA 30
gi|199599827|gb|FJ000001.1|/1-31               AATGATTAATAACATCATAATTAGAACACTG 31
gi|239916537|gb|GQ141875.1|/1-31               AATGATCAATAACATCATAATTAGAACACTG 31
gi|50604532|gb|AY673831.1|/1-31                GATGATAAACAACATCATAATTAGGACATTA 31
gi|1373227|gb|U57056.1|CXU57056/1-31           AATGATAAATAACATCATAATCAGGACACTC 31
gi|262283884|gb|GU109481.1|/1-31               AATGATCAACAACATCATAATCAGGACATTA 31
gi|209981445|gb|FJ357838.1|/1-31               AATGATCAACAACATCATAATCAGGACATTA 31
gi|10863164|gb|AF231765.1|/1-31                AATGATTAACAACATCATAATTAGGACACTA 31
gi|335352400|gb|JN048469.1|/1-31               AATGATTAACAATATCATAATTAGGACACTA 31
gi|380082986|gb|JQ040513.1|/1-31               AATGATTAACAATATCATAATTAGGACACTA 31
gi|335352398|gb|JN048468.1|/1-31               AATGATTAACAATATCATAATTAGGACACTA 31
gi|405779213|gb|JX312064.1|/1-31               AATGATTAACAATATCATAATTAGGACACTA 31
gi|323419|gb|M33854.1|CXA3CG/1-31              AATGATTAACAATATCATAATTAGGACACTA 31
gi|10863162|gb|AF231764.1|/1-31                AATGATTAACAATATCATAATTAGGACACTA 31
gi|10863160|gb|AF231763.1|/1-31                AATGATTAACAATATCATAATTAGGACACTA 31
gi|303362830|gb|AY752944.2|/1-31               AATGATTAACAATATCATAATTAGGACACTA 31
gi|54399970|gb|AY752945.1|/1-31                AATGATTAACAATATCATAATTAGGACACTA 31
gi|323421|gb|M16572.1|CXA3G/1-31               AATGATTAACAATATCATAATTAGGACACTA 31
gi|323432|gb|M88483.1|CXAB3CG/1-31             AATGATTAACAATATCATAATTAGGACACTA 31
gi|54399972|gb|AY752946.1|/1-31                AATGATTAACAATATCATAATTAGGACACTA 31
                                                .........10........20........30
```

CVB3

SL6809-6839

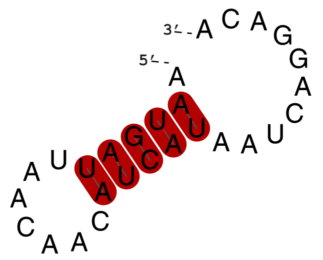


```
                                               .(((((.......))))).........
gi|458298|gb|U05876.1|CAU05876/1-28            AAUGAUCAACAACAUCAUAAUUAGGACA 28
gi|5815313|gb|AF176044.1|/1-28                 GAUGAUCAACAAUAUCAUUAUUAGAACA 28
gi|4406135|gb|AF085363.1|/1-28                 AAUGAUAAAUAACAUCAUCAUCAGGACU 28
gi|59045|emb|X67706.1|/1-28                    AAUGAUCAAUAACAUCAUAAUCAGGACU 28
gi|1373227|gb|U57056.1|CXU57056/1-28           AAUGAUAAAUAACAUCAUAAUCAGGACA 28
gi|61031|emb|X05690.1|/1-28                    AAUGAUCAAUAACAUCAUUAUCAGGACC 28
gi|405779213|gb|JX312064.1|/1-28               AAUGAUUAACAAUAUCAUAAUUAGGACA 28
gi|769799|emb|X84981.1|/1-28                   AAUGAUAAAUAACAUCAUAAUCAGGACA 28
gi|323417|gb|M16560.1|CXA1G/1-28               AAUGAUAAAUAACAUCAUAAUCAGGACA 28
                                               .........10........20.......
```

enteroviruses

SL6809-6857

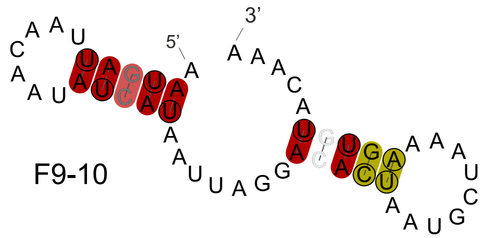


```
                                               .(((((.......))))).......(((((.........))))).....
gi|239916537|gb|GQ141875.1|/1-49               AATGATCAATAACATCATAATTAGAACACTGATGTTGAAGGTGTACAAG 49
gi|262283884|gb|GU109481.1|/1-49               AATGATCAACAACATCATAATCAGGACATTAATGTTGAAAGTTTACAAA 49
gi|209981445|gb|FJ357838.1|/1-49               AATGATCAACAACATCATAATCAGGACATTAATGTTGAAAGTTTACAAA 49
gi|158323845|gb|EU144042.1|/1-48               CATGATT-ATAACATAATCATTAGAACATTAATGCTGAAGGTGTACAAG 48
gi|50604532|gb|AY673831.1|/1-49                GATGATAAACAACATCATAATTAGGACATTAATGTTGAAGGTATACAAG 49
gi|1373227|gb|U57056.1|CXU57056/1-49           AATGATAAATAACATCATAATCAGGACACTCATGCTGAAGGTGTACAAA 49
SL6809-6857                                    AAUGAUUAACAAUAUCAUAAUUAGGACACUAAUGCUAAAAGUGUACAAA 49
gi|335352400|gb|JN048469.1|/1-49               AATGATTAACAATATCATAATTAGGACACTAATGCTAAAAGTGTACAAA 49
gi|380082986|gb|JQ040513.1|/1-49               AATGATTAACAATATCATAATTAGGACACTAATGCTAAAAGTGTACAAA 49
gi|335352398|gb|JN048468.1|/1-49               AATGATTAACAATATCATAATTAGGACACTAATGCTAAAAGTGTACAAA 49
gi|405779213|gb|JX312064.1|/1-49               AATGATTAACAATATCATAATTAGGACACTAATGCTAAAAGTGTACAAA 49
gi|323419|gb|M33854.1|CXA3CG/1-49              AATGATTAACAATATCATAATTAGGACACTAATGCTAAAAGTGTACAAA 49
gi|10863162|gb|AF231764.1|/1-49                AATGATTAACAATATCATAATTAGGACACTAATGCTAAAAGTGTACAAA 49
gi|10863160|gb|AF231763.1|/1-49                AATGATTAACAATATCATAATTAGGACACTAATGCTAAAAGTGTACAAA 49
gi|10863164|gb|AF231765.1|/1-49                AATGATTAACAACATCATAATTAGGACACTAATGCTAAAAGTGTACAAA 49
gi|303362830|gb|AY752944.2|/1-49               AATGATTAACAATATCATAATTAGGACACTAATGCTAAAAGTGTACAAA 49
gi|54399970|gb|AY752945.1|/1-49                AATGATTAACAATATCATAATTAGGACACTAATGCTAAAAGTGTACAAA 49
gi|323421|gb|M16572.1|CXA3G/1-49               AATGATTAACAATATCATAATTAGGACACTAATGCTAAAAGTGTACAAA 49
gi|323432|gb|M88483.1|CXAB3CG/1-49             AATGATTAACAATATCATAATTAGGACACTAATGCTAAAAGTGTACAAA 49
gi|54399972|gb|AY752946.1|/1-49                AATGATTAACAATATCATAATTAGGACACTAATGCTAAAAGTGTACAAA 49
                                               .........10........20........30........40........
```

CVB3

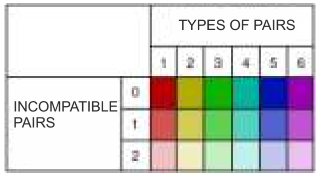

SL6854-6878

```
                                         .....((((((........))))))
gi|158323845|gb|EU144042.1|/1-25         CAAGGGGATAGACCTGGACCAGTTC 25
gi|199599827|gb|FJ000001.1|/1-25         CAAGGGCATTGACCTGGATCAGTTC 25
gi|239916537|gb|GQ141875.1|/1-25         CAAGGGCATTGACCTGGATCAGTTC 25
gi|50604532|gb|AY673831.1|/1-25          CAAGGGCATTGATTTGGACCAGTTC 25
gi|1373227|gb|U57056.1|CXU57056/1-25     CAAAGGGATCGACTTGGATCAATTC 25
gi|262283884|gb|GU109481.1|/1-25         CAAAGGCATTGACCTGGATCAATTT 25
gi|209981445|gb|FJ357838.1|/1-25         CAAAGGCATTGACCTGGATCAATTT 25
gi|10863164|gb|AF231765.1|/1-25          CAAAGGGATTGACTTGGACCAATTC 25
SL6854_6878/1-25                         CAAAGGGATTGACTTGGACCAATTC 25
gi|335352400|gb|JN048469.1|/1-25         CAAAGGGATTGACTTGGACCAATTC 25
gi|380082986|gb|JQ040513.1|/1-25         CAAAGGGATTGACTTGGACCAATTC 25
gi|335352398|gb|JN048468.1|/1-25         CAAAGGGATTGACTTGGACCAATTC 25
gi|405779213|gb|JX312064.1|/1-25         CAAAGGGATTGACTTGGACCAATTC 25
gi|323419|gb|M33854.1|CXA3CG/1-25        CAAAGGGATTGACTTGGACCAATTC 25
gi|10863162|gb|AF231764.1|/1-25          CAAAGGGATTGACTTGGACCAATTC 25
gi|10863160|gb|AF231763.1|/1-25          CAAAGGGATTGACTTGGACCAATTC 25
gi|303362830|gb|AY752944.2|/1-25         CAAAGGGATTGACTTGGACCAATTC 25
gi|54399970|gb|AY752945.1|/1-25          CAAAGGGATTGACTTGGACCAATTC 25
gi|323421|gb|M16572.1|CXA3G/1-25         CAAAGGGATTGACTTGGACCAATTC 25
gi|323432|gb|M88483.1|CXAB3CG/1-25       CAAAGGGATTGACTTGGACCAATTC 25
gi|54399972|gb|AY752946.1|/1-25          CAAAGGGATTGACTTGGACCAATTC 25
                                         .........10........20....
```

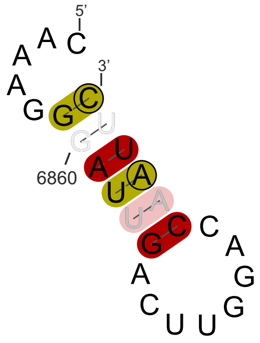

CVB3

SL6854-6878

```
                                             .....((((((........)))))).. 
SL6854-6878/-JX312064.1|/1-27                CAAAGGGATTGACTTGGACCAATTCAG 27
gi|4406135|gb|AF085363.1|/1-27               TAAGGGAATTGACTTGGACCAGTTTAG 27
gi|59045|emb|X67706.1|/1-27                  CAAGGGGATTGATTTGGACCAGTTCAG 27
gi|1373227|gb|U57056.1|CXU57056/1-27         CAAAGGGATCGACTTGGATCAATTCAG 27
gi|769799|emb|X84981.1|/1-27                 CAAAGGGATTGATTTGGATCAATTCAG 27
gi|323417|gb|M16560.1|CXA1G/1-27             CAAAGGGATCGACTTGGATCAATTCAG 27
                                             .........10........20......
```

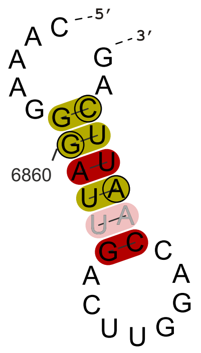

enteroviruses

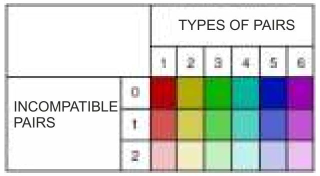

SL6891-6922

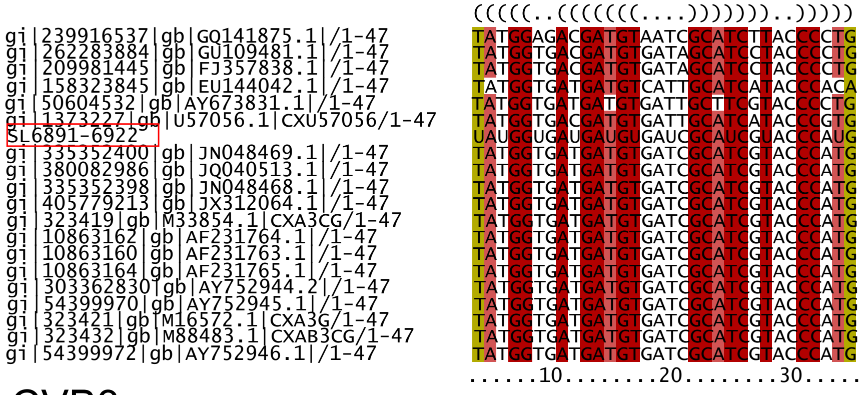

CVB3

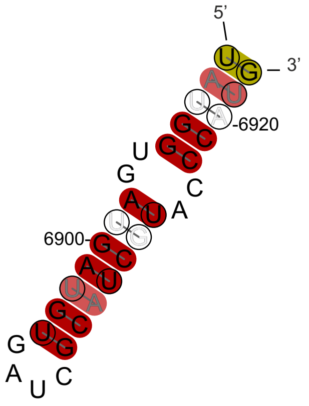

SL6891-6922

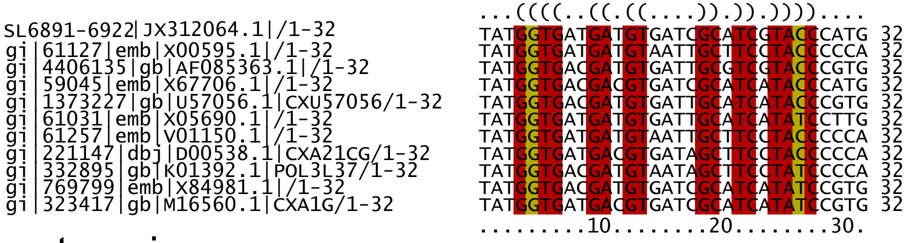

enteroviruses

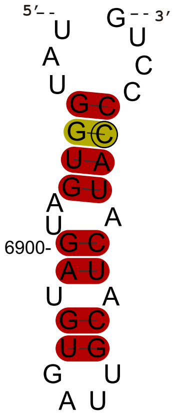

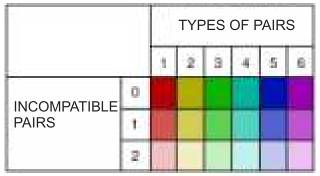

```
                                             .(((((.((....)).))))
gi|239916537|gb|GQ141875.1|/1-57              TTACTTGCTGAGGCGGGTAA
gi|262283884|gb|GU109481.1|/1-57              CTGTTGGCTGAAGCTGGTAA
gi|209981445|gb|FJ357838.1|/1-57              CTGTTAGCTGAAGCTGGTAA
gi|158323845|gb|EU144042.1|/1-57             CTGCTAGCTGAAGCTGGTAAA
gi|50604532|gb|AY673831.1|/1-57               CTACTAGCTGAAGCCGGTAA
gi|1373227|gb|U57056.1|CXU57056/1-57          TTGCTTGCTGAAGCTGGCAA
SL6940-6957                                   UUACUCGCUGAAGCUGGUAA
gi|335352400|gb|JN048469.1|/1-57              TTACTCGCTGAAGCTGGTAA
gi|380082986|gb|JQ040513.1|/1-57              TTACTCGCTGAAGCTGGTAA
gi|335352398|gb|JN048468.1|/1-57              TTACTCGCTGAAGCTGGTAA
gi|405779213|gb|JX312064.1|/1-57              TTACTCGCTGAAGCTGGTAA
gi|323419|gb|M33854.1|CXA3CG/1-57             TTACTCGCTGAAGCTGGTAA
gi|10863162|gb|AF231764.1|/1-57               TTACTCGCTGAAGCTGGTAA
gi|10863160|gb|AF231763.1|/1-57               TTACTCGCTGAAGCTGGTAA
gi|10863164|gb|AF231765.1|/1-57               TTACTCGCTGAAGCTGGTAA
gi|303362830|gb|AY752944.2|/1-57              TTACTCGCTGAAGCTGGTAA
gi|54399970|gb|AY752945.1|/1-57               TTACTCGCTGAAGCTGGTAA
gi|323421|gb|M16572.1|CXA3G/1-57              TTACTCGCTGAAGCTGGTAA
gi|323432|gb|M88483.1|CXAB3CG/1-57            TTACTCGCTGAAGCTGGTAA
gi|54399972|gb|AY752946.1|/1-57               TTACTCGCTGAAGCTGGTAA
                                              .........10........20
```

CVB3

SL6940-6957

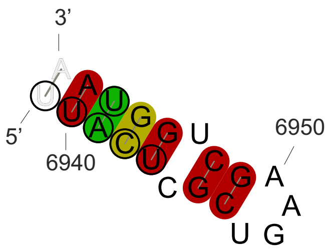


```
                                      (((((((.((....)).)))))))
SL6940-6957\JX312064.1|/1-24          CTTTACTCGCTGAAGCTGGTAAGG 24
gi|59045|emb|X67706.1|/1-24           CTCTGCTCGCTGAGGCTGGCAAGG 24
gi|1373227|gb|U57056.1|CXU57056/1-24  CTTTGCTTGCTGAAGCTGGCAAGG 24
gi|61031|emb|X05690.1|/1-24           CTCTGCTCGCTGAAGCTGGTAAAG 24
gi|769799|emb|X84981.1|/1-24          CCCTGCTTGCTGAAGCTGGTAAGG 24
gi|323417|gb|M16560.1|CXA1G/1-24      CCCTGCTTGCTGAAGCTGGTAGGG 24
                                      .........10........20...
```

enteroviruses

SL6940-6957

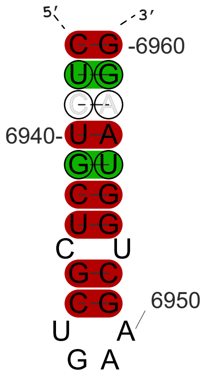

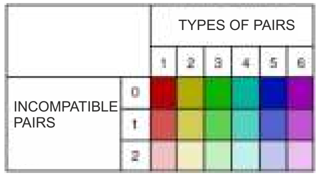

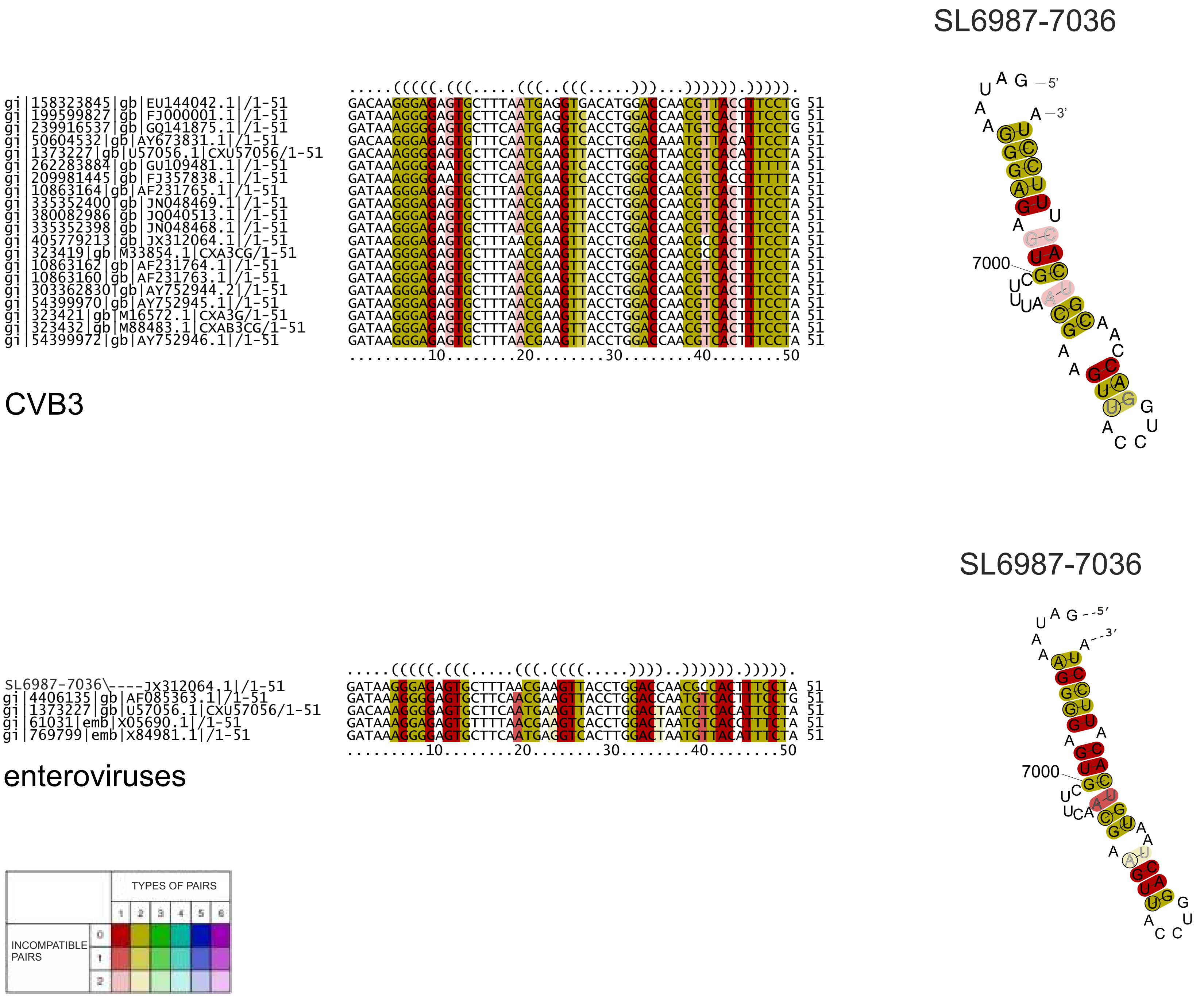

SL6987-7036
gi|158323845|gb|EU144042.1|/1-51
gi|199599827|gb|FJ000001.1|/1-51
gi|239916537|gb|GQ141875.1|/1-51
gi|50604532|gb|AY673831.1|/1-51
gi|1373227|gb|U57056.1|CXU57056/1-51
gi|262283884|gb|GU109481.1|/1-51
gi|209981445|gb|FJ357838.1|/1-51
gi|10863164|gb|AF231765.1|/1-51
gi|335352400|gb|JN048469.1|/1-51
gi|380082986|gb|JQ040513.1|/1-51
gi|335352398|gb|JN048468.1|/1-51
gi|405779213|gb|JX312064.1|/1-51
gi|323419|gb|M33854.1|CXA3CG/1-51
gi|10863162|gb|AF231764.1|/1-51
gi|10863160|gb|AF231763.1|/1-51
gi|303362830|gb|AY752944.2|/1-51
gi|54399970|gb|AY752945.1|/1-51
gi|323421|gb|M16572.1|CXA3G/1-51
gi|323432|gb|M88483.1|CXAB3CG/1-51
gi|54399972|gb|AY752946.1|/1-51
CVB3
7000
5'
3'
SL6987-7036
SL6987-7036\----JX312064.1|/1-51
gi|4406135|gb|AF085363.1|/1-51
gi|1373227|gb|U57056.1|CXU57056/1-51
gi|61031|emb|X05690.1|/1-51
gi|769799|emb|X84981.1|/1-51
enteroviruses
7000
TYPES OF PAIRS
INCOMPATIBLE PAIRS

SL7159-7194
E10-like hairpin

```
                                              ((((..((((((.........)))))).....))))
gi|158323845|gb|EU144042.1|/1-36              GATCCTTGTGCTTATTGGCTTGGCACAACGGAGAGC 36
gi|199599827|gb|FJ000001.1|/1-36              GATCCTTGTGCTTATTGGCTTGGCACAATGGAGAAC 36
gi|239916537|gb|GQ141875.1|/1-36              GATCCTTGTGCTTATTGGCTTGGCACAATGGGGAAC 36
gi|50604532|gb|AY673831.1|/1-36               GCTCATTGTGCCTATTGGCCTGGCACAATGGGGAGC 36
gi|1373227|gb|U57056.1|CXU57056/1-36          GCTCATTGTGCTTATTGGCCTGGCACAACGGGGAGC 36
gi|262283884|gb|GU109481.1|/1-36              GATCTTTGTGCCTATTGGCTTGGCACAATGGAGAGC 36
gi|209981445|gb|FJ357838.1|/1-36              GATCTTTGTGCCTATTGGCTTGGCACAATGGTGAGC 36
gi|10863164|gb|AF231765.1|/1-36               GCTCACTGTGTCTATTAGCTTGGCATAACGGGGAGC 36
gi|335352400|gb|JN048469.1|/1-36              GCTCACTGTGTCTATTAGCTTGGCATAACGGGGAGC 36
gi|380082986|gb|JQ040513.1|/1-36              GCTCACTGTGTCTATTAGCTTGGCATAACGGGGAGC 36
gi|335352398|gb|JN048468.1|/1-36              GCTCATTGTGTCTATTAGCTTGGCATAACGGGGAGC 36
gi|405779213|gb|JX312064.1|/1-36              GCTCACTGTGTCTATTAGCTTGGCATAACGGGGAGC 36
gi|323419|gb|M33854.1|CXA3CG/1-36             GCTCACTGTGTCTATTAGCTTGGCATAACGGGGAGC 36
gi|10863162|gb|AF231764.1|/1-36               GCTCACTGTGTCTATTAGCTTGGCATAACGGGGAGC 36
gi|10863160|gb|AF231763.1|/1-36               GCTCACTGTGTCTATTAGCTTGGCATAACGGGGAGC 36
gi|303362830|gb|AY752944.2|/1-36              GCTCACTGTGTCTATTAGCTTGGCATAACGGGGAGC 36
gi|54399970|gb|AY752945.1|/1-36               GCTCACTGTGTCTATTAGCTTGGCATAACGGGGAGC 36
gi|323421|gb|M16572.1|CXA3G/1-36              GCTCACTGTGTCTATTAGCTTGGCATAACGGGGAGC 36
gi|323432|gb|M88483.1|CXAB3CG/1-36            GCTCACTGTGTCTATTAGCTTGGCATAACGGGGAGC 36
gi|54399972|gb|AY752946.1|/1-36               GCTCACTGTGTCTATTAGCTTGGCATAACGGGGAGC 36
                                              .........10........20........30.....
```

CVB3

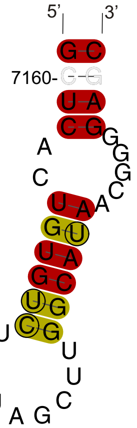

SL7159-7194
E10-like hairpin

```
                                              .(((..((((((.........))))))..)))....
SL7159-7194\JX312064.1/1-36                   GCTCACTGTGTCTATTAGCTTGGCATAACGGGGAGC 36
gi|61127|emb|X00595.1|/1-36                   GCTCATTGTGCCTATTGGCCTGGCACAACGGCGAAG 36
gi|458298|gb|U05876.1|CAU05876/1-36           GATCCCTGTGTCTTTTGGCGTGGCACAATGGTAAGC 36
gi|5815313|gb|AF176044.1|/1-36                GCTCCCTCTGCTTATTAGCATGGCACAACGGGAAAG 36
gi|4406135|gb|AF085363.1|/1-36                GCTCGCTGTGTCTATTGGCCTGGCACAATGGAGAGC 36
gi|59045|emb|X67706.1|/1-36                   GTTCATTGTGCTTATTGGCTTGGCACAATGGAGAAC 36
gi|1373227|gb|U57056.1|CXU57056/1-36          GCTCATTGTGCTTATTGGCCTGGCACAACGGGGAGC 36
gi|61031|emb|X05690.1|/1-36                   GCTCCCTGTGCTTATTGGCTTGGCACAATGGAGAGC 36
gi|61257|emb|V01150.1|/1-36                   GCTCTCTGTGCCTATTAGCTTGGCACAATGGCGAAG 36
gi|221147|dbj|D00538.1|CXA21CG/1-36           GCTCATTGTGCCTATTGGCTTGGCATAACGGCGAAG 36
gi|332895|gb|K01392.1|POL3L37/1-36            GCTCCTTGTGTCTATTGGCTTGGCACAACGGGGAAG 36
gi|769799|emb|X84981.1|/1-36                  GCTCGCTATGTCTATTGGCCTGGCACAACGGGGAGC 36
gi|323417|gb|M16560.1|CXA1G/1-36              GCTCGTTGTGCTTATTGGCCTGGCACAACGGGGAGC 36
                                              .........10........20........30.....
```

enteroviruses

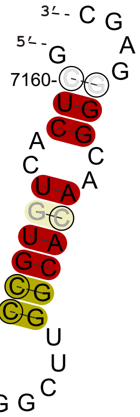

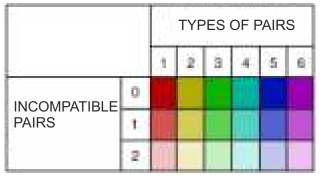

SL7247-7291

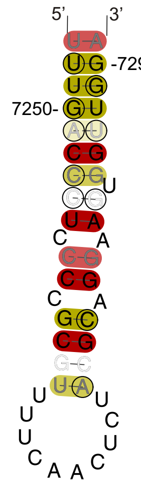


```
                                                    (((((((((.((.((((..........)))).)).)).)))))))
gi|158323845|gb|EU144042.1|/1-45                    CCTAACCTTGCCTGCATTTTCGACTCTACGCAGGAAGTGGCTAGA 45
gi|199599827|gb|FJ000001.1|/1-45                    TCTGGCCCTACCTGCGTTTTCGACCCTGCGCAGGAAATGGCTGGA 45
gi|239916537|gb|GQ141875.1|/1-45                    TCTGGCCCTACCTGCGTTTTCGGCCCTGCGCAGGAAATGGCTGGA 45
gi|50604532|gb|AY673831.1|/1-45                     TTTGACACTACCTGCATTCTCAACACTACGCAGAAAATGGTTGGA 45
gi|1373227|gb|U57056.1|CXU57056/1-45                TTTGACTCTACCTGCGTTCTCAACCATTCGTAGGAAGTGGTTGGA 45
gi|262283884|gb|GU109481.1|/1-45                    TCTGACTTTGCCTGCGTTTTCGACTTTGCGCAGGAAGTGGCTAGA 45
gi|209981445|gb|FJ357838.1|/1-45                    TCTGACTTTGCCTGCGTTTTCGACTTTGCGCAGGAAATGGTTAGA 45
gi|10863164|gb|AF231765.1|/1-45                     TTTGACCCTCCCCGCGTTTTCAACTCTACGCAGGAAGTGGTTGGA 45
gi|335352400|gb|JN048469.1|/1-45                    TTTGACCCTCCCCGCGTTTTCAACTCTACGCAGGAAGTGGTTGGA 45
gi|380082986|gb|JQ040513.1|/1-45                    TTTGACCCTCCCCGCGTTTTCAACTCTACGCAGGAAGTGGTTGGA 45
gi|335352398|gb|JN048468.1|/1-45                    TTTGACCCTCCCCGCGTTTTCAACTCTACGCAGGAAGTGGTTGGA 45
gi|405779213|gb|JX312064.1|/1-45                    TTTGACCCTCCCCGCGTTTTCAACTCTACGCAGGAAGTGGTTGGA 45
gi|323419|gb|M33854.1|CXA3CG/1-45                   TTTGACCCTCCCCGCGTTTTCAACTCTACGCAGGAAGTGGTTGGA 45
gi|10863162|gb|AF231764.1|/1-45                     TTTGACCCTCCCCGCGTTTTCAACTCTACGCAGGAAGTGGTTGGA 45
gi|10863160|gb|AF231763.1|/1-45                     TTTGACCCTCCCCGCGTTTTCAACTCTACGCAGGAAGTGGTTGGA 45
gi|303362830|gb|AY752944.2|/1-45                    TTTGACCCTCCCCGCGTTTTCAACTCTACGCAGGAAGTGGTTGGA 45
gi|54399970|gb|AY752945.1|/1-45                     TTTGACCCTCCCCGCGTTTTCAACTCTACGCAGGAAGTGGTTGGA 45
gi|323421|gb|M16572.1|CXA3G/1-45                    TTTGACCCTCCCCGCGTTTACAACTCTAGGCAGGAAGTGGTTGGA 45
gi|323432|gb|M88483.1|CXAB3CG/1-45                  TTTGACCCTCCCCGCGTTTTCAACTCTACGCAGGAAGTGGTTGGA 45
gi|54399972|gb|AY752946.1|/1-45                     TTTGACCCTCCCCGCGTTTTCAACTCTACGCAGGAAGTGGTTGGA 45
                                                    .........10........20........30........40....
```

CVB3

SL7247-7291

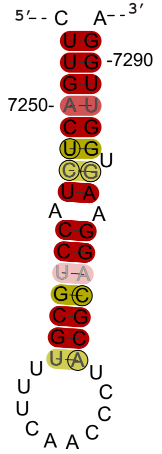


```
                                                    .((((((((.(((((((..........))))))).)).))))))).
gi|4406135|gb|AF085363.1|/1-45                      CUUGUCUCUGCCUGCGUUUUCGACCUUGCGCAGGAAGUGGUUGGA 45
gi|59045|emb|X67706.1|/1-45                         CUUGACCUUGCCUGCGUUUUCAACUUUACGCAGGAAAUGGUUGGA 45
gi|1373227|gb|U57056.1|CXU57056/1-45                UUUGACUCUACCUGCGUUCUCAACCAUUCGUAGGAAGUGGUUGGA 45
gi|61031|emb|X05690.1|/1-45                         CUUGACUCUGCCCGCGUUUUCGACCCUACGUAGGAAAUGGUUGGA 45
gi|SL572_616/1-45                                   UUUGACCCUCCCCGCGUUUUCAACUCUACGCAGGAAGUGGUUGGA 45
gi|769799|emb|X84981.1|/1-45                        UUUGACUCUACCUGCGUUUUCAACCCUACGCAGGAAGUGGUUGGA 45
gi|323417|gb|M16560.1|CXA1G/1-45                    CUUGACUCUACCUGCGUUUUCAACUCUACGCAGGAAGUGGUUGGA 45
                                                    .........10........20........30........40....
```

enteroviruses

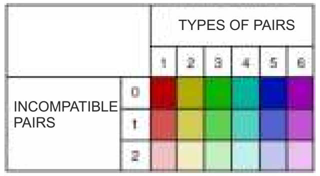

Supplement: Supplementary file 1 [file viruses-12-01232-s001.zip › SM2.pdf]
