# Supplementary material for: New RNA Structural Elements Identified in the Coding Region of the Coxsackie B3 Virus Genome
Source: Viruses. 2020 Oct 30;12(11):1232. doi: 10.3390/v12111232 (PMC7692623; doi:10.3390/v12111232)
Supplement: Supplementary file 1 [file viruses-12-01232-s001.zip › SM3.pdf]

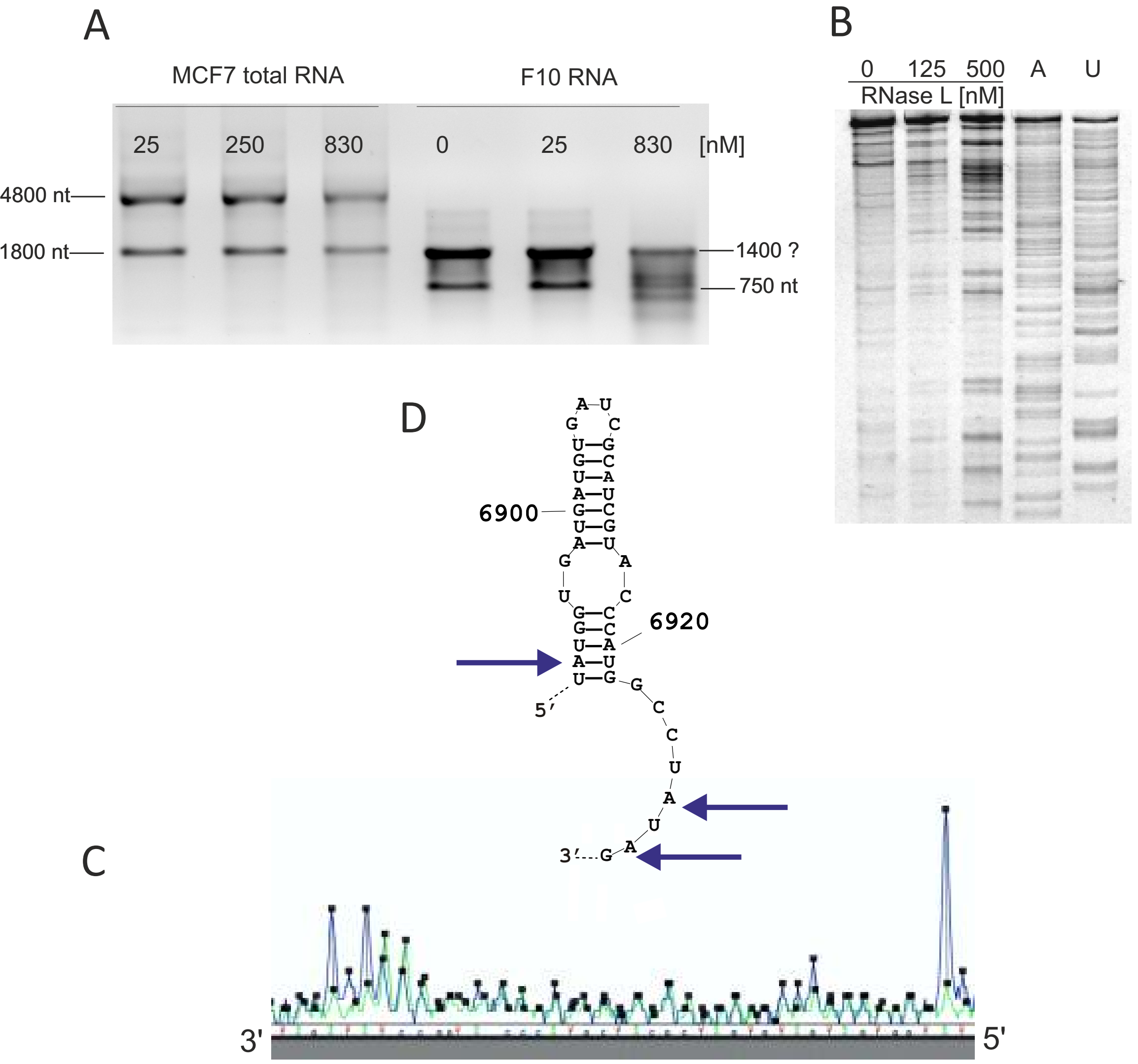

Figure SM3. RNase L assay. The viral RNA (F10 fragment of CVB3) or cellular (MCF7) total RNA was subjected to increasing amounts of RNase L: 0,25 nM, and 830 nM. Reaction products were electrophoretically separated on agarose gel. B-C).

(A) 750-nt-long 3'-terminal fragment of the CVB3 genome was digested with RNAse L at the given concentrations: 0, 125 nM, and 500 nM. Reaction products were separated on a 12% PAA gel (B) or in the capillaries (C) in parallel to sequencing lines.

Blue peaks - RNase L cleavages; green peaks - control reaction.

D) RNase L cleavage sites are displayed on a secondary structure model of the viral RNA fragment, the SL6891-6922 motif, as blue arrows.
