# Supplementary material for: New RNA Structural Elements Identified in the Coding Region of the Coxsackie B3 Virus Genome
Source: Viruses. 2020 Oct 30;12(11):1232. doi: 10.3390/v12111232 (PMC7692623; doi:10.3390/v12111232)
Supplement: Supplementary file 1 [file viruses-12-01232-s001.zip › SM4.pdf]

SM4 Table. Potential interactions with proteins of the newly identified RNA motifs in CVB3 genome fragments. Content based on data collected in the *ATtRACT* database (A datTabase of RNA binding proteins and AssoCiated moTifs, https://attract.cnic.es/index); mode - sequence search.

apical part - marked in red

>SL665-748 CCCUUUGUUGGGUUUAUACCACUUAGCUUGAAAGAGGUUAAAACAUUACAAUUCAUUGUUAAGUUGAAUACAGCAAAAUGGG

and SL652-752 GAGCUAUUAUAUAUCCCUUUGUUGGGUUUAUACCACUUAGCUUGAAAGAGGUUAAAACAUUACAAUUCAUUGUUAAGUUGAAUACAGCAAAAUGGGAGCUC

Gene name	Gene id	Mutated*	Organism	Motif	Length	Reference	Domain	Quality_score				Experiments
CELF1	ENSG00000149187	no	Homo_sapiens	UGUU	4	20947024	RRM	70;19	1.835	2.608	1.556	X-RAY DIFFRACTION
CELF1	ENSG00000149187	no	Homo_sapiens	UUGUU	5	20947024	RRM	69;18	3.562	4.939	3.165	X-RAY DIFFRACTION
CELF2	ENSG00000048740	no	Homo_sapiens	UGUU	4	PubMed ID is not availa	RRM	70;19	1.835	2.608	1.556	X-RAY DIFFRACTION
CELF2	ENSG00000048740	no	Homo_sapiens	UGUUG	5	15657417	RRM	19	3.039	3.452	2.686	SELEX of 20nt random with recombinant protein, some winner sequences are verified by EMSA.
CELF2	ENSG00000048740	no	Homo_sapiens	UUGUU	5	15657417	RRM	69;18	3.562	4.939	3.165	SELEX of 20nt random with recombinant protein, some winner sequences are verified by EMSA.
CELF2	ENSG00000048740	no	Homo_sapiens	UUGUU	5	11931771	RRM	69;18	3.562	4.939	3.165	UV crosslink, western blot, immunoprecipitation in HeLa nuclear extracts.
ELAVL2	ENSG00000107105	no	Homo_sapiens	GAAA	4	8668530	RRM	43	0.78	0.557	0.622	SELEX
ELAVL2	ENSG00000107105	no	Homo_sapiens	GUUUA	5	8497264	RRM	25	17958	4.489	3.107	SELEX of 25nt random with recombinant protein.
ENOX1	ENSG00000120658	no	Homo_sapiens	AAUACAG	7	23846655	RRM	80	7.092	7.161	6.654	RNAcompete
GRSF1	ENSG00000132463	no	Homo_sapiens	UGGGA	5	17337441	RRM	92	2.439	2.685	2.404	protein affinity purification
GRSF1	ENSG00000132463	no	Homo_sapiens	UGGGU	5	17337441	RRM	22	2.962	3.741	43985	protein affinity purification
HNRNPA1	ENSG00000135486	no	Homo_sapiens	UUUAUA	6	12426391	RRM	26	4.381	6.187	3.784	RNA affinity isolation from HeLa nuclear extract using WT and mutated RNAs. Immunoblotting.
HNRNPA1	ENSG00000135486	yes	Homo_sapiens	CUUAG	5	19404736	RRM	35	3.848	4.915	3.576	Mutational analysis, RNA affinity chromatography, UV cross-linking, immunoprecipitation with HeLa nuclear extract.
HNRNPA2B1	ENSG00000122566	no	Homo_sapiens	UUUAUA	6	12426391	RRM	26	4.381	6.187	3.784	RNA affinity isolation from HeLa nuclear extract using WT and mutated RNAs. Immunoblotting.
HNRNPF	ENSG00000169813	no	Homo_sapiens	UGGGA	5	17337441	RRM;ZF_RNPHF	92	2.439	2.685	2.404	protein affinity purification
HNRNPF	ENSG00000169813	no	Homo_sapiens	UGGGU	5	17337441	RRM;ZF_RNPHF	22	2.962	3.741	43985	protein affinity purification
HNRNPF	ENSG00000169813	no	Homo_sapiens	UGGGA	5	16885237	RRM;ZF_RNPHF	92	2.439	2.685	2.404	NMR titration using recombinant protein
HNRNPF	ENSG00000169813	no	Homo_sapiens	UGGGA	5	11571276	RRM;ZF_RNPHF	92	2.439	2.685	2.404	RNA affinity binding assays, immunoblot with WT HeLa nuclear extracts or depleted by RNA affinity chromatography.
HNRNPH1	ENSG00000169045	no	Homo_sapiens	AGAGG	5	17337441	ZF_RNPHF	46	2.572	2.435	33635	RNA-affinity chromatography assays with HeLa nuclear extract and immunoblotting.
HNRNPH1	ENSG00000169045	no	Homo_sapiens	UGGGA	5	17337441	ZF_RNPHF	92	2.439	2.685	2.404	protein affinity purification
HNRNPH1	ENSG00000169045	no	Homo_sapiens	UGGGU	5	17337441	ZF_RNPHF	22	2.962	3.741	43985	protein affinity purification
HNRNPH1	ENSG00000169045	no	Homo_sapiens	UGGGA	5	20100605	ZF_RNPHF	92	2.439	2.685	2.404	GST pull-down, mass spectrometry, Immunoprecipitation, western blotting, RNA pull-down assay, REMSA, RNA interference
HNRNPH1	ENSG00000169045	no	Homo_sapiens	UGGGA	5	11571276	ZF_RNPHF	92	2.439	2.685	2.404	RNA affinity binding assays, immunoblot with WT HeLa nuclear extracts or depleted by RNA affinity chromatography.
HNRNPH1	ENSG00000169045	no	Homo_sapiens	UGGGU	5	20100605	ZF_RNPHF	22	2.962	3.741	43985	GST pull-down, mass spectrometry, Immunoprecipitation, western blotting, RNA pull-down assay, REMSA, RNA interference
HNRNPH1	ENSG00000169045	no	Homo_sapiens	UGGGU	5	18806275	ZF_RNPHF	22	2.962	3.741	43985	Western blotting using HEK293T nuclear extract, immunodepletion, surface plasmon resonance
HNRNPH1	ENSG00000169045	yes	Homo_sapiens	UGGGA	5	12732620	ZF_RNPHF	92	2.439	2.685	2.404	UV cross-linking, pull-down assay, immunoblotting in HeLa nuclear extracts with wt and mutated RNAs.
HNRNPH2	ENSG00000126945	no	Homo_sapiens	AGAGG	5	17337441	ZF_RNPHF	46	2.572	2.435	33635	RNA-affinity chromatography assays with HeLa nuclear extract and immunoblotting.
HNRNPH2	ENSG00000126945	no	Homo_sapiens	UGGGA	5	17337441	ZF_RNPHF	92	2.439	2.685	2.404	protein affinity purification
HNRNPH2	ENSG00000126945	no	Homo_sapiens	UGGGU	5	17337441	ZF_RNPHF	22	2.962	3.741	43985	protein affinity purification
HNRNPH2	ENSG00000126945	no	Homo_sapiens	UGGGA	5	11571276	ZF_RNPHF	92	2.439	2.685	2.404	RNA affinity binding assays, immunoblot with WT HeLa nuclear extracts or depleted by RNA affinity chromatography.
HNRNPH2	ENSG00000126945	no	Homo_sapiens	UGGGU	5	18806275	ZF_RNPHF	22	2.962	3.741	43985	Western blotting using HEK293T nuclear extract, immunodepletion, surface plasmon resonance
HNRNPH2	ENSG00000126945	yes	Homo_sapiens	UGGGA	5	12732620	ZF_RNPHF	92	2.439	2.685	2.404	UV cross-linking, pull-down assay, immunoblotting in HeLa nuclear extracts with wt and mutated RNAs.
HNRNPH3	ENSG00000096746	no	Homo_sapiens	UGGGA	5	17337441	RRM	92	2.439	2.685	2.404	protein affinity purification
HNRNPH3	ENSG00000096746	no	Homo_sapiens	UGGGU	5	17337441	RRM	22	2.962	3.741	43985	protein affinity purification
HNRNPH3	ENSG00000096746	no	Homo_sapiens	UGGGA	5	11571276	RRM	92	2.439	2.685	2.404	RNA affinity binding assays, immunoblot with WT HeLa nuclear extracts or depleted by RNA affinity chromatography.
HNRNPH3	ENSG00000096746	no	Homo_sapiens	UGGGU	5	18806275	RRM	22	2.962	3.741	43985	Western blotting using HEK293T nuclear extract, immunodepletion, surface plasmon resonance
HNRNPH3	ENSG00000096746	yes	Homo_sapiens	UGGGA	5	12732620	RRM	92	2.439	2.685	2.404	UV cross-linking, pull-down assay, immunoblotting in HeLa nuclear extracts with wt and mutated RNAs.
HNRNPK	ENSG00000165119	no	Homo_sapiens	UCCCUU	6	11278705	KH	13	4.583	5.662	4.713	SELEX of 50nt random with recombinant protein. Consensus motif: u/aCCCCCu/a. Winners confirmed by EMSA with recombinant protein.
HNRNPL	ENSG00000104824	no	Homo_sapiens	AACA	4	15889141	RRM	55	1.267	1.042	1.004	SELEX of 20nt random with recombinant protein. Consensus motif: ACACACAC. Pull-down assays, UV crosslink, immunoprecipitation with HeLa nuclear extracts.
HNRNPL	ENSG00000104824	no	Homo_sapiens	ACAA	4	15889141	RRM	61	1.478	1.087	1.199	Pull-down assays, UV crosslink, immunoprecipitation with HeLa nuclear extracts.

HNRNPL	ENSG00000104824	no	Homo_sapiens	ACAU	4	15889141	RRM	56	1.405	1.431	43983	SELEX of 20nt random with recombinant protein. Consensus motif: ACACACAC. Pull-down assays, UV crosslink, immunoprecipitation with HeLa nuclear extracts.
HNRNPL	ENSG00000104824	no	Homo_sapiens	CCAC	4	15889141	RRM	32	1.142	0.892	1.398	SELEX of 20nt random with recombinant protein. Consensus motif: ACACACAC. Pull-down assays, UV crosslink, immunoprecipitation with HeLa nuclear extracts.
HNRNPL	ENSG00000104824	no	Homo_sapiens	UACA	4	15889141	RRM	60;82	2.622	2.572	2.225	SELEX of 20nt random with recombinant protein. Consensus motif: ACACACAC. Pull-down assays, UV crosslink, immunoprecipitation with HeLa nuclear extracts.
IGF2BP2	ENSG00000073792	no	Homo_sapiens	GAAUACA	7	23846655	KH;RRM	79	19906	7.163	7.177	RNAcompete
IGF2BP2	ENSG00000073792	no	Homo_sapiens	CAAUUCA	7	23846655	KH;RRM	62	7.745	7.666	7.249	RNAcompete
IGF2BP3	ENSG00000136231	no	Homo_sapiens	CAUU	4	20371350	KH;RRM	57;67	2.138	2.552	28491	PAR-clip
KHDRBS1	ENSG00000121774	no	Homo_sapiens	CAAAAU	6	9341174	KH	87	4.731	4.913	4.193	SELEX of 40nt random with recombinant protein. Winners and mutants confirmed by EMSA with recombinant protein.
KHDRBS1	ENSG00000121774	no	Homo_sapiens	UUAAAA	6	12490714	KH	51	3.655	5.356	3.163	Differential display and cDNA-representational difference analysis (cDNA-RDA) in vitro. In vivo coimmunoprecipitation. Further in vitro binding: protein incubated with labeled RNA.
KHDRBS1	ENSG00000121774	no	Homo_sapiens	UUAAAA	6	9341174	KH	51	3.655	5.356	3.163	SELEX of 40nt random with recombinant protein. Winners and mutants confirmed by EMSA with recombinant protein.
KHSRP	ENSG00000088247	no	Homo_sapiens	GGGU	4	18684992	KH	23	1.339	2.109	1.668	NMR
KHSRP	ENSG00000088247	no	Homo_sapiens	UAUUAU	6	18684992	KH	4	4.804	6.251	4.069	NMR
KHSRP	ENSG00000088247	no	Homo_sapiens	UAUUAU	6	17437720	KH	4	4.804	6.251	4.069	NMR and CD with recombinant protein
KHSRP	ENSG00000088247	no	Homo_sapiens	UAUUAU	6	18684992	KH	4	4.804	6.251	4.069	NMR, scaffold-independent analysis (SIA) with recombinant protein
KHSRP	ENSG00000088247	no	Homo_sapiens	UGGG	4	18684992	KH	92;22	1.525	1.891	1.835	NMR
KHSRP	ENSG00000088247	no	Homo_sapiens	UGGGU	5	18684992	KH	22	2.962	3.741	43985	NMR
KHSRP	ENSG00000088247	no	Homo_sapiens	UGGGU	5	18684992	KH	22	2.962	3.741	43985	NMR, scaffold-independent analysis (SIA) with recombinant protein
KHSRP	ENSG00000088247	no	Homo_sapiens	UUAG	4	18684992	KH	36	1.735	2.943	45658	NMR
KHSRP	ENSG00000088247	no	Homo_sapiens	UUGGG	5	18684992	KH	21	2.895	3.605	2.914	NMR, scaffold-independent analysis (SIA) with recombinant protein
NOVA1	ENSG00000139910	no	Homo_sapiens	UCAU	4	10811881	KH	66	1.251	1.352	0.98	SELEX
NOVA1	ENSG00000139910	no	Homo_sapiens	UUCAU	5	17655233	KH	65	2.841	3.256	2.476	NMR titrations using recombinant protein
NOVA1	ENSG00000139910	no	Homo_sapiens	CCAC	4	17655233	KH	32	1.142	0.892	1.398	NMR
NOVA1	ENSG00000139910	no	Homo_sapiens	UCAU	4	17655233	KH	66	1.251	1.352	0.98	NMR
NOVA2	ENSG00000104967	no	Homo_sapiens	UCAU	4	10811881	KH	66	1.251	1.352	0.98	SELEX
OAS1	ENSG00000089127	no	Homo_sapiens	UUGA	4	23319625	OAS1_C;NTP_TRANSF_2	41;77	2.225	2.437	1.882	X-RAY DIFFRACTION
PCBP2	ENSG00000197111	no	Homo_sapiens	CCCU	4	17526645	KH	14	0.722	0.899	1.356	X-RAY DIFFRACTION
PIWIL1	ENSG00000125207	no	Homo_sapiens	GCUU	4	21193640	GAGE;PAZ;PIWI	39	1.341	1.545	1.365	X-RAY DIFFRACTION
PPIE	ENSG00000084072	no	Homo_sapiens	UUAAAA	6	18258190	RRM;PRO_ISOMERASE	51	3.655	5.356	3.163	single RNA affinity purification
PPIE	ENSG00000084072	no	Homo_sapiens	UAUAUA	6	18258190	RRM;PRO_ISOMERASE	7	4.573	6.994	3.548	single RNA affinity purification
PPIE	ENSG00000084072	no	Homo_sapiens	AUUAUA	6	18258190	RRM;PRO_ISOMERASE	5	5.038	6.391	4.314	single RNA affinity purification
PPIE	ENSG00000084072	no	Homo_sapiens	UUUAUA	6	18258190	RRM;PRO_ISOMERASE	26	4.381	6.187	3.784	single RNA affinity purification
PPIE	ENSG00000084072	no	Homo_sapiens	AUAUAU	6	18258190	RRM;PRO_ISOMERASE	8	4.393	6.407	3.424	single RNA affinity purification
PPIE	ENSG00000084072	no	Homo_sapiens	UUAUAU	6	18258190	RRM;PRO_ISOMERASE	6	4.698	12571	4.059	single RNA affinity purification
PPIE	ENSG00000084072	no	Homo_sapiens	UAUUAU	6	18258190	RRM;PRO_ISOMERASE	4	4.804	6.251	4.069	single RNA affinity purification
PTBP1	ENSG00000011304	no	Homo_sapiens	CUUA	4	16179478	RRM	35	1.721	2.436	1.409	NMR
PTBP1	ENSG00000011304	no	Homo_sapiens	CUAU	4	16179478	RRM	3	1.998	2.095	1.615	NMR
PTBP1	ENSG00000011304	no	Homo_sapiens	CUUU	4	16179478	RRM	16	0.583	1.353	0.37	NMR
PTBP1	ENSG00000011304	no	Homo_sapiens	CUUG	4	16179478	RRM	40	1.189	1.561	1.112	NMR
PTBP1	ENSG00000011304	no	Homo_sapiens	CCUUU	5	16179478	RRM	15	2.633	3.314	2.597	NMR
RBMS3	ENSG00000144642	no	Homo_sapiens	UAUAUA	6	23846655	RRM	7	4.573	6.994	3.548	RNAcompete
RBMS3	ENSG00000144642	no	Homo_sapiens	AUAUAUC	7	23846655	RRM	8	7.769	8.734	6.968	RNAcompete
RBMX	ENSG00000147274	no	Homo_sapiens	AAAAU	5	24692659	RRM	88	2.073	2.713	1.518	NMR spectroscopy, isothermal titration calorimetry. EMSA with recombinat protein and other competitor protein (HTra2beta1).
RNASEL	ENSG00000135828	no	Homo_sapiens	AAAA	4	24578532	ANK;PKINASE;RIBONUC_2	53;88	1.157	1.589	0.611	X-RAY DIFFRACTION
SRP19	ENSG00000153037	no	Homo_sapiens	GAGC	4	11641499	SRP19	0;95	2.205	1.766	2.713	X-RAY DIFFRACTION
SRP19	ENSG00000153037	no	Homo_sapiens	GGAGC	5	20179341	SRP19	94	2.762	2.141	27454	X-RAY DIFFRACTION
SRP54	ENSG00000100883	no	Homo_sapiens	AGGU	4	12244299	SRP54,SRP_SPB	48	41640	1.596	1.321	X-RAY DIFFRACTION
SRP54	ENSG00000100883	no	Homo_sapiens	GGAGC	5	12244299	SRP54,SRP_SPB	94	2.762	2.141	27454	X-RAY DIFFRACTION
SRP68	ENSG00000167881	no	Homo_sapiens	GGAGC	5	24700861	SRP68	94	2.762	2.141	27454	X-RAY DIFFRACTION
SRP68	ENSG00000167881	no	Homo_sapiens	UAAG	4	24700861	SRP68	73	1.696	2.614	1.484	X-RAY DIFFRACTION
SRSF10	ENSG00000188529	no	Homo_sapiens	AAAGAGG	7	23846655	RRM	44	6.483	6.129	6.504	RNAcompete
SRSF5	ENSG00000100650	no	Homo_sapiens	ACAGC	5	9649504	RRM	83	3.139	2.494	3.589	Functional SELEX of constructs with 20nt random by splicing in HeLa S100 extracts complemented by recombinant protein. Consensus motif: ACDGS. UV crosslink, competition and immunoprecipitation assays in HeLa nuclear extracts.
SRSF5	ENSG00000100650	no	Homo_sapiens	AGAGG	5	9649504	RRM	46	2.572	2.435	33635	Functional SELEX of constructs with 20nt random by splicing in HeLa S100 extracts complemented by recombinant protein. Consensus motif: ACDGS. UV crosslink, competition and immunoprecipitation assays in HeLa nuclear extracts.

SRSF9	ENSG00000111786	no	Homo_sapiens	ACCAC	5	17548433	RRM	31	3.514	36192	3.459	SELEX of 20nt random with recombinant protein. Consensus motif: AGGAC. EMSA, UV crosslink, SDS-PAGE with HeLa nuclear extracts.
TIA1	ENSG00000116001	no	Homo_sapiens	AUUC	4	8576255	RRM	64	1.525	1.747	1.194	SELEX of 68nt random with recombinant protein. Winners confirmed by UV cross-linking and competition assay.
TIA1	ENSG00000116001	no	Homo_sapiens	CUUUG	5	8576255	RRM	16	2.616	2.808	2.515	SELEX of 68nt random with recombinant protein. Winners confirmed by UV cross-linking and competition assay.
TIA1	ENSG00000116001	no	Homo_sapiens	GUUUA	5	8576255	RRM	25	17958	4.489	3.107	SELEX of 68nt random with recombinant protein. Winners confirmed by UV cross-linking and competition assay.
TIAL1	ENSG00000151923	no	Homo_sapiens	AUUA	4	8576255	RRM	58;5	2.407	3.246	1.818	SELEX of 68nt random with recombinant protein. Winners confirmed by UV cross-linking and competition assay.
TIAL1	ENSG00000151923	no	Homo_sapiens	AUUC	4	8576255	RRM	64	1.525	1.747	1.194	SELEX of 68nt random with recombinant protein. Winners confirmed by UV cross-linking and competition assay.
TIAL1	ENSG00000151923	no	Homo_sapiens	CUUUG	5	8576255	RRM	16	2.616	2.808	2.515	SELEX of 68nt random with recombinant protein. Winners confirmed by UV cross-linking and competition assay.
XPO5	ENSG00000124571	no	Homo_sapiens	GUUU	4	19965479	XPO1	25	0.972	1.868	0.676	X-RAY DIFFRACTION
YTHDC1	ENSG00000083896	no	Homo_sapiens	GAAUAC	6	20167602	YTH	79	6.179	5.896	5.872	SELEX
ZFP36	ENSG00000128016	no	Homo_sapiens	CCCUUU	6	18367721	ZF_CCCH	14	4.658	5.536	4.774	RIP-chip
ZRANB2	ENSG00000132485	no	Homo_sapiens	AGGUUA	6	19304800	ZF_RANBP	48	5.927	6.616	5.613	SELEX of 25nt random with recombinant protein. Consensus motifs: CAGGUAA and AAGGUAA. Fluorescence anisotropy.
ZRANB2	ENSG00000132485	no	Homo_sapiens	GAGGUU	6	19304800	ZF_RANBP	47	5.295	5.765	4.913	SELEX of 25nt random with recombinant protein. Consensus motifs: CAGGUAA and AAGGUAA.
SRSF5	ENSG00000100650	no	Homo_sapiens	UACAGC	6	11967553;12824367	RRM	82	5.711	5.025	34090	SELEX
RBMX	ENSG00000147274	no	Homo_sapiens	AAGU	4	12761049	RRM	74	1.262	1.367	1.056	Mutagenesis;Deletion analysis
SRSF2	ENSG00000161547	no	Homo_sapiens	UUAG	4	10022872;9710624;762	RRM	36	1.735	2.943	45658	In vitro splicing assays and RNA competition assays
HNRNPA1	ENSG00000135486	no	Homo_sapiens	UUAG	4	10022872;9710624;762	RRM	36	1.735	2.943	45658	In vitro splicing assays and RNA competition assays
HNRNPH1	ENSG00000169045	no	Homo_sapiens	GGGA	4	11847131;9343212	ZF_RNPHF	93	0.817	1.002	1.164	snRNA inactivation in HeLa cell nuclear extract;Western blot

>SL799-854 apical loop - marked in red

UAGCGGCAAUUCCAUCAUUCACUACACAAAUAUUAAUUAUUACAAGGAUGCCGCAU

Gene name	Gene id	Mutated	Organism	Motif	Length	Reference	Domain		Quality_score			Experiments
A1CF	ENSG00000148584	no	Homo_sapiens	UUAAUUA	7	23846655	RRM	32	7.527	9.839	6.913	RNAcompete
HNRNPL	ENSG00000104824	no	Homo_sapiens	ACAA	4	15889141	RRM	25;41	3.365	2.974	3.086	Pull-down assays, UV crosslink, immunoprecipitation with HeLa nuclear extracts.
HNRNPL	ENSG00000104824	no	Homo_sapiens	ACAC	4	15889141	RRM	23	2.584	2.448	2.497	SELEX of 20nt random with recombinant protein. Pull-down assays, UV crosslink, immunoprecipitation with HeLa nuclear extracts.
HNRNPL	ENSG00000104824	no	Homo_sapiens	ACACAAA	7	23846655	RRM	23	7.892	8.107	7.357	RNAcompete
HNRNPL	ENSG00000104824	no	Homo_sapiens	ACACAAA	7	23846655	RRM	23	7.892	8.107	7.357	RNAcompete
HNRNPL	ENSG00000104824	no	Homo_sapiens	CACA	4	15889141	RRM	24	2.043	1.976	1.966	SELEX of 20nt random with recombinant protein. Consensus motif: ACACACAC. Pull-down assays, UV crosslink, immunoprecipitation with HeLa nuclear extracts.
HNRNPL	ENSG00000104824	no	Homo_sapiens	UACA	4	15889141	RRM	40;22	3.508	3.459	3.111	SELEX of 20nt random with recombinant protein. Consensus motif: ACACACAC. Pull-down assays, UV crosslink, immunoprecipitation with HeLa nuclear extracts.
IGF2BP3	ENSG00000136231	no	Homo_sapiens	CAUU	4	20371350	KH;RRM	15	2.025	2.439	1.666	PAR-clip
MBNL1	ENSG00000152601	no	Homo_sapiens	AUGCCG	6	20071745	ZF_CCCH	47	43898	7.305	9.228	SELEX of 32nt random with recombinant protein. Consensus motifs: YGCY, YYYGCUKY.
MBNL1	ENSG00000152601	no	Homo_sapiens	UGCC	4	20071745	ZF_CCCH	48	1.875	1.732	2.133	SELEX
NOVA1	ENSG00000139910	no	Homo_sapiens	UCAC	4	10811881	KH	18	2.351	2.349	2.223	SELEX
NOVA1	ENSG00000139910	no	Homo_sapiens	UCAU	4	10811881	KH	14	2.138	2.238	1.867	SELEX
NOVA1	ENSG00000139910	no	Homo_sapiens	UCAC	4	17655233	KH	18	2.351	2.349	2.223	NMR
NOVA1	ENSG00000139910	no	Homo_sapiens	CCAU	4	17655233	KH	11	2.242	2.114	2.141	NMR
NOVA1	ENSG00000139910	no	Homo_sapiens	UCAU	4	17655233	KH	14	2.138	2.238	1.867	NMR
NOVA2	ENSG00000104967	no	Homo_sapiens	UCAC	4	10811881	KH	18	2.351	2.349	2.223	SELEX
NOVA2	ENSG00000104967	no	Homo_sapiens	UCAU	4	10811881	KH	14	2.138	2.238	1.867	SELEX
PABPC1	ENSG00000070756	no	Homo_sapiens	ACAAAUA	7	7908267	RRM	25	7.782	8.322	7.113	SELEX
PPIE	ENSG00000084072	no	Homo_sapiens	UAUUAA	6	18258190	RRM;PRO_ISOMERASE	30	5.783	7.219	5.268	single RNA affinity purification
PPIE	ENSG00000084072	no	Homo_sapiens	UAAUUA	6	18258190	RRM;PRO_ISOMERASE	33	5.954	8.047	5.371	single RNA affinity purification
PPIE	ENSG00000084072	no	Homo_sapiens	AUAUUA	6	18258190	RRM;PRO_ISOMERASE	29	5.952	7.157	5.304	single RNA affinity purification
PPIE	ENSG00000084072	no	Homo_sapiens	UUAUUA	6	18258190	RRM;PRO_ISOMERASE	36	5.579	42552	4.928	single RNA affinity purification
PPIE	ENSG00000084072	no	Homo_sapiens	AUUAAU	6	18258190	RRM;PRO_ISOMERASE	31	5.882	7.247	5.324	single RNA affinity purification
PPIE	ENSG00000084072	no	Homo_sapiens	AAAUAU	6	18258190	RRM;PRO_ISOMERASE	27	5.039	43988	4.448	single RNA affinity purification
PPIE	ENSG00000084072	no	Homo_sapiens	AAUUAU	6	18258190	RRM;PRO_ISOMERASE	34	5.589	6.705	5.052	single RNA affinity purification
PPIE	ENSG00000084072	no	Homo_sapiens	UUAAUU	6	18258190	RRM;PRO_ISOMERASE	32	5.348	21002	28946	single RNA affinity purification
PPIE	ENSG00000084072	no	Homo_sapiens	AAUAUU	6	18258190	RRM;PRO_ISOMERASE	28	5.215	6.409	4.659	single RNA affinity purification
PPIE	ENSG00000084072	no	Homo_sapiens	AUUAUU	6	18258190	RRM;PRO_ISOMERASE	35	5.283	6.675	24929	single RNA affinity purification
PTBP1	ENSG00000011304	no	Homo_sapiens	CUAC	4	16179478	RRM	21	2.958	2.399	2.947	NMR
RBMY1A1	ENSG00000234414	no	Homo_sapiens	CACAA	5	17318228	RRM	24	4.518	4.194	4.284	SELEX

SRSF3	ENSG00000112081	no	Homo_sapiens	CACUACAC	8	10094314	RRM	19	11.152	28764	10.582	SELEX of 20-nt random with recombinant protein. Consensus motifs: AGACKACGAY, ACGAGAGAY, WCAUCGAYY, YWCUUCAU and CUWCAAC. Winners confirmed by EMSA with recombinant protein, UV crosslink, complementation assay, immunoprecipitations with HeLa S100 and nuclear extracts.
SRSF3	ENSG00000112081	no	Homo_sapiens	CAUC	4	17036044	RRM	12	2.322	1.776	2.484	NMR spectroscopy
SRSF3	ENSG00000112081	no	Homo_sapiens	CAUCAU	6	17036044	RRM	12	6.084	16193	5.978	NMR spectroscopy
SRSF3	ENSG00000112081	no	Homo_sapiens	UUCAC	5	17036044	RRM	17	4.276	4.354	4.015	NMR spectroscopy
SRSF9	ENSG00000111786	no	Homo_sapiens	AGGAU	5	17548433	RRM	44	4.172	3.932	4.083	SELEX of 20nt random with recombinant protein. Consensus motif: AGGAC. EMSA, UV crosslink, SDS-PAGE with HeLa nuclear extracts.
SRSF9	ENSG00000111786	no	Homo_sapiens	AGGAU	5	17548433	RRM	44	4.172	3.932	4.083	SELEX
SRSF9	ENSG00000111786	no	Homo_sapiens	GGAUGCC	7	23846655	RRM	45	8.123	7.359	8.919	RNAcompete
TIA1	ENSG00000116001	no	Homo_sapiens	AUUC	4	8576255	RRM	16;8	3.412	3.634	3.081	SELEX of 68nt random with recombinant protein. Winners confirmed by UV cross-linking and competition assay.
TIAL1	ENSG00000151923	no	Homo_sapiens	AUUA	4	8576255	RRM	31;38;3	3.879	4.717	47178	SELEX of 68nt random with recombinant protein. Winners confirmed by UV cross-linking and competition assay.
TIAL1	ENSG00000151923	no	Homo_sapiens	AUUC	4	8576255	RRM	16;8	3.412	3.634	3.081	SELEX of 68nt random with recombinant protein. Winners confirmed by UV cross-linking and competition assay.
XPO5	ENSG00000124571	no	Homo_sapiens	CCGC	4	19965479	XPO1	50	3.081	2.616	4.656	X-RAY DIFFRACTION
YBX1	ENSG00000065978	no	Homo_sapiens	CAUC	4	22730292	CSD	12	2.322	1.776	2.484	In vivo splicing in HEK-293 cells with wt and mutated sequences, EMSA
YBX1	ENSG00000065978	no	Homo_sapiens	CAUC	4	22730292	CSD	12	2.322	1.776	2.484	SELEX of 20nt random sequences with recombinant protein. Winners confirmed by EMSA with recombinant protein.
YTHDC1	ENSG00000083896	no	Homo_sapiens	GGAUGC	6	20167602	YTH	45	6.404	5.832	6.954	SELEX
NOVA1	ENSG00000139910	no	Homo_sapiens	UCAC	4	15367696	KH	18	2.351	2.349	2.223	RNA affinity binding assays

>SL2313-2343 apical loop - marked in red

GCUGGUAUCAAACAAACAUAGUGGUCCCAGC

Gene name	Gene id	Mutated	Organism	Motif	Length	Reference		Domain	Quality_score			Experiments
HNRNPL	ENSG00000104824	no	Homo_sapiens	AACA	4	15889141	RRM	10;14	4.074	31107	3.812	SELEX of 20nt random with recombinant protein. Consensus motif: ACACACAC. Pull-down assays UV crosslink immunoprecipitation with HeLa nuclear extracts.
HNRNPL	ENSG00000104824	no	Homo_sapiens	ACAA	4	15889141	RRM	11	3.286	2.894	3.007	Pull-down assays UV crosslink immunoprecipitation with HeLa nuclear extracts.
HNRNPL	ENSG00000104824	no	Homo_sapiens	ACAU	4	15889141	RRM	15	3.212	3.239	2.867	SELEX of 20nt random with recombinant protein. Consensus motif: ACACACAC. Pull-down assays UV crosslink immunoprecipitation with HeLa nuclear extracts.
HNRNPL	ENSG00000104824	no	Homo_sapiens	AAACAAA	7	23846655	RRM	9	7.575	8.176	44080	RNAcompete
HNRNPLL	ENSG00000143889	no	Homo_sapiens	ACAAACA	7	23846655	RRM	11	8.762	8.922	8.162	RNAcompete
IGF2BP2	ENSG00000073792	no	Homo_sapiens	ACAAACA	7	23846655	KH;RRM	11	8.762	8.922	8.162	RNAcompete
IGF2BP3	ENSG00000136231	no	Homo_sapiens	ACAAACA	7	23846655	KH;RRM	11	8.762	8.922	8.162	RNAcompete
NOVA1	ENSG00000139910	no	Homo_sapiens	CCAG	4	17655233	KH	26	2.306	1.995	24504	NMR
PABPC1	ENSG00000070756	no	Homo_sapiens	ACAAACA	7	7908267	RRM	11	8.762	8.922	8.162	SELEX
PTBP1	ENSG00000011304	no	Homo_sapiens	CUGG	4	16179478	RRM	1	2.221	1.981	2.591	NMR
RBMX	ENSG00000147274	no	Homo_sapiens	AUCAAA	6	24692659	RRM	6	7.191	6.841	6.796	NMR spectroscopy isothermal titration calorimetry
RBMX	ENSG00000147274	no	Homo_sapiens	UCAAA	5	24692659	RRM	7	35156	4.959	43955	SOLUTION NMR
SAMD4A	ENSG00000020577	no	Homo_sapiens	GCUGGUA	7	23846655	SAM	0	9.654	9.681	9.788	RNAcompete
SRP14	ENSG00000140319	no	Homo_sapiens	GUGG	4	11089964	SRP14	20	2.639	2.503	3.0	X-RAY DIFFRACTION
ZNF346	ENSG00000113761	no	Homo_sapiens	GUGGU	5	24521053	ZF_C2H2	20	4.999	33329	5.001	SOLUTION NMR

>SL2562-2590 apical loop - marked in red

GUCACACGUCACAAGUAGUGCCGGGUGAC;

Gene name	Gene id	Mutated	Organism	Motif	Length	Reference		Domain	Quality_score			Experiments
FUS	ENSG00000089280	no	Homo_sapiens	GGGUGA	6	11098054	RRM	22	6.831	7.462	7.302;	SELEX of 25nt random with recombinant protein. Consensus motif: GGUG. SDS-PAGE; EMSA; UV crosslink; competition and immunoprecipitation assays with HeLa nuclear extracts.
FUS	ENSG00000089280	no	Homo_sapiens	GGUG	4	11098054	ZF_RANBP;RRM	23	2.813	2.917	3.206	SELEX
HNRNPL	ENSG00000104824	no	Homo_sapiens	ACAA	4	15889141	RRM	10	3.393	3.001	3.113;	Pull-down assays; UV crosslink;immunoprecipitation with HeLa nuclear extracts.
HNRNPL	ENSG00000104824	no	Homo_sapiens	ACAC	4	15889141	RRM	3	3.612	3.476	3.524;	SELEX of 20nt random with recombinant protein. Pull-down assays; UV crosslink;immunoprecipitation with HeLa nuclear extracts.
HNRNPL	ENSG00000104824	no	Homo_sapiens	CACA	4	15889141	RRM	9;2	44016	4.004	3.994	SELEX of 20nt random with recombinant protein. Consensus motif: ACACACAC. Pull-down assays; UV crosslink;immunoprecipitation with HeLa nuclear extracts.
HNRNPL	ENSG00000104824	no	Homo_sapiens	CACG	4	15889141	RRM	4	4.768	4.209	5.357;	SELEX of 20nt random with recombinant protein. Consensus motif: ACACACAC. Pull-down assays; UV crosslink;immunoprecipitation with HeLa nuclear extracts.
KHSRP	ENSG00000088247	no	Homo_sapiens	GGGU	4	18684992	KH	22	3.253	4.023	3.582;	NMR
MBNL1	ENSG00000152601	no	Homo_sapiens	UGCC	4	20071745	ZF_CCCH	18	2.902	2.759	3.161;	SELEX
NOVA1	ENSG00000139910	no	Homo_sapiens	UCAC	4	10811881	KH	8;1	4.379	4.376	4.251	SELEX
NOVA1	ENSG00000139910	no	Homo_sapiens	UCAC	4	17655233	KH	8;1	4.379	4.376	4.251	NMR
NOVA2	ENSG00000104967	no	Homo_sapiens	UCAC	4	10811881	KH	8;1	4.379	4.376	4.251	SELEX

RBMY1A1	ENSG00000234414	no	Homo_sapiens	CACAA	5	17318228	RRM	9	5.575	45778	5.341;	SELEX
SRSF2	ENSG00000161547	no	Homo_sapiens	AGUAG	5	11847131	RRM	13	5.715	6.569	5.099;	RNA affinity chromatography assay and immunoblot.
SRSF5	ENSG00000100650	no	Homo_sapiens	ACACG	5	9649504	RRM	3	7.165	6.565	7.679;	Functional SELEX of constructs with 20nt random by splicing in HeLa S100 extracts complemented by recombinant protein. Consensus motif: ACDGS. UV crosslink; competition and immunoprecipitation assays in HeLa nuclear extracts.
YTHDC1	ENSG00000083896	no	Homo_sapiens	UAGUGC	6	20167602	YTH	15	17746	9.049	8.182;	SELEX
ZRANB2	ENSG00000132485	no	Homo_sapiens	GGGUGA	6	19304800	ZF_RANBP	22	6.831	7.462	7.302;	SELEX of 25nt random with recombinant protein. Consensus motifs: CAGGUAA and AAGGUAA.
SRSF5	ENSG00000100650	no	Homo_sapiens	CACGUC	6	11967553;12824367	RRM	4	9.099	20302	9.831	SELEX
RBMX	ENSG00000147274	no	Homo_sapiens	AAGU	4	12761049	RRM	12	3.176	3.281	2.971	Mutagenesis;Deletion analysis
SRSF6	ENSG00000124193	no	Homo_sapiens	UCACACG	7	11967553;12824367	RRM	1	11.358	11.035	11.791	SELEX
SRSF1	ENSG00000136450	no	Homo_sapiens	CACACGU	7	11967553;12824367	RRM	2	10.857	11.047	10.942	SELEX
NOVA1	ENSG00000139910	no	Homo_sapiens	UCAC	4	15367696	KH	8;1	4.379	4.376	4.251	RNA affinity binding assays

>SL2664-2719 apical loop - marked in red

UCAGCAUGCGUGUACUUUACGGAGUAUGAAAACUCAGGUGCCAAGCGGUAUGCUGA

Gene name	Gene id	Mutated	Organism	Motif	Length	Reference	Domain		Quality_score			Experiments
CELF2	ENSG00000048740	no	Homo_sapiens	CGUGU	5	15657417	RRM	8	5.648	5.374	6.178	SELEX of 20nt random with recombinant protein; some winner sequences are verified by EMSA.
CPEB4	ENSG00000113742	no	Homo_sapiens	CUUUA	5	24990967	RRM	14	4.029	4.889	3.656	SOLUTION NMR
ELAVL2	ENSG00000107105	no	Homo_sapiens	CUUUA	5	8497264	RRM	14	4.029	4.889	3.656	SELEX of 25nt random with recombinant protein.
ELAVL2	ENSG00000107105	no	Homo_sapiens	GAAA	4	8668530	RRM	27	1.667	1.443	1.508	SELEX
FUS	ENSG00000089280	no	Homo_sapiens	GGUG	4	11098054	ZF_RANBP;RRM	36	1.786	1.889	2.179	SELEX
IGF2BP2	ENSG00000073792	no	Homo_sapiens	AAACUCA	7	23846655	KH;RRM	29	7.934	7.737	7.697	RNAcompete
IGF2BP3	ENSG00000136231	no	Homo_sapiens	AAACUCA	7	23846655	KH;RRM	29	7.934	7.737	7.697	RNAcompete
KHDRBS1	ENSG00000121774	no	Homo_sapiens	GAAAAC	6	9341174	KH	27	30803	5.427	5.723	SELEX of 40nt random with recombinant protein. Winners and mutants confirmed by EMSA with recombinant protein.
LIN28A	ENSG00000131914	no	Homo_sapiens	CGGAGUA	7	23846655	ZF_CCHC;CSD	19	10.971	10.174	11.476	RNAcompete
MBNL1	ENSG00000152601	no	Homo_sapiens	UGCC	4	20071745	ZF_CCCH	38	1.875	1.732	2.133	SELEX
MBNL1	ENSG00000152601	no	Homo_sapiens	UGCU	4	20071745	ZF_CCCH	50	1.774	1.671	1.879	SELEX
NOVA1	ENSG00000139910	no	Homo_sapiens	UCAG	4	17655233	KH	0;33	2.818	44014	2.854	NMR
NUDT21	ENSG00000167005	no	Homo_sapiens	UGUA	4	21295486	NUDIX_2	10	2.321	3.063	1.894	X-RAY DIFFRACTION
PTBP1	ENSG00000011304	no	Homo_sapiens	CUCA	4	16179478	RRM	32	1.885	1.861	1.817	NMR
PTBP1	ENSG00000011304	no	Homo_sapiens	CUGA	4	16179478	RRM	52	1.794	1.627	1.921	NMR
PTBP1	ENSG00000011304	no	Homo_sapiens	CUUU	4	16179478	RRM	14	17168	45323	1.257	NMR
RBFOX1	ENSG00000078328	no	Homo_sapiens	AGCAUG	6	22434879	RRM	2	41791	5.937	6.083	Mutational analysis. In vivo splicing with wt and mutated sequences.
RBFOX1	ENSG00000078328	no	Homo_sapiens	AGCAUG	6	16537540	RRM	2	41791	5.937	6.083	SELEX of 40nt random with recombinant protein. Consensus motif: UGCAUG. Immunoblot; siRNA; pulldown assay.
RBFOX1	ENSG00000078328	no	Homo_sapiens	AGCAUGC	7	23846655	RRM	2	8.587	8.209	8.701	RNAcompete
RNASEL	ENSG00000135828	no	Homo_sapiens	AAAA	4	24578532	ANK;PKINASE;RIBONUC_2	28	1.043	1.475	0.498	X-RAY DIFFRACTION
SRP54	ENSG00000100883	no	Homo_sapiens	AGGU	4	12244299	SRP54;SRP_SPB	35	2.026	2.483	2.208	X-RAY DIFFRACTION
SRP68	ENSG00000167881	no	Homo_sapiens	UGCUG	5	24700861	SRP68	50	3.209	2.713	3.544	X-RAY DIFFRACTION
SRSF1	ENSG00000136450	no	Homo_sapiens	CUCAGGU	7	9649504	RRM	32	7.358	8.158	7.405	Functional SELEX of constructs with 20nt random by splicing in HeLa S100 extracts complemented by recombinant protein. Consensus motif: SRSASGA. UV crosslink; competition and immunoprecipitation assays in HeLa nuclear extracts.
SRSF2	ENSG00000161547	no	Homo_sapiens	GGAGUA	6	23846655	RRM	20	6.886	6.657	6.833	RNAcompete
SRSF6	ENSG00000124193	no	Homo_sapiens	UGCGUG	6	9649504	RRM	6	47300	7.181	8.093	Functional SELEX of constructs with 20nt random by splicing in HeLa S100 extracts complemented by recombinant protein. Consensus motif: USCGKM. UV crosslink; competition and immunoprecipitation assays in HeLa nuclear extracts.
SRSF9	ENSG00000111786	no	Homo_sapiens	AUGAAAA	7	23846655	RRM	25	6.902	6.695	6.552	RNAcompete
SRSF9	ENSG00000111786	no	Homo_sapiens	CGGAG	5	17548433	RRM	19	5.038	4.375	6.252	SELEX of 20nt random with recombinant protein. Consensus motif: AGGAC. EMSA, UV crosslink, SDS-PAGE with HeLa nuclear extracts.
SSB	ENSG00000138385	no	Homo_sapiens	UGCUG	5	16387655	LA;RM	50	3.209	2.713	3.544	X-RAY DIFFRACTION
TIA1	ENSG00000116001	no	Homo_sapiens	CUUUA	5	8576255	RRM	14	4.029	4.889	3.656	SELEX of 68nt random with recombinant protein. Winners confirmed by UV cross-linking and competition assay.
TIAL1	ENSG00000151923	no	Homo_sapiens	CUUUA	5	8576255	RRM	14	4.029	4.889	3.656	SELEX of 68nt random with recombinant protein. Winners confirmed by UV cross-linking and competition assay.
YTHDC1	ENSG00000083896	no	Homo_sapiens	GCAUGC	6	20167602	YTH	3	6.688	6.608	6.623	EMSA
YTHDC1	ENSG00000083896	no	Homo_sapiens	GCAUGC	6	20167602	YTH	3	6.688	6.608	6.623	SELEX
ZRANB2	ENSG00000132485	no	Homo_sapiens	CGGUAU	6	19304800	ZF_RANBP	45	9.411	9.078	9.833	SELEX of 25nt random with recombinant protein. Consensus motifs: CAGGUAA and AAGGUAA.
SRSF5	ENSG00000100650	no	Homo_sapiens	UACGGA	6	11967553;12824367	RRM	17	8.989	8.124	9.627	SELEX

>SL4558-4656 apical loop - marked in red

GGCCGUGGUGAUUAUGGACGAUCUAUGCCAGAAUCCUGAUGGGAAAGACGUCUCCUUGUUCUGCCAAAUGGUUUCCAGUGUAGAUUUUGUACCACCCAUGG

Gene name	Gene id	Mutated	Organism	Motif	Length	Reference		Domain	Quality_score			Experiments
CELF1	ENSG00000149187	no	Homo_sapiens	UGUU	4	20947024	RRM	56	1.617	14277	1.338	X-RAY DIFFRACTION
ACO1	ENSG00000122729	no	Homo_sapiens	CAGUGU	6	8021254	ACONITASE_C	22	5.925	34090	5.912	SELEX
CELF1	ENSG00000149187	no	Homo_sapiens	UUGUU	5	20947024	RRM	55	3.354	4.731	2.958	X-RAY DIFFRACTION
CELF2	ENSG00000048740	no	Homo_sapiens	UGUU	4	PubMed ID is not available	RRM	56	1.617	14277	1.338	X-RAY DIFFRACTION
CELF2	ENSG00000048740	no	Homo_sapiens	UUGUU	5	15657417	RRM	55	3.354	4.731	2.958	SELEX of 20nt random with recombinant protein;some winner sequences are verified by EMSA.
CELF2	ENSG00000048740	no	Homo_sapiens	UUGUU	5	11931771	RRM	55	3.354	4.731	2.958	UV crosslink; western blot; immunoprecipitation in HeLa nuclear extracts.
CELF2	ENSG00000048740	no	Homo_sapiens	UGUUC	5	15657417	RRM	3	4.285	4.558	4.199	SELEX of 20nt random with recombinant protein some winner sequences are verified by EMSA.
CMTR1	ENSG00000137200	no	Homo_sapiens	GAUC	4	24402442	FTSJ;G-PATCH	19	2.884	2.436	2.799	X-RAY DIFFRACTION
ELAVL1	ENSG00000066044	no	Homo_sapiens	AUUU	4	23519412	RRM	30	1.525	2.659	1.034	X-RAY DIFFRACTION
ELAVL1	ENSG00000066044	no	Homo_sapiens	AUUUU	5	23519412	RRM	30	2.874	4.474	2.317	X-RAY DIFFRACTION
ELAVL1	ENSG00000066044	no	Homo_sapiens	UUUU	4	23519412	RRM	31	0.793	2.648	0.257	X-RAY DIFFRACTION
ELAVL2	ENSG00000107105	no	Homo_sapiens	GAAA	4	8668530	RRM	42	1.562	1.339	1.403	SELEX
ELAVL2	ENSG00000107105	no	Homo_sapiens	GUUUC	5	8497264	RRM	17	4.264	4.956	4.043	SELEX of 25nt random with recombinant protein.
ELAVL4	ENSG00000162374	no	Homo_sapiens	AUUU	4	11175903	RRM	30	1.525	2.659	1.034	X-RAY DIFFRACTION
ESRP2	ENSG00000103067	no	Homo_sapiens	UGGGAAA	7	23846655	RRM	39	33390	6.723	6.984	RNAcompete
FUS	ENSG00000089280	no	Homo_sapiens	GGUG	4	11098054	ZF_RANBP;RRM	6	1.681	1.785	2.074	SELEX
FUS	ENSG00000089280	no	Homo_sapiens	UGGUGA	6	11098054	RRM	5	5.374	5.178	5.496	SELEX of 25nt random with recombinant protein. Consensus motif: GGUG. SDS-PAGE; EMSA; UV crosslink; competition and immunoprecipitation assays with HeLa nuclear extracts.
GRSF1	ENSG00000132463	no	Homo_sapiens	UGGGA	5	17337441	RRM	39	3.232	3.478	3.196	protein affinity purification
HNRNPF	ENSG00000169813	no	Homo_sapiens	UGGGA	5	17337441	RRM;ZF_RNPHF	39	3.232	3.478	3.196	protein affinity purification
HNRNPF	ENSG00000169813	no	Homo_sapiens	UGGGA	5	16885237	RRM;ZF_RNPHF	39	3.232	3.478	3.196	NMR titration using recombinant protein
HNRNPF	ENSG00000169813	no	Homo_sapiens	UGGGA	5	11571276	RRM;ZF_RNPHF	39	3.232	3.478	3.196	immunoblot with WT HeLa nuclear extracts or depleted by RNA affinity chromatography;RNA affinity binding assays
HNRNPH1	ENSG00000169045	no	Homo_sapiens	UGGGA	5	17337441	ZF_RNPHF	39	3.232	3.478	3.196	protein affinity purification
HNRNPH1	ENSG00000169045	no	Homo_sapiens	UGGGA	5	20100605	ZF_RNPHF	39	3.232	3.478	3.196	GST pull-down; western blotting; mass spectrometry; Immunoprecipitation; RNA pull-down assay; REMSA; RNA interference
HNRNPH1	ENSG00000169045	no	Homo_sapiens	UGGGA	5	11571276	ZF_RNPHF	39	3.232	3.478	3.196	immunoblot with WT HeLa nuclear extracts or depleted by RNA affinity chromatography; RNA affinity binding assays
HNRNPH1	ENSG00000169045	yes	Homo_sapiens	UGGGA	5	12732620	ZF_RNPHF	39	3.232	3.478	3.196	UV cross-linking; immunoblotting in HeLa nuclear extracts with wt and mutated RNAs; pull-down assay
HNRNPH1	ENSG00000169045	no	Homo_sapiens	GGGA	4	11847131;9343212	ZF_RNPHF	40	1.599	1.784	1.945	snRNA inactivation in HeLa cell nuclear extract;Western blot
HNRNPH2	ENSG00000126945	no	Homo_sapiens	UGGGA	5	17337441	ZF_RNPHF	39	3.232	3.478	3.196	protein affinity purification
HNRNPH2	ENSG00000126945	no	Homo_sapiens	UGGGA	5	11571276	ZF_RNPHF	39	3.232	3.478	3.196	immunoblot with WT HeLa nuclear extracts or depleted by RNA affinity chromatography; RNA affinity binding assays
HNRNPH2	ENSG00000126945	yes	Homo_sapiens	UGGGA	5	12732620	ZF_RNPHF	39	3.232	3.478	3.196	UV cross-linking; immunoblotting in HeLa nuclear extracts with wt and mutated RNAs; pull-down assay; immunoblotting in HeLa nuclear extracts with wt and mutated RNAs.
HNRNPH3	ENSG00000096746	no	Homo_sapiens	UGGGA	5	17337441	RRM	39	3.232	3.478	3.196	protein affinity purification
HNRNPH3	ENSG00000096746	no	Homo_sapiens	UGGGA	5	11571276	RRM	39	3.232	3.478	3.196	immunoblot with WT HeLa nuclear extracts or depleted by RNA affinity chromatography.RNA affinity binding assays
HNRNPH3	ENSG00000096746	yes	Homo_sapiens	UGGGA	5	12732620	RRM	39	3.232	3.478	3.196	UV cross-linking; immunoblotting in HeLa nuclear extracts with wt and mutated RNAs. pull-down assay
HNRNPK	ENSG00000165119	no	Homo_sapiens	ACCCAU	6	11278705	KH	40	6.906	6.902	6.835	SELEX of 50nt random with recombinant protein. Consensus motif: u/aCCCCCu/a. Winners confirmed by EMSA with recombinant protein.
HNRNPL	ENSG00000104824	no	Homo_sapiens	CACC	4	15889141	RRM	39	2.274	1.891	2.547	Pull-down assays; UV crosslink; immunoprecipitation with HeLa nuclear extracts.
HNRNPL	ENSG00000104824	no	Homo_sapiens	CACC	4	15889141	RRM	39	2.274	1.891	2.547	SELEX of 20nt random with recombinant protein. Consensus motif: ACACACAC. Pull-down assays;UV crosslink; immunoprecipitation with HeLa nuclear extracts.
HNRNPL	ENSG00000104824	no	Homo_sapiens	CCAC	4	15889141	RRM	38	2.234	1.983	2.489	SELEX of 20nt random with recombinant protein. Consensus motif: ACACACAC. Pull-down assays;UV crosslink; immunoprecipitation with HeLa nuclear extracts.
KHSRP	ENSG00000088247	no	Homo_sapiens	UGGG	4	18684992	KH	39	1.307	1.673	1.617	NMR
MBNL1	ENSG00000152601	no	Homo_sapiens	AUGCCA	6	20071745	ZF_CCCH	24	5.951	5.383	5.918	SELEX of 32nt random with recombinant protein. Consensus motifs: YGCY YYYGCUKY.
MBNL1	ENSG00000152601	no	Homo_sapiens	UGCC	4	20071745	ZF_CCCH	25	28126	1.627	2.028	SELEX
MBNL1	ENSG00000152601	no	Homo_sapiens	CUGCCA	6	20071745	ZF_CCCH	7	5.595	5.176	6.068	SELEX of 32nt random with recombinant protein. Consensus motifs: YGCY YYYGCUKY.
NOVA1	ENSG00000139910	no	Homo_sapiens	CCAG	4	17655233	KH	27	46753	0.97	1.644	NMR
NOVA1	ENSG00000139910	no	Homo_sapiens	ACCACC	6	10811881	KH	37	6.286	5.518	6.567	SELEX of 25nt random with recombinant protein. Consensus motif: UCAY. EMSA and Filter Binding Assay with recombinant protein.
NOVA1	ENSG00000139910	no	Homo_sapiens	CCAC	4	17655233	KH	38	2.234	1.983	2.489	NMR
NOVA1	ENSG00000139910	no	Homo_sapiens	CCAU	4	17655233	KH	42	2.446	2.318	2.345	NMR
NOVA2	ENSG00000104967	no	Homo_sapiens	ACCACC	6	10811881	KH	37	6.286	5.518	6.567	SELEX of 25nt random with recombinant protein. Consensus motif: UCAY. EMSA and Filter Binding Assay with recombinant protein.
NUDT21	ENSG00000167005	no	Homo_sapiens	UGUA	4	21295486	NUDIX_2	34;25	3.526	4.268	3.098	X-RAY DIFFRACTION
NUDT21	ENSG00000167005	no	Homo_sapiens	UUGUA	5	20479262	NUDIX_2	33	4.374	5.589	3.841	X-RAY DIFFRACTION
NUDT21	ENSG00000167005	no	Homo_sapiens	UUGUA	5	21295486	NUDIX_2	33	4.374	5.589	3.841	X-RAY DIFFRACTION

NXF1	ENSG00000162231	no	Homo_sapiens	AGAC	4	21822283	LRR_1;NTF2;TAP-RNA_BINE	45	12086	1.992	2.334	X-RAY DIFFRACTION
NXF1	ENSG00000162231	no	Homo_sapiens	CCUG	4	21822283	LRR_1;NTF2;TAP-RNA_BINE	34	41640	0.951	1.558	X-RAY DIFFRACTION
PTBP1	ENSG00000011304	no	Homo_sapiens	CUGA	4	16179478	RRM	35	1.689	1.522	1.816	NMR
PTBP1	ENSG00000011304	no	Homo_sapiens	CUAU	4	16179478	RRM	22	28522	2.877	2.397	NMR
PTBP1	ENSG00000011304	no	Homo_sapiens	CUCC	4	16179478	RRM	51	1.473	1.548	1.803	NMR
PTBP1	ENSG00000011304	no	Homo_sapiens	CUUG	4	16179478	RRM	54	1.971	2.343	1.894	NMR
PTBP1	ENSG00000011304	no	Homo_sapiens	CCUGA	5	16179478	RRM	34	3.506	3.242	3.751	NMR
PTBP1	ENSG00000011304	no	Homo_sapiens	UCUAU	5	16179478	RRM	21	4.513	4.904	4.019	NMR
PTBP1	ENSG00000011304	no	Homo_sapiens	UCUCC	5	16179478	RRM	50	3.374	24167	3.617	NMR
PTBP1	ENSG00000011304	no	Homo_sapiens	CCUUG	5	16179478	RRM	53	3.791	4.075	3.945	NMR
PTBP1	ENSG00000011304	no	Homo_sapiens	CUGC	4	16179478	RRM	7	1.774	1.433	2.265	NMR
PTBP1	ENSG00000011304	no	Homo_sapiens	UCUGC	5	16179478	RRM	6	3.885	3.898	4.123	NMR
RBM28	ENSG00000106344	no	Homo_sapiens	GUGUAGA	7	23846655	RRM	24	9.151	32387	8.844	RNAcompete
RC3H1	ENSG00000135870	no	Homo_sapiens	UUUC	4	25026078	ZF_CCCH	18	1.705	2.665	1.465	X-RAY DIFFRACTION
SRP14	ENSG00000140319	no	Homo_sapiens	GUGG	4	11089964	SRP14	4	1.614	1.477	1.974	X-RAY DIFFRACTION
SRSF1	ENSG00000136450	no	Homo_sapiens	GGAAAGAC ; AC	8	7543047	RRM	41	9.789	9.188	9.935	SELEX of random 20nt with recombinant protein. Consensus motifs: RGAAGAAC. Confirmed by EMSA competition assay with recombinant protein and SELEX winners. UV-crosslinking and immunoprecipitation with Hela nuclear extract or S100 extract.
SRSF1	ENSG00000136450	no	Homo_sapiens	UGGUGAU	7	18841201	RRM	5	7.788	7.327	7.664	genome-wide in vivo immunoprecipitation
SRSF1	ENSG00000136450	no	Homo_sapiens	CCACCCA	7	16825284	RRM	38	7.396	7.361	7.838	SELEX
SRSF2	ENSG00000161547	no	Homo_sapiens	UCCAGU	6	22002536	RRM	20	6.187	5.866	44348	SOLUTION NMR
SRSF3	ENSG00000112081	no	Homo_sapiens	GAUC	4	17036044	RRM	19	2.884	2.436	2.799	NMR spectroscopy
SRSF6	ENSG00000124193	no	Homo_sapiens	GACGUC	6	9649504	RRM	46	8.131	46935	20699	Functional SELEX of constructs with 20nt random by splicing in HeLa S100 extracts complemented by recombinant protein. Consensus motif: USCGKM. UV crosslink; competition and immunoprecipitation assays in HeLa nuclear extracts.
SRSF9	ENSG00000111786	no	Homo_sapiens	AAGAC	5	17548433	RRM	44	4.241	24167	4.223	SELEX of 20nt random with recombinant protein. Consensus motif: AGGAC. EMSA; UV crosslink; SDS-PAGE with HeLa nuclear extracts.
SRSF9	ENSG00000111786	no	Homo_sapiens	UGGAC	5	17548433	RRM	14	43556	3.362	4.762	SELEX of 20nt random with recombinant protein. Consensus motif: AGGAC. EMSA; UV crosslink; SDS-PAGE with HeLa nuclear extracts.
SRSF9	ENSG00000111786	no	Homo_sapiens	ACCAC	5	17548433	RRM	37	4.622	4.098	4.567	SELEX of 20nt random with recombinant protein. Consensus motif: AGGAC. EMSA; UV crosslink; SDS-PAGE with HeLa nuclear extracts.
SSB	ENSG00000138385	no	Homo_sapiens	AUUU	4	18547518	LA;RRM	30	1.525	2.659	1.034	X-RAY DIFFRACTION
SSB	ENSG00000138385	no	Homo_sapiens	UUUU	4	18547518	LA;RRM	31	0.793	2.648	0.257	X-RAY DIFFRACTION
TIA1	ENSG00000116001	no	Homo_sapiens	AUUUUG	6	8576255	RRM	30	5.312	6.165	4.879	SELEX of 68nt random with recombinant protein. Winners confirmed by UV cross-linking and competition assay.
TIA1	ENSG00000116001	no	Homo_sapiens	GUUUC	5	8576255	RRM	17	4.264	4.956	4.043	SELEX of 68nt random with recombinant protein. Winners confirmed by UV cross-linking and competition assay.
TIAL1	ENSG00000151923	no	Homo_sapiens	AUUA	4	8576255	RRM	10	2.189	3.027	43983	SELEX of 68nt random with recombinant protein. Winners confirmed by UV cross-linking and competition assay.
TIAL1	ENSG00000151923	no	Homo_sapiens	AUUUUG	6	8576255	RRM	30	5.312	6.165	4.879	SELEX of 68nt random with recombinant protein. Winners confirmed by UV cross-linking and competition assay.
TIAL1	ENSG00000151923	no	Homo_sapiens	GUUUC	5	8576255	RRM	17	4.264	4.956	4.043	SELEX of 68nt random with recombinant protein. Winners confirmed by UV cross-linking and competition assay.
TRA2B	ENSG00000136527	no	Homo_sapiens	AGAA	4	20926394	RRM	29	1.416	0.95	1.343	SOLUTION NMR
XPO5	ENSG00000124571	no	Homo_sapiens	GUUU	4	19965479	XPO1	17	2.063	2.959	1.767	X-RAY DIFFRACTION
YBX1	ENSG00000065978	no	Homo_sapiens	GAUC	4	22730292	CSD	19	2.884	2.436	2.799	EMSA with recombinant protein.
YBX1	ENSG00000065978	no	Homo_sapiens	CACC	4	22730292	CSD	39	2.274	1.891	2.547	SELEX of 20nt random sequences with recombinant protein. Winners confirmed by EMSA with recombinant protein.
ZFP36	ENSG00000128016	no	Homo_sapiens	CUUGU	5	18367721	ZF_CCCH	54	4.043	4.714	3.905	RIP-chip
ZFP36	ENSG00000128016	no	Homo_sapiens	CCUUGU	6	18367721	ZF_CCCH	53	5.897	6.443	5.902	RIP-chip
ZFP36	ENSG00000128016	no	Homo_sapiens	CUUGU	5	18367721	ZF_CCCH	1	4.358	43895	44652	RIP-chip
ZNF346	ENSG00000113761	no	Homo_sapiens	GUGGU	5	24521053	ZF_C2H2	4	3.946	3.857	3.948	SOLUTION NMR
ZNF346	ENSG00000113761	no	Homo_sapiens	CCACC	5	24521053	ZF_C2H2	38	27089	3.427	43986	SOLUTION NMR

>SL4765-4802 apical loop - marked in red

GGAGAUUUCACUUUGACAUGAACAUCGAGGUUAUUUCC

Gene name	Gene id	Mutated	Organism	Motif	Length	Reference		Domain	Quality_score			Experiments
CELF2	ENSG00000048740	no	Homo_sapiens	CAUCG	5	15657417	RRM	22	6.664	5.325	7.905	SELEX of 20nt random with recombinant protein; some winner sequences are verified by EMSA.
ELAVL1	ENSG00000066044	no	Homo_sapiens	AUUU	4	23519412	RRM	4;32	2.919	4.053	2.428	X-RAY DIFFRACTION
ELAVL2	ENSG00000107105	no	Homo_sapiens	AUUUC	5	8497264	RRM	4;32	5.336	6.076	4.958	SELEX of 25nt random with recombinant protein.
ELAVL4	ENSG00000162374	no	Homo_sapiens	AUUU	4	11175903	RRM	4;32	2.919	4.053	2.428	X-RAY DIFFRACTION

HNRNPL	ENSG00000104824	no	Homo_sapiens	AACA	4	15889141	RRM	20	2.752	2.528	17930	SELEX of 20nt random with recombinant protein. Consensus motif: ACACACAC. Pull-down assays; UV crosslink; immunoprecipitation with HeLa nuclear extracts.
HNRNPL	ENSG00000104824	no	Homo_sapiens	ACAU	4	15889141	RRM	21;15	32568	3.917	3.545	SELEX of 20nt random with recombinant protein. Consensus motif: ACACACAC. Pull-down assays; UV crosslink; immunoprecipitation with HeLa nuclear extracts.
NOVA1	ENSG00000139910	no	Homo_sapiens	UCAC	4	10811881	KH	7	34731	2.948	2.822	SELEX
NOVA1	ENSG00000139910	no	Homo_sapiens	UCAC	4	17655233	KH	7	34731	2.948	2.822	NMR
NOVA2	ENSG00000104967	no	Homo_sapiens	UCAC	4	10811881	KH	7	34731	2.948	2.822	SELEX
OAS1	ENSG00000089127	no	Homo_sapiens	UUGA	4	23319625	OAS1_C;NTP_TRANSF_2	12	25965	2.923	2.367	X-RAY DIFFRACTION
PHAX	ENSG00000164902	no	Homo_sapiens	AUCG	4	20430857	RNA_GG_BIND	23	5.253	4.219	5.818	SOLUTION NMR
PPIE	ENSG00000084072	no	Homo_sapiens	UUAUUU	6	18258190	RRM;PRO_ISOMERASE	30	4.984	11505	4.426	single RNA affinity purification
PTBP1	ENSG00000011304	no	Homo_sapiens	CUUU	4	16179478	RRM	10	2.068	2.839	1.855	NMR
RC3H1	ENSG00000135870	no	Homo_sapiens	UUUC	4	25026078	ZF_CCCH	5;33	3.099	43986	2.859	X-RAY DIFFRACTION
SRP54	ENSG00000100883	no	Homo_sapiens	AGGU	4	12244299	SRP54; SRP_SPB	27	2.625	3.081	2.806	X-RAY DIFFRACTION
SRSF2	ENSG00000161547	no	Homo_sapiens	GGAGAU	6	23846655	RRM	0	6.542	5.844	6.707	RNAcompete
SRSF3	ENSG00000112081	no	Homo_sapiens	CAUC	4	17036044	RRM	22	2.921	2.375	3.082	NMR spectroscopy
SRSF3	ENSG00000112081	no	Homo_sapiens	UUCAC	5	17036044	RRM	6	4.889	4.967	4.628	NMR spectroscopy
SRSF9	ENSG00000111786	no	Homo_sapiens	AUGAACA	7	23846655	RRM	17	8.575	8.015	8.303	RNAcompete
SSB	ENSG00000138385	no	Homo_sapiens	AUUU	4	18547518	LA;RRM	4;32	2.919	4.053	2.428	X-RAY DIFFRACTION
TIA1	ENSG00000116001	no	Homo_sapiens	AUUUC	5	8576255	RRM	4;32	5.336	6.076	4.958	SELEX of 68nt random with recombinant protein. Winners confirmed by UV cross-linking and competition assay.
TIA1	ENSG00000116001	no	Homo_sapiens	CUUUG	5	8576255	RRM	10	4.129	4.321	4.028	SELEX of 68nt random with recombinant protein. Winners confirmed by UV cross-linking and competition assay.
TIAL1	ENSG00000151923	no	Homo_sapiens	AUUUC	5	8576255	RRM	4;32	5.336	6.076	4.958	SELEX of 68nt random with recombinant protein. Winners confirmed by UV cross-linking and competition assay.
TIAL1	ENSG00000151923	no	Homo_sapiens	CUUUG	5	8576255	RRM	10	4.129	4.321	4.028	SELEX of 68nt random with recombinant protein. Winners confirmed by UV cross-linking and competition assay.
YBX1	ENSG00000065978	no	Homo_sapiens	AACAUC	6	23846655	CSD	20	7.056	6.348	7.057	RNAcompete
YBX1	ENSG00000065978	no	Homo_sapiens	AACAUCG	7	23846655	CSD	20	10.956	19603	11.775	RNAcompete
YBX1	ENSG00000065978	no	Homo_sapiens	CAUC	4	22730292	CSD	22	2.921	2.375	3.082	In vivo splicing in HEK-293 cells with wt and mutated sequences; EMSA
YBX1	ENSG00000065978	no	Homo_sapiens	CAUC	4	22730292	CSD	22	2.921	2.375	3.082	SELEX of 20nt random sequences with recombinant protein. Winners confirmed by EMSA with recombinant protein.
YBX2	ENSG00000006047	no	Homo_sapiens	AACAUCG	7	23846655	CSD	20	10.956	19603	11.775	RNAcompete
ZRANB2	ENSG00000132485	no	Homo_sapiens	AGGUUA	6	19304800	ZF_RANBP	27	7.468	8.157	7.154	SELEX of 25nt random with recombinant protein. Consensus motifs: CAGGUAA and AAGGUAA. Fluorescence anisotropy.
ZRANB2	ENSG00000132485	no	Homo_sapiens	GAGGUU	6	19304800	ZF_RANBP	26	6.836	7.305	6.454	SELEX of 25nt random with recombinant protein. Consensus motifs: CAGGUAA and AAGGUAA.
NOVA1	ENSG00000139910	no	Homo_sapiens	UCAC	4	15367696	KH	7	34731	2.948	2.822	RNA affinity binding assays

>SL6236-6260 apical loop - marked in red

GGCGCUUGAUCUAACAACGAGUGCC

Gene name	Gene id	Mutated	Organism	Motif	Length	Reference	Domain		Quality_score			Experiments
ADAR	ENSG00000160710	no	Homo_sapiens	GCGC	4	17437712	A_DEAMIN;DSRM;Z-ALPHA	1	4.668	4.251	6.201	X-RAY DIFFRACTION
CMTR1	ENSG00000137200	no	Homo_sapiens	GAUC	4	24402442	FTSJ;G-PATCH	7	4.258	3.809	4.172	X-RAY DIFFRACTION
DDX58	ENSG00000107201	no	Homo_sapiens	GGCG	4	20961956	DEAD	0	4.272	4.063	5.425	X-RAY DIFFRACTION
DHX9	ENSG00000135829	no	Homo_sapiens	GCGC	4	23361462	DEAD;DSRM;HA2;HELICASE	1	4.668	4.251	6.201	X-RAY DIFFRACTION
HNRNPL	ENSG00000104824	no	Homo_sapiens	AACA	4	15889141	RRM	12	3.422	3.198	42430	SELEX of 20nt random with recombinant protein. Consensus motif: ACACACAC. Pull-down assays, UV crosslink, immunoprecipitation with HeLa nuclear extracts.
HNRNPL	ENSG00000104824	no	Homo_sapiens	ACAA	4	15889141	RRM	13	3.634	3.242	3.354	Pull-down assays, UV crosslink, immunoprecipitation with HeLa nuclear extracts.
MBNL1	ENSG00000152601	no	Homo_sapiens	GCGCUU	6	20071745	ZF_CCCH	1	9.344	8.761	10.626	SELEX of 32nt random with recombinant protein. Consensus motifs: YGCY, YYYGCUKY.
MBNL1	ENSG00000152601	no	Homo_sapiens	UGCC	4	20071745	ZF_CCCH	21	3.143	3.0	3.402	SELEX
MBNL1	ENSG00000152601	no	Homo_sapiens	CGCU	4	20071745	ZF_CCCH	2	5.0	4.398	6.154	SELEX
OAS1	ENSG00000089127	no	Homo_sapiens	UUGA	4	23319625	OAS1_C;NTP_TRANSF_2	5	13940	3.593	3.037	X-RAY DIFFRACTION
PIWIL1	ENSG00000125207	no	Homo_sapiens	GCUU	4	21193640	GAGE;PAZ;PIWI	3	3.496	44015	19054	X-RAY DIFFRACTION
PTBP1	ENSG00000011304	no	Homo_sapiens	CUAA	4	16179478	RRM	10	4.052	4.734	3.658	NMR
PTBP1	ENSG00000011304	no	Homo_sapiens	CUUG	4	16179478	RRM	4	3.345	3.716	3.267	NMR
PTBP1	ENSG00000011304	no	Homo_sapiens	UCUAA	5	16179478	RRM	9	5.838	6.778	5.441	NMR
QKI	ENSG00000112531	yes	Homo_sapiens	CGAGU	5	24722255	KH	17	7.504	24259	8.105	EMSA with WT and mutated sequences and UV crosslinking with recombinant protein
SRSF3	ENSG00000112081	no	Homo_sapiens	GAUC	4	17036044	RRM	7	4.258	3.809	4.172	NMR spectroscopy
YBX1	ENSG00000065978	no	Homo_sapiens	ACAAC	5	18503770	CSD	13	6.206	5.427	43617	UV crosslink, EMSA, and pull-down assays with HeLa nuclear extracts.
YBX1	ENSG00000065978	no	Homo_sapiens	GAUC	4	22730292	CSD	7	4.258	3.809	4.172	EMSA with recombinant protein.
YBX2	ENSG00000006047	no	Homo_sapiens	AACAACG	7	23846655	CSD	12	11.826	10.503	12.554	RNAcompete
YTHDC1	ENSG00000083896	no	Homo_sapiens	GAGUGC	6	20167602	YTH	18	7.532	7.553	7.341	SELEX

>SL6289-6308 apical loop - marked in red

AGAAGAGGGACAUCCUCUCU

Gene name	Gene id	Mutated	Organism	Motif	Length	Reference	Domain		Quality_score			Experiments
F2	ENSG00000180210	no	Homo_sapiens	GAAG	4	18971322	GLA;KRINGLE;THROMBIN_L	1	3.411	2.742	3.734	X-RAY DIFFRACTION
GRSF1	ENSG00000132463	no	Homo_sapiens	AGGGA	5	17337441	RRM	5	5.285	5.689	5.563	protein affinity purification
HNRNPF	ENSG00000169813	no	Homo_sapiens	AGGGA	5	11571276	RRM;ZF_RNPHF	5	5.285	5.689	5.563	RNA affinity binding assays, immunoblot with WT HeLa nuclear extracts or depleted by RNA affinity chromatography.
HNRNPF	ENSG00000169813	no	Homo_sapiens	AGGGA	5	17337441	RRM;ZF_RNPHF	5	5.285	5.689	5.563	RNA-affinity chromatography assays with HeLa nuclear extract and immunoblotting.
HNRNPF	ENSG00000169813	no	Homo_sapiens	AGGGA	5	17337441	RRM;ZF_RNPHF	5	5.285	5.689	5.563	protein affinity purification
HNRNPF	ENSG00000169813	yes	Homo_sapiens	AGGGA	5	19404736	RRM;ZF_RNPHF	5	5.285	5.689	5.563	Mutational analysis, RNA affinity chromatography, UV cross-linking, immunoprecipitation with HeLa nuclear extract.
HNRNPH1	ENSG00000169045	no	Homo_sapiens	AGAGG	5	17337441	ZF_RNPHF	3	5.171	5.035	19115	RNA-affinity chromatography assays with HeLa nuclear extract and immunoblotting.
HNRNPH1	ENSG00000169045	no	Homo_sapiens	AGGGA	5	20028692	ZF_RNPHF	5	5.285	5.689	5.563	In vivo splicing in HEK293T and HMVEC using wt or mutated constructs.
HNRNPH1	ENSG00000169045	no	Homo_sapiens	AGGGA	5	11571276	ZF_RNPHF	5	5.285	5.689	5.563	RNA affinity binding assays, immunoblot with WT HeLa nuclear extracts or depleted by RNA affinity chromatography.
HNRNPH1	ENSG00000169045	no	Homo_sapiens	AGGGA	5	17337441	ZF_RNPHF	5	5.285	5.689	5.563	RNA-affinity chromatography assays with HeLa nuclear extract and immunoblotting.
HNRNPH1	ENSG00000169045	no	Homo_sapiens	AGGGA	5	17337441	ZF_RNPHF	5	5.285	5.689	5.563	protein affinity purification
HNRNPH1	ENSG00000169045	yes	Homo_sapiens	AGGGA	5	19404736	ZF_RNPHF	5	5.285	5.689	5.563	Mutational analysis, RNA affinity chromatography, UV cross-linking, immunoprecipitation with HeLa nuclear extract.
HNRNPH2	ENSG00000126945	no	Homo_sapiens	AGAGG	5	17337441	ZF_RNPHF	3	5.171	5.035	19115	RNA-affinity chromatography assays with HeLa nuclear extract and immunoblotting.
HNRNPH2	ENSG00000126945	no	Homo_sapiens	AGGGA	5	20028692	ZF_RNPHF	5	5.285	5.689	5.563	In vivo splicing in HEK293T and HMVEC using wt or mutated constructs.
HNRNPH2	ENSG00000126945	no	Homo_sapiens	AGGGA	5	11571276	ZF_RNPHF	5	5.285	5.689	5.563	RNA affinity binding assays, immunoblot with WT HeLa nuclear extracts or depleted by RNA affinity chromatography.
HNRNPH2	ENSG00000126945	no	Homo_sapiens	AGGGA	5	17337441	ZF_RNPHF	5	5.285	5.689	5.563	RNA-affinity chromatography assays with HeLa nuclear extract and immunoblotting.
HNRNPH2	ENSG00000126945	no	Homo_sapiens	AGGGA	5	17337441	ZF_RNPHF	5	5.285	5.689	5.563	protein affinity purification
HNRNPH2	ENSG00000126945	yes	Homo_sapiens	AGGGA	5	19404736	ZF_RNPHF	5	5.285	5.689	5.563	Mutational analysis, RNA affinity chromatography, UV cross-linking, immunoprecipitation with HeLa nuclear extract.
HNRNPH3	ENSG00000096746	no	Homo_sapiens	AGGGA	5	20028692	RRM	5	5.285	5.689	5.563	In vivo splicing in HEK293T and HMVEC using wt or mutated constructs.
HNRNPH3	ENSG00000096746	no	Homo_sapiens	AGGGA	5	11571276	RRM	5	5.285	5.689	5.563	RNA affinity binding assays, immunoblot with WT HeLa nuclear extracts or depleted by RNA affinity chromatography.
HNRNPH3	ENSG00000096746	no	Homo_sapiens	AGGGA	5	17337441	RRM	5	5.285	5.689	5.563	RNA-affinity chromatography assays with HeLa nuclear extract and immunoblotting.
HNRNPH3	ENSG00000096746	no	Homo_sapiens	AGGGA	5	17337441	RRM	5	5.285	5.689	5.563	protein affinity purification
HNRNPH3	ENSG00000096746	yes	Homo_sapiens	AGGGA	5	19404736	RRM	5	5.285	5.689	5.563	Mutational analysis, RNA affinity chromatography, UV cross-linking, immunoprecipitation with HeLa nuclear extract.
HNRNPL	ENSG00000104824	no	Homo_sapiens	ACAU	4	15889141	RRM	9	3.932	3.958	3.587	SELEX of 20nt random with recombinant protein. Consensus motif: ACACACAC. Pull-down assays, UV crosslink, immunoprecipitation with HeLa nuclear extracts.
KHSRP	ENSG00000088247	no	Homo_sapiens	AGGG	4	18684992	KH	5	3.392	3.853	32568	NMR
NONO	ENSG00000147140	no	Homo_sapiens	AGGGA	5	9001221	RRM	5	5.285	5.689	5.563	SELEX
PABPN1	ENSG00000100836	no	Homo_sapiens	AGAAGA	6	23846655	RRM	0	6.793	5.711	7.063	RNAcompete
PTBP1	ENSG00000011304	no	Homo_sapiens	CUCU	4	16179478	RRM	16;14	4.239	4.626	4.358	SOLUTION NMR
PTBP1	ENSG00000011304	no	Homo_sapiens	CUCUC	5	19861426	RRM	14	5.239	5.778	21306	in vitro RNA pulldown
PTBP1	ENSG00000011304	no	Homo_sapiens	CUCUCU	6	10911989	PTBP1	14	6.719	7.722	6.863	Immunodepletion, UV crosslink in WERI-1 and HeLa extracts, immunoprecipitation, immunoblot.
PTBP1	ENSG00000011304	no	Homo_sapiens	CUCUCU	6	11788707	RRM	14	6.719	7.722	6.863	NMR
PTBP1	ENSG00000011304	no	Homo_sapiens	CUCUCU	6	16179478	PTBP1	14	6.719	7.722	6.863	NMR, EMSA with recombinant protein
PTBP1	ENSG00000011304	no	Homo_sapiens	CUCUCU	6	11003644	PTBP1	14	6.719	7.722	6.863	RNA affinity purification with HeLa or WERI-1 nuclear extracts. UV crosslink in HeLa and in WERI-1 extracts. EMSA with purified protein. Immunoprecipitation and Western blot.
PTBP1	ENSG00000011304	no	Homo_sapiens	CUCUCU	6	16179478	RRM	14	6.719	7.722	6.863	SOLUTION NMR
PTBP1	ENSG00000011304	no	Homo_sapiens	CUCUCU	6	9234723	PTBP1	14	6.719	7.722	6.863	UV crosslink in HeLa and WERI-1 nuclear extracts, competition assay, immunoprecipitation. EMSA supershift in HeLa nuclear extracts.
PTBP1	ENSG00000011304	no	Homo_sapiens	CUCU	4	16179478	RRM	16;14	4.239	4.626	4.358	NMR
PTBP1	ENSG00000011304	no	Homo_sapiens	UCUCU	5	11788707	PTBP1	15	4.925	5.809	4.858	Chemical shift mapping (NMR)
PTBP1	ENSG00000011304	no	Homo_sapiens	UCUCU	5	11788707	RRM	15	4.925	5.809	4.858	NMR
PTBP1	ENSG00000011304	no	Homo_sapiens	UCUCU	5	16179478	RRM	15	4.925	5.809	4.858	SOLUTION NMR
PTBP1	ENSG00000011304	no	Homo_sapiens	CCUCU	5	16179478	RRM	13	44017	5.383	5.456	NMR
PTBP1	ENSG00000011304	no	Homo_sapiens	UCUCU	5	16179478	RRM	15	4.925	5.809	4.858	NMR
PTBP2	ENSG00000117569	no	Homo_sapiens	CUCUCU	6	11003644	RRM	14	6.719	7.722	6.863	RNA affinity purification with HeLa or WERI-1 nuclear extracts. UV crosslink in HeLa and in WERI-1 extracts. EMSA with purified protein. Immunoprecipitation and Western blot.
SRP19	ENSG00000153037	no	Homo_sapiens	CCUC	4	20179341	SRP19	13	3.219	3.298	3.553	X-RAY DIFFRACTION
SRP54	ENSG00000100883	no	Homo_sapiens	CCUC	4	12244299	SRP54,SRP_SPB	13	3.219	3.298	3.553	X-RAY DIFFRACTION
SRP68	ENSG00000167881	no	Homo_sapiens	CCUC	4	24700861	SRP68	13	3.219	3.298	3.553	X-RAY DIFFRACTION
SRSF10	ENSG00000188529	no	Homo_sapiens	AGAGGGA	7	23846655	RRM	3	8.958	9.248	9.175	RNAcompete
SRSF2	ENSG00000161547	yes	Homo_sapiens	AGAAG	5	11847131	RRM	0	43956	44652	5.334	RNA affinity chromatography assay and immunoblot.
SRSF3	ENSG00000112081	no	Homo_sapiens	CAUC	4	17036044	RRM	10	3.963	3.417	4.124	NMR spectroscopy

SRSF5	ENSG00000100650	no	Homo_sapiens	AGAGG	5	9649504	RRM	3	5.171	5.035	19115	Functional SELEX of constructs with 20nt random by splicing in HeLa S100 extracts complemented by recombinant protein. Consensus motif: ACDGS. UV crosslink, competition and immunoprecipitation assays in HeLa nuclear extracts.
TRA2B	ENSG00000136527	no	Homo_sapiens	AGAA	4	20926394	RRM	0	3.162	2.695	3.088	SOLUTION NMR
YBX1	ENSG00000065978	no	Homo_sapiens	CAUC	4	22730292	CSD	10	3.963	3.417	4.124	In vivo splicing in HEK-293 cells with wt and mutated sequences, EMSA
YBX1	ENSG00000065978	no	Homo_sapiens	CAUC	4	22730292	CSD	10	3.963	3.417	4.124	SELEX of 20nt random sequences with recombinant protein. Winners confirmed by EMSA with recombinant protein.
ZFP36	ENSG00000128016	no	Homo_sapiens	UCCUCU	6	18367721	ZF_CCCH	12	6.943	7.394	7.235	RIP-chip
HNRNPH1	ENSG00000169045	no	Homo_sapiens	GGGA	4	11847131;9343212	ZF_RNPHF	6	3.344	3.529	3.691	snRNA inactivation in HeLa cell nuclear extract;Western blot

>SL6376-6399 apical loop - marked in red

UGACUUAUGUAAAAGAUGAGCUCA

Gene name	Gene id	Mutated	Organism	Motif	Length	Reference		Domain	Quality_score			Experiments
CELF2	ENSG00000048740	no	Homo_sapiens	UAUGU	5	15657417	RRM	5	5.516	6.376	5.066	SELEX of 20nt random with recombinant protein, some winner sequences are verified by EMSA.
NUDT21	ENSG00000167005	no	Homo_sapiens	UGUA	4	21295486	NUDIX_2	7	3.657	4.399	3.229	X-RAY DIFFRACTION
PABPN1	ENSG00000100836	no	Homo_sapiens	AAAAGA	6	23846655	RRM	10	6.466	6.226	6.044	RNAcompete
PTBP1	ENSG00000011304	no	Homo_sapiens	CUUA	4	16179478	RRM	3	3.943	4.659	3.631	NMR
PTBP1	ENSG00000011304	no	Homo_sapiens	CUCA	4	16179478	RRM	20	3.221	3.196	3.152	NMR
QKI	ENSG00000112531	no	Homo_sapiens	ACUUAU	6	24722255	KH	2	8.136	8.849	7.647	EMSA with WT and mutated sequences with recombinant protein
RNASEL	ENSG00000135828	no	Homo_sapiens	AAAA	4	24578532	ANK;PKINASE;RIBONUC_2	10	2.379	2.811	1.833	X-RAY DIFFRACTION
SF1	ENSG00000168066	no	Homo_sapiens	ACUUAU	6	24722255	KH;ZF_CCHC	2	8.136	8.849	7.647	UV crosslinking with recombinant protein
SRP19	ENSG00000153037	no	Homo_sapiens	GAGC	4	11641499	SRP19	17	3.427	2.988	3.935	X-RAY DIFFRACTION

>SL6476-6495 apical loop - marked in red

ACAGACAUUUGGUAAUCUGU

Gene name	Gene id	Mutated	Organism	Motif	Length	Reference		Domain	Quality_score			Experiments
ELAVL1	ENSG00000066044	no	Homo_sapiens	AUUU	4	23519412	RRM	6	2.961	4.095	17199	X-RAY DIFFRACTION
ELAVL2	ENSG00000107105	no	Homo_sapiens	AUUUG	5	8497264	RRM	6	5.408	6.055	5.039	SELEX of 25nt random with recombinant protein.
ELAVL4	ENSG00000162374	no	Homo_sapiens	AUUU	4	11175903	RRM	6	2.961	4.095	17199	X-RAY DIFFRACTION
HNRNPL	ENSG00000104824	no	Homo_sapiens	ACAU	4	15889141	RRM	4	3.932	3.958	3.587	SELEX of 20nt random with recombinant protein. Consensus motif: ACACACAC. Pull-down assays, UV crosslink, immunoprecipitation with HeLa nuclear extracts.
IGF2BP3	ENSG00000136231	no	Homo_sapiens	CAUU	4	20371350	KH;RRM	5	3.665	44047	3.307	PAR-clip
NXF1	ENSG00000162231	no	Homo_sapiens	AGAC	4	21822283	LRR_1;NTF2;TAP-RNA_BINI	2	4.076	3.738	4.079	X-RAY DIFFRACTION
PTBP1	ENSG00000011304	no	Homo_sapiens	CUGU	4	16179478	RRM	16	3.302	3.525	12114	NMR
PTBP1	ENSG00000011304	no	Homo_sapiens	UCUGU	5	16179478	RRM	15	44170	30072	4.958	NMR
SSB	ENSG00000138385	no	Homo_sapiens	AUUU	4	18547518	LA;RRM	6	2.961	4.095	17199	X-RAY DIFFRACTION
ZRANB2	ENSG00000132485	no	Homo_sapiens	UGGUAA	6	19304800	ZF_RANBP	9	35977	9.036	8.0	SELEX of 25nt random with recombinant protein. Consensus motifs: CAGGUAA and AAGGUAA.

>SL6581-6597 apical loop - marked in red

GAUGUUAGAUGGACAUC

Gene name	Gene id	Mutated	Organism	Motif	Length	Reference		Domain	Quality_score			Experiments
CELF1	ENSG00000149187	no	Homo_sapiens	UGUU	4	20947024	RRM	2	3.643	4.416	3.363	X-RAY DIFFRACTION
CELF2	ENSG00000048740	no	Homo_sapiens	AUGUU	5	15657417	RRM	1	5.822	6.404	5.437	SELEX of 20nt random with recombinant protein, some winner sequences are verified by EMSA.
CELF2	ENSG00000048740	no	Homo_sapiens	UGUU	4	PubMed ID is not availa	RRM	2	3.643	4.416	3.363	X-RAY DIFFRACTION
HNRNPL	ENSG00000104824	no	Homo_sapiens	ACAU	4	15889141	RRM	12	4.212	4.239	3.867	SELEX of 20nt random with recombinant protein. Consensus motif: ACACACAC. Pull-down assays, UV crosslink, immunoprecipitation with HeLa nuclear extracts.
KHSRP	ENSG00000088247	no	Homo_sapiens	UUAG	4	18684992	KH	4	4.542	27515	4.057	NMR
SRSF1	ENSG00000136450	no	Homo_sapiens	AGAUGGAC	8	7543047	RRM	6	12.336	11.412	13.008	SELEX of random 20nt with recombinant protein. Consensus motifs: RGAAGAAC, ACGCGCA and AGGACAGAGC. Confirmed by EMSA, competition assay with recombinant protein and SELEX winners. UV-crosslinking and immunoprecipitation with Hela nuclear extract or S100 extract.
SRSF3	ENSG00000112081	no	Homo_sapiens	CAUC	4	17036044	RRM	13	4.243	3.697	4.404	NMR spectroscopy
SRSF9	ENSG00000111786	no	Homo_sapiens	UGGAC	5	17548433	RRM	9	6.297	5.469	6.869	SELEX of 20nt random with recombinant protein. Consensus motif: AGGAC. EMSA, UV crosslink, SDS-PAGE with HeLa nuclear extracts.
YBX1	ENSG00000065978	no	Homo_sapiens	CAUC	4	22730292	CSD	13	4.243	3.697	4.404	In vivo splicing in HEK-293 cells with wt and mutated sequences, EMSA
YBX1	ENSG00000065978	no	Homo_sapiens	CAUC	4	22730292	CSD	13	4.243	3.697	4.404	SELEX of 20nt random sequences with recombinant protein. Winners confirmed by EMSA with recombinant protein.
SRSF2	ENSG00000161547	no	Homo_sapiens	UUAG	4	10022872;9710624;762	RRM	4	4.542	27515	4.057	In vitro splicing assays and RNA competition assays
HNRNPA1	ENSG00000135486	no	Homo_sapiens	UUAG	4	10022872;9710624;762	RRM	4	4.542	27515	4.057	In vitro splicing assays and RNA competition assays

>SL6689-6722 apical loop - marked in red

GCACAAAGAGACAAACUACAUUGACUACUUGUGC

Gene name	Gene id	Mutated	Organism	Motif	Length	Reference		Domain	Quality_score			Experiments
CELF1	ENSG00000149187	no	Homo_sapiens	UUGUG	5	20947024	RRM	28	4.675	5.085	4.515	X-RAY DIFFRACTION
FXR2	ENSG00000129245	no	Homo_sapiens	AGACAAA	7	23846655	KH	8	8.558	8.191	46235	RNAcompete
HNRNPH1	ENSG00000169045	no	Homo_sapiens	AGAGA	5	17337441	ZF_RNPHF	6	4.243	36220	4.084	RNA-affinity chromatography assays with HeLa nuclear extract and immunoblotting.

HNRNPH2	ENSG00000126945	no	Homo_sapiens	AGAGA	5	17337441	ZF_RNPHF	6	4.243	36220	4.084	RNA-affinity chromatography assays with HeLa nuclear extract and immunoblotting.
HNRNPL	ENSG00000104824	no	Homo_sapiens	ACAA	4	15889141	RRM	10;2	4.139	3.747	31472	Pull-down assays, UV crosslink, immunoprecipitation with HeLa nuclear extracts.
HNRNPL	ENSG00000104824	no	Homo_sapiens	ACAU	4	15889141	RRM	17	3.066	3.092	26330	SELEX of 20nt random with recombinant protein. Consensus motif: ACACACAC. Pull-down assays, UV crosslink, immunoprecipitation with HeLa nuclear extracts.
HNRNPL	ENSG00000104824	no	Homo_sapiens	CACA	4	15889141	RRM	1	2.816	27426	27061	SELEX of 20nt random with recombinant protein. Consensus motif: ACACACAC. Pull-down assays, UV crosslink, immunoprecipitation with HeLa nuclear extracts.
HNRNPL	ENSG00000104824	no	Homo_sapiens	GCAC	4	15889141	RRM	0	3.379	3.086	3.581	SELEX of 20nt random with recombinant protein. Consensus motif: ACACACAC. Pull-down assays, UV crosslink, immunoprecipitation with HeLa nuclear extracts.
HNRNPL	ENSG00000104824	no	Homo_sapiens	UACA	4	15889141	RRM	16	3.282	3.233	2.885	SELEX of 20nt random with recombinant protein. Consensus motif: ACACACAC. Pull-down assays, UV crosslink, immunoprecipitation with HeLa nuclear extracts.
IGF2BP3	ENSG00000136231	no	Homo_sapiens	CAUU	4	20371350	KH;RRM	18	2.799	3.213	16103	PAR-clip
NXF1	ENSG00000162231	no	Homo_sapiens	AGAC	4	21822283	LRR_1;NTF2;TAP-RNA_BIND	8	3.209	2.871	3.212	X-RAY DIFFRACTION
OAS1	ENSG00000089127	no	Homo_sapiens	UUGA	4	23319625	OAS1_C;NTP_TRANSF_2	20	2.885	3.098	2.542	X-RAY DIFFRACTION
PTBP1	ENSG00000011304	no	Homo_sapiens	CUAC	4	16179478	RRM	15;24	4.732	4.173	4.721	NMR
PTBP1	ENSG00000011304	no	Homo_sapiens	CUUG	4	16179478	RRM	27	31079	3.221	2.773	NMR
RBMY1A1	ENSG00000234414	no	Homo_sapiens	CACAA	5	17318228	RRM	1	5.312	4.987	5.078	SELEX
SRSF10	ENSG00000188529	no	Homo_sapiens	AGACAA	6	18794844	RRM	8	6.988	18050	6.812	EMSA
SRSF10	ENSG00000188529	no	Homo_sapiens	AGACAA	6	18794844	RRM	8	6.988	18050	6.812	EMSA and RNase protection assay with recombinant protein.
SRSF10	ENSG00000188529	no	Homo_sapiens	AGAGACA	7	23846655	RRM	6	8.275	8.222	7.779	RNAcompete
SRSF10	ENSG00000188529	no	Homo_sapiens	AGAGACA	7	23846655	RRM	6	8.275	8.222	7.779	RNAcompete
SRSF10	ENSG00000188529	no	Homo_sapiens	AAAGAGA	7	23846655	RRM	4	7.881	7.435	43989	RNAcompete
SRSF10	ENSG00000188529	no	Homo_sapiens	GACAAA	6	18794844	RRM	9	7.101	6.623	6.872	EMSA
SRSF10	ENSG00000188529	no	Homo_sapiens	GACAAA	6	18794844	RRM	9	7.101	6.623	6.872	EMSA and RNase protection assay with recombinant protein.
YTHDC1	ENSG00000083896	no	Homo_sapiens	GACUAC	6	20167602	YTH	22	8.085	7.301	7.782	SELEX
ZFP36	ENSG00000128016	no	Homo_sapiens	CUUGU	5	18367721	ZF_CCCH	27	4.943	5.615	4.805	RIP-chip
ZFP36	ENSG00000128016	no	Homo_sapiens	ACUUGU	6	18367721	ZF_CCCH	26	7.223	7.805	43928	RIP-chip
ZFP36	ENSG00000128016	no	Homo_sapiens	UUGUGC	6	18367721	ZF_CCCH	28	7.191	7.467	7.095	RIP-chip

>SL6738-6761 apical loop - marked in red

UACAGGGAUAAACAUUACUUUGUG

Gene name	Gene id	Mutated	Organism	Motif	Length	Reference	Domain		Quality_score			Experiments
CELF1	ENSG00000149187	no	Homo_sapiens	UUGUG	5	20947024	RRM	19	46143	24593	43835	X-RAY DIFFRACTION
ESRP1	ENSG00000104413	no	Homo_sapiens	AGGGAU	6	20526337	RRM	3	7.662	8.107	7.644	NMR spectroscopy
GRSF1	ENSG00000132463	no	Homo_sapiens	AGGGA	5	17337441	RRM	3	4.963	5.367	5.241	protein affinity purification
HNRNPA1	ENSG00000135486	no	Homo_sapiens	CAGGGA	6	7510636	RRM	2	6.617	6.785	7.126	SELEX of 20nt random sequences with recombinant protein. Winners confirmed by filter binding assays with purified protein and confirmed by UV-crosslink and immunoblot with HeLa nuclear extract. Consensus motif: UAGGGA/U.
HNRNPA1	ENSG00000135486	no	Homo_sapiens	CAGGGA	6	11598017	RRM	2	6.617	6.785	7.126	UV crosslink, immunoblot, EMSA.
HNRNPF	ENSG00000169813	no	Homo_sapiens	AGGGA	5	11571276	RRM;ZF_RNPHF	3	4.963	5.367	5.241	RNA affinity binding assays, immunoblot with WT HeLa nuclear extracts or depleted by RNA affinity chromatography.
HNRNPF	ENSG00000169813	no	Homo_sapiens	AGGGA	5	17337441	RRM;ZF_RNPHF	3	4.963	5.367	5.241	RNA-affinity chromatography assays with HeLa nuclear extract and immunoblotting.
HNRNPF	ENSG00000169813	no	Homo_sapiens	AGGGAU	6	20526337	RRM;ZF_RNPHF	3	7.662	8.107	7.644	NMR spectroscopy
HNRNPF	ENSG00000169813	no	Homo_sapiens	AGGGAU	6	23275549	RRM;ZF_RNPHF	3	7.662	8.107	7.644	NMR spectroscopy, isothermal titration calorimetry
HNRNPF	ENSG00000169813	no	Homo_sapiens	AGGGA	5	17337441	RRM;ZF_RNPHF	3	4.963	5.367	5.241	protein affinity purification
HNRNPF	ENSG00000169813	yes	Homo_sapiens	AGGGA	5	19404736	RRM;ZF_RNPHF	3	4.963	5.367	5.241	Mutational analysis, RNA affinity chromatography, UV cross-linking, immunoprecipitation with HeLa nuclear extract.
HNRNPH1	ENSG00000169045	no	Homo_sapiens	AGGGA	5	20028692	ZF_RNPHF	3	4.963	5.367	5.241	In vivo splicing in HEK293T and HMVEC using wt or mutated constructs.
HNRNPH1	ENSG00000169045	no	Homo_sapiens	AGGGA	5	11571276	ZF_RNPHF	3	4.963	5.367	5.241	RNA affinity binding assays, immunoblot with WT HeLa nuclear extracts or depleted by RNA affinity chromatography.
HNRNPH1	ENSG00000169045	no	Homo_sapiens	AGGGA	5	17337441	ZF_RNPHF	3	4.963	5.367	5.241	RNA-affinity chromatography assays with HeLa nuclear extract and immunoblotting.
HNRNPH1	ENSG00000169045	no	Homo_sapiens	AGGGA	5	17337441	ZF_RNPHF	3	4.963	5.367	5.241	protein affinity purification
HNRNPH1	ENSG00000169045	yes	Homo_sapiens	AGGGA	5	19404736	ZF_RNPHF	3	4.963	5.367	5.241	Mutational analysis, RNA affinity chromatography, UV cross-linking, immunoprecipitation with HeLa nuclear extract.
HNRNPH2	ENSG00000126945	no	Homo_sapiens	AGGGA	5	20028692	ZF_RNPHF	3	4.963	5.367	5.241	In vivo splicing in HEK293T and HMVEC using wt or mutated constructs.
HNRNPH2	ENSG00000126945	no	Homo_sapiens	AGGGA	5	11571276	ZF_RNPHF	3	4.963	5.367	5.241	RNA affinity binding assays, immunoblot with WT HeLa nuclear extracts or depleted by RNA affinity chromatography.
HNRNPH2	ENSG00000126945	no	Homo_sapiens	AGGGA	5	17337441	ZF_RNPHF	3	4.963	5.367	5.241	RNA-affinity chromatography assays with HeLa nuclear extract and immunoblotting.
HNRNPH2	ENSG00000126945	no	Homo_sapiens	AGGGA	5	17337441	ZF_RNPHF	3	4.963	5.367	5.241	protein affinity purification
HNRNPH2	ENSG00000126945	yes	Homo_sapiens	AGGGA	5	19404736	ZF_RNPHF	3	4.963	5.367	5.241	Mutational analysis, RNA affinity chromatography, UV cross-linking, immunoprecipitation with HeLa nuclear extract.
HNRNPH3	ENSG00000096746	no	Homo_sapiens	AGGGA	5	20028692	RRM	3	4.963	5.367	5.241	In vivo splicing in HEK293T and HMVEC using wt or mutated constructs.
HNRNPH3	ENSG00000096746	no	Homo_sapiens	AGGGA	5	11571276	RRM	3	4.963	5.367	5.241	RNA affinity binding assays, immunoblot with WT HeLa nuclear extracts or depleted by RNA affinity chromatography.
HNRNPH3	ENSG00000096746	no	Homo_sapiens	AGGGA	5	17337441	RRM	3	4.963	5.367	5.241	RNA-affinity chromatography assays with HeLa nuclear extract and immunoblotting.

HNRNPH3	ENSG00000096746	no	Homo_sapiens	AGGGA	5	17337441	RRM	3	4.963	5.367	5.241	protein affinity purification
HNRNPH3	ENSG00000096746	yes	Homo_sapiens	AGGGA	5	19404736	RRM	3	4.963	5.367	5.241	Mutational analysis, RNA affinity chromatography, UV cross-linking, immunoprecipitation with HeLa nuclear extract.
HNRNPL	ENSG00000104824	no	Homo_sapiens	AACA	4	15889141	RRM	10	3.489	3.265	3.227	SELEX of 20nt random with recombinant protein. Consensus motif: ACACACAC. Pull-down assays, UV crosslink, immunoprecipitation with HeLa nuclear extracts.
HNRNPL	ENSG00000104824	no	Homo_sapiens	ACAU	4	15889141	RRM	11	3.627	3.654	3.282	SELEX of 20nt random with recombinant protein. Consensus motif: ACACACAC. Pull-down assays, UV crosslink, immunoprecipitation with HeLa nuclear extracts.
HNRNPL	ENSG00000104824	no	Homo_sapiens	UACA	4	15889141	RRM	0	3.844	3.794	3.447	SELEX of 20nt random with recombinant protein. Consensus motif: ACACACAC. Pull-down assays, UV crosslink, immunoprecipitation with HeLa nuclear extracts.
IGF2BP3	ENSG00000136231	no	Homo_sapiens	CAUU	4	20371350	KH;RRM	12	13210	3.775	3.002	PAR-clip
KHDRBS2	ENSG00000112232	no	Homo_sapiens	GAUAAAC	7	23846655	KH	6	10.484	10.392	10.153	RNAcompete
KHDRBS3	ENSG00000131773	no	Homo_sapiens	AUAAAC	6	23846655	KH	7	7.922	8.578	7.414	RNAcompete
KHSRP	ENSG00000088247	no	Homo_sapiens	AGGG	4	18684992	KH	3	3.087	3.548	3.585	NMR
NONO	ENSG00000147140	no	Homo_sapiens	AGGGA	5	9001221	RRM	3	4.963	5.367	5.241	SELEX
PTBP1	ENSG00000011304	no	Homo_sapiens	CUUU	4	16179478	RRM	17	2.805	3.576	2.592	NMR
PTBP1	ENSG00000011304	no	Homo_sapiens	UUACUUU	7	19561594	PTBP1	14	8.591	45200	8.238	RNAcompete using recombinant protein
RBM41	ENSG00000089682	no	Homo_sapiens	UUACUUU	7	23846655	RRM	14	8.591	45200	8.238	RNAcompete
SRSF5	ENSG00000100650	no	Homo_sapiens	ACAGG	5	9649504	RRM	1	5.164	46143	5.218	Functional SELEX of constructs with 20nt random by splicing in HeLa S100 extracts complemented by recombinant protein. Consensus motif: ACDGS. UV crosslink, competition and immunoprecipitation assays in HeLa nuclear extracts.
TIA1	ENSG00000116001	no	Homo_sapiens	CUUUG	5	8576255	RRM	17	4.894	5.086	4.793	SELEX of 68nt random with recombinant protein. Winners confirmed by UV cross-linking and competition assay.
TIAL1	ENSG00000151923	no	Homo_sapiens	AUUA	4	8576255	RRM	13	23071	4.468	43924	SELEX of 68nt random with recombinant protein. Winners confirmed by UV cross-linking and competition assay.
TIAL1	ENSG00000151923	no	Homo_sapiens	CUUUG	5	8576255	RRM	17	4.894	5.086	4.793	SELEX of 68nt random with recombinant protein. Winners confirmed by UV cross-linking and competition assay.
HNRNPH1	ENSG00000169045	no	Homo_sapiens	GGGA	4	11847131;9343212	ZF_RNPHF	4	3.039	3.224	3.386	snRNA inactivation in HeLa cell nuclear extract;Western blot
QKI	ENSG00000112531	no	Homo_sapiens	AUUAAC	6	24722255	KH	3	8.916	9.835	8.452	EMSA with WT and mutated sequences and UV crosslinking with recombinant protein
SF1	ENSG00000168066	no	Homo_sapiens	AUUAAC	6	24722255	KH;ZF_CCHC	3	8.916	9.835	8.452	EMSA with WT and mutated sequences and UV crosslinking with recombinant protein
TIAL1	ENSG00000151923	no	Homo_sapiens	AUUA	4	8576255	RRM	3	4.215	5.053	3.625	SELEX of 68nt random with recombinant protein. Winners confirmed by UV cross-linking and competition assay.

>S6810-6826 apical loop - marked in red

AUGAUUAACAAUAUCAU

Gene name	Gene id	Mutated	Organism	Motif	Length	Reference		Domain	Quality_score			Experiments
HNRNPL	ENSG00000104824	no	Homo_sapiens	AACA	4	15889141	RRM	6	4.074	31107	3.812	SELEX of 20nt random with recombinant protein. Consensus motif: ACACACAC. Pull-down assays, UV crosslink, immunoprecipitation with HeLa nuclear extracts.
HNRNPL	ENSG00000104824	no	Homo_sapiens	ACAA	4	15889141	RRM	7	4.286	3.894	4.007	Pull-down assays, UV crosslink, immunoprecipitation with HeLa nuclear extracts.
NOVA1	ENSG00000139910	no	Homo_sapiens	UCAU	4	10811881	KH	13	4.058	4.159	3.788	SELEX
NOVA1	ENSG00000139910	no	Homo_sapiens	UCAU	4	17655233	KH	13	4.058	4.159	3.788	NMR
NOVA2	ENSG00000104967	no	Homo_sapiens	UCAU	4	10811881	KH	13	4.058	4.159	3.788	SELEX
QKI	ENSG00000112531	no	Homo_sapiens	AUUAAC	6	24722255	KH	3	8.916	9.835	8.452	EMSA with WT and mutated sequences and UV crosslinking with recombinant protein
SF1	ENSG00000168066	no	Homo_sapiens	AUUAAC	6	24722255	KH;ZF_CCHC	3	8.916	9.835	8.452	EMSA with WT and mutated sequences and UV crosslinking with recombinant protein
TIAL1	ENSG00000151923	no	Homo_sapiens	AUUA	4	8576255	RRM	3	4.215	5.053	3.625	SELEX of 68nt random with recombinant protein. Winners confirmed by UV cross-linking and competition assay.

>SL6854-6878 apical loop - marked in red

CAAAGGGAUUGACUUGGACCAAUUC

Gene name	Gene id	Mutated	Organism	Motif	Length	Reference		Domain	Quality_score			Experiments
EIF4B	ENSG00000063046	no	Homo_sapiens	CUUGGAC	7	8846295	RRM	12	9.817	9.354	10.161	SELEX
ESRP1	ENSG00000104413	no	Homo_sapiens	AGGGAU	6	20526337	RRM	3	7.588	8.033	21002	NMR spectroscopy
GRSF1	ENSG00000132463	no	Homo_sapiens	AGGGA	5	17337441	RRM	3	4.892	5.297	5.171	protein affinity purification
HNRNPF	ENSG00000169813	no	Homo_sapiens	AGGGA	5	11571276	RRM;ZF_RNPHF	3	4.892	5.297	5.171	RNA affinity binding assays, immunoblot with WT HeLa nuclear extracts or depleted by RNA affinity chromatography.
HNRNPF	ENSG00000169813	no	Homo_sapiens	AGGGA	5	17337441	RRM;ZF_RNPHF	3	4.892	5.297	5.171	RNA-affinity chromatography assays with HeLa nuclear extract and immunoblotting.
HNRNPF	ENSG00000169813	no	Homo_sapiens	AGGGAU	6	20526337	RRM;ZF_RNPHF	3	7.588	8.033	21002	NMR spectroscopy
HNRNPF	ENSG00000169813	no	Homo_sapiens	AGGGAU	6	23275549	RRM;ZF_RNPHF	3	7.588	8.033	21002	NMR spectroscopy, isothermal titration calorimetry
HNRNPF	ENSG00000169813	no	Homo_sapiens	AGGGA	5	17337441	RRM;ZF_RNPHF	3	4.892	5.297	5.171	protein affinity purification
HNRNPF	ENSG00000169813	yes	Homo_sapiens	AGGGA	5	19404736	RRM;ZF_RNPHF	3	4.892	5.297	5.171	Mutational analysis, RNA affinity chromatography, UV cross-linking, immunoprecipitation with HeLa nuclear extract.
HNRNPH1	ENSG00000169045	no	Homo_sapiens	AGGGA	5	20028692	ZF_RNPHF	3	4.892	5.297	5.171	In vivo splicing in HEK293T and HMVEC using wt or mutated constructs.
HNRNPH1	ENSG00000169045	no	Homo_sapiens	AGGGA	5	11571276	ZF_RNPHF	3	4.892	5.297	5.171	RNA affinity binding assays, immunoblot with WT HeLa nuclear extracts or depleted by RNA affinity chromatography.

HNRNPH1	ENSG00000169045	no	Homo_sapiens	AGGGA	5	17337441	ZF_RNPHF	3	4.892	5.297	5.171	RNA-affinity chromatography assays with HeLa nuclear extract and immunoblotting.
HNRNPH1	ENSG00000169045	no	Homo_sapiens	AGGGA	5	17337441	ZF_RNPHF	3	4.892	5.297	5.171	protein affinity purification
HNRNPH1	ENSG00000169045	yes	Homo_sapiens	AGGGA	5	19404736	ZF_RNPHF	3	4.892	5.297	5.171	Mutational analysis, RNA affinity chromatography, UV cross-linking, immunoprecipitation with HeLa nuclear extract.
HNRNPH2	ENSG00000126945	no	Homo_sapiens	AGGGA	5	20028692	ZF_RNPHF	3	4.892	5.297	5.171	In vivo splicing in HEK293T and HMVEC using wt or mutated constructs.
HNRNPH2	ENSG00000126945	no	Homo_sapiens	AGGGA	5	11571276	ZF_RNPHF	3	4.892	5.297	5.171	RNA affinity binding assays, immunoblot with WT HeLa nuclear extracts or depleted by RNA affinity chromatography.
HNRNPH2	ENSG00000126945	no	Homo_sapiens	AGGGA	5	17337441	ZF_RNPHF	3	4.892	5.297	5.171	RNA-affinity chromatography assays with HeLa nuclear extract and immunoblotting.
HNRNPH2	ENSG00000126945	no	Homo_sapiens	AGGGA	5	17337441	ZF_RNPHF	3	4.892	5.297	5.171	protein affinity purification
HNRNPH2	ENSG00000126945	yes	Homo_sapiens	AGGGA	5	19404736	ZF_RNPHF	3	4.892	5.297	5.171	Mutational analysis, RNA affinity chromatography, UV cross-linking, immunoprecipitation with HeLa nuclear extract.
HNRNPH3	ENSG00000096746	no	Homo_sapiens	AGGGA	5	20028692	RRM	3	4.892	5.297	5.171	In vivo splicing in HEK293T and HMVEC using wt or mutated constructs.
HNRNPH3	ENSG00000096746	no	Homo_sapiens	AGGGA	5	11571276	RRM	3	4.892	5.297	5.171	RNA affinity binding assays, immunoblot with WT HeLa nuclear extracts or depleted by RNA affinity chromatography.
HNRNPH3	ENSG00000096746	no	Homo_sapiens	AGGGA	5	17337441	RRM	3	4.892	5.297	5.171	RNA-affinity chromatography assays with HeLa nuclear extract and immunoblotting.
HNRNPH3	ENSG00000096746	no	Homo_sapiens	AGGGA	5	17337441	RRM	3	4.892	5.297	5.171	protein affinity purification
HNRNPH3	ENSG00000096746	yes	Homo_sapiens	AGGGA	5	19404736	RRM	3	4.892	5.297	5.171	Mutational analysis, RNA affinity chromatography, UV cross-linking, immunoprecipitation with HeLa nuclear extract.
KHSRP	ENSG00000088247	no	Homo_sapiens	AGGG	4	18684992	KH	3	43864	3.481	3.518	NMR
NONO	ENSG00000147140	no	Homo_sapiens	AGGGA	5	9001221	RRM	3	4.892	5.297	5.171	SELEX
OAS1	ENSG00000089127	no	Homo_sapiens	UUGA	4	23319625	OAS1_C;NTP_TRANSF_2	8	13940	3.593	3.037	X-RAY DIFFRACTION
PTBP1	ENSG00000011304	no	Homo_sapiens	CUUG	4	16179478	RRM	12	3.345	3.716	3.267	NMR
SRSF10	ENSG00000188529	no	Homo_sapiens	AAAGGGA	7	23846655	RRM	1	8.893	8.967	8.777	RNAcompete
SRSF5	ENSG00000100650	no	Homo_sapiens	AAAGG	5	9649504	RRM	1	5.123	5.051	5.118	Functional SELEX of constructs with 20nt random by splicing in HeLa S100 extracts complemented by recombinant protein. Consensus motif: ACDGS. UV crosslink, competition and immunoprecipitation assays in HeLa nuclear extracts.
SRSF9	ENSG00000111786	no	Homo_sapiens	UGGAC	5	17548433	RRM	14	5.605	4.777	6.177	SELEX of 20nt random with recombinant protein. Consensus motif: AGGAC. EMSA, UV crosslink, SDS-PAGE with HeLa nuclear extracts.
TIA1	ENSG00000116001	no	Homo_sapiens	AUUC	4	8576255	RRM	21	3.681	3.902	3.349	SELEX of 68nt random with recombinant protein. Winners confirmed by UV cross-linking and competition assay.
TIAL1	ENSG00000151923	no	Homo_sapiens	AUUC	4	8576255	RRM	21	3.681	3.902	3.349	SELEX of 68nt random with recombinant protein. Winners confirmed by UV cross-linking and competition assay.
HNRNPH1	ENSG00000169045	no	Homo_sapiens	GGGA	4	11847131;9343212	ZF_RNPHF	4	2.972	3.157	3.319	snRNA inactivation in HeLa cell nuclear extract;Western blot

>SL6891-6922 apical loop - marked in red

UAUGGUGAUGAUGUGAUCGCAUCGUACCCAUG

Gene name	Gene id	Mutated	Organism	Motif	Length	Reference		Domain	Quality_score			Experiments
CELF2	ENSG00000048740	no	Homo_sapiens	AUGUG	5	15657417	RRM	10	4.758	4.781	22737	SELEX of 20nt random with recombinant protein, some winner sequences are verified by EMSA.
CELF2	ENSG00000048740	no	Homo_sapiens	CAUCG	5	15657417	RRM	19	6.944	5.605	8.185	SELEX of 20nt random with recombinant protein, some winner sequences are verified by EMSA.
CMTR1	ENSG00000137200	no	Homo_sapiens	GAUC	4	24402442	FTSJ;G-PATCH	14	3.859	3.411	3.774	X-RAY DIFFRACTION
FUS	ENSG00000089280	no	Homo_sapiens	GGUG	4	11098054	ZF_RANBP;RRM	3	2.656	2.759	3.049	SELEX
FUS	ENSG00000089280	no	Homo_sapiens	UGGUGA	6	11098054	RRM	2	6.401	6.204	6.523	SELEX of 25nt random with recombinant protein. Consensus motif: GGUG. SDS-PAGE, EMSA, UV crosslink, competition and immunoprecipitation assays with HeLa nuclear extracts.
HNRNPK	ENSG00000165119	no	Homo_sapiens	ACCCAU	6	11278705	KH	25	7.611	7.606	7.539	SELEX of 50nt random with recombinant protein. Consensus motif: u/aCCCCu/a. Winners confirmed by EMSA with recombinant protein.
NOVA1	ENSG00000139910	no	Homo_sapiens	CCAU	4	17655233	KH	27	3.112	2.984	3.011	NMR
PHAX	ENSG00000164902	no	Homo_sapiens	AUCG	4	20430857	RNA_GG_BIND	15;20	6.524	18019	7.089	SOLUTION NMR
RBM46	ENSG00000151962	no	Homo_sapiens	GAUGAUG	7	23846655	RRM	6	8.478	7.277	8.808	RNAcompete
SRSF1	ENSG00000136450	no	Homo_sapiens	UGAUGAU	7	18841201	RRM	5	8.586	7.803	8.531	genome-wide in vivo immunoprecipitation
SRSF1	ENSG00000136450	no	Homo_sapiens	UGGUGAU	7	18841201	RRM	2	8.842	8.381	8.718	genome-wide in vivo immunoprecipitation
SRSF3	ENSG00000112081	no	Homo_sapiens	CAUC	4	17036044	RRM	19	3.192	2.646	3.354	NMR spectroscopy
SRSF3	ENSG00000112081	no	Homo_sapiens	GAUC	4	17036044	RRM	14	3.859	3.411	3.774	NMR spectroscopy
SRSF6	ENSG00000124193	no	Homo_sapiens	AUCGUA	6	9649504	RRM	20	10.286	10.013	10.219	Functional SELEX of constructs with 20nt random by splicing in HeLa S100 extracts complemented by recombinant protein. Consensus motif: USCGKM. UV crosslink, competition and immunoprecipitation assays in HeLa nuclear extracts.
YBX1	ENSG00000065978	no	Homo_sapiens	CAUC	4	22730292	CSD	19	3.192	2.646	3.354	In vivo splicing in HEK-293 cells with wt and mutated sequences, EMSA
YBX1	ENSG00000065978	no	Homo_sapiens	CAUC	4	22730292	CSD	19	3.192	2.646	3.354	SELEX of 20nt random sequences with recombinant protein. Winners confirmed by EMSA with recombinant protein.
YBX1	ENSG00000065978	no	Homo_sapiens	GAUC	4	22730292	CSD	14	3.859	3.411	3.774	EMSA with recombinant protein.
YTHDC1	ENSG00000083896	no	Homo_sapiens	UCGUAC	6	20167602	YTH	21	10.717	10.015	11.089	SELEX
SRSF5	ENSG00000100650	no	Homo_sapiens	CGCAUC	6	11967553;12824367	RRM	17	41518	7.961	10.481	SELEX

>SL6940-6957 apical loop - marked in red

UACUCGCUGAAGCUGGUA

Gene name	Gene id	Mutated	Organism	Motif	Length	Reference	Domain		Quality_score			Experiments
F2	ENSG00000180210	no	Homo_sapiens	GAAG	4	18971322	GLA;KRINGLE;THROMBIN_L	8	3.591	2.922	3.915	X-RAY DIFFRACTION
MBNL1	ENSG00000152601	no	Homo_sapiens	UCGCUG	6	20071745	ZF_CCCH	3	9.456	26512	10.566	SELEX of 32nt random with recombinant protein. Consensus motifs: YGCY, YYYGCUKY.
MBNL1	ENSG00000152601	no	Homo_sapiens	CGCU	4	20071745	ZF_CCCH	4	5.552	4.951	6.706	SELEX
PTBP1	ENSG00000011304	no	Homo_sapiens	CUGA	4	16179478	RRM	6	3.615	3.448	3.742	NMR
PTBP1	ENSG00000011304	no	Homo_sapiens	CUCG	4	16179478	RRM	2	5.569	5.186	44713	NMR
PTBP1	ENSG00000011304	no	Homo_sapiens	CUGG	4	16179478	RRM	12	3.121	2.881	3.492	NMR
SAMD4A	ENSG00000020577	no	Homo_sapiens	GCUGGUA	7	23846655	SAM	11	10.713	27303	10.847	RNAcompete
SRSF1	ENSG00000136450	no	Homo_sapiens	UGAAGCU	7	18841201	RRM	7	9.708	8.949	9.969	genome-wide in vivo immunoprecipitation

>SL6987-7036 apical loop - marked in red

GAUAAGGGAGAGUGCUUUAACGAAGUUACCUGGACCAACGCCACUUUCCU

Gene name	Gene id	Mutated	Organism	Motif	Length	Reference	Domain		Quality_score			Experiments
CPEB4	ENSG00000113742	no	Homo_sapiens	CUUUA	5	24990967	RRM	14	4.206	5.066	3.833	SOLUTION NMR
ELAVL2	ENSG00000107105	no	Homo_sapiens	CUUUA	5	8497264	RRM	14	4.206	5.066	3.833	SELEX of 25nt random with recombinant protein.
ELAVL2	ENSG00000107105	no	Homo_sapiens	CUUUC	5	8497264	RRM	43	3.749	4.502	3.714	SELEX of 25nt random with recombinant protein.
F2	ENSG00000180210	no	Homo_sapiens	GAAG	4	18971322	GLA;KRINGLE;THROMBIN_L	21	1.944	1.274	2.267	X-RAY DIFFRACTION
GRSF1	ENSG00000132463	no	Homo_sapiens	AGGGA	5	17337441	RRM	4	3.761	4.166	43925	protein affinity purification
HNRNPF	ENSG00000169813	no	Homo_sapiens	AGGGA	5	11571276	RRM;ZF_RNPHF	4	3.761	4.166	43925	RNA affinity binding assays, immunoblot with WT HeLa nuclear extracts or depleted by RNA affinity chromatography.
HNRNPF	ENSG00000169813	no	Homo_sapiens	AGGGA	5	17337441	RRM;ZF_RNPHF	4	3.761	4.166	43925	RNA-affinity chromatography assays with HeLa nuclear extract and immunoblotting.
HNRNPF	ENSG00000169813	no	Homo_sapiens	AGGGA	5	17337441	RRM;ZF_RNPHF	4	3.761	4.166	43925	protein affinity purification
HNRNPF	ENSG00000169813	yes	Homo_sapiens	AGGGA	5	19404736	RRM;ZF_RNPHF	4	3.761	4.166	43925	Mutational analysis, RNA affinity chromatography, UV cross-linking, immunoprecipitation with HeLa nuclear extract.
HNRNPH1	ENSG00000169045	no	Homo_sapiens	AGGGA	5	20028692	ZF_RNPHF	4	3.761	4.166	43925	In vivo splicing in HEK293T and HMVEC using wt or mutated constructs.
HNRNPH1	ENSG00000169045	no	Homo_sapiens	AGGGA	5	11571276	ZF_RNPHF	4	3.761	4.166	43925	RNA affinity binding assays, immunoblot with WT HeLa nuclear extracts or depleted by RNA affinity chromatography.
HNRNPH1	ENSG00000169045	no	Homo_sapiens	AGGGA	5	17337441	ZF_RNPHF	4	3.761	4.166	43925	RNA-affinity chromatography assays with HeLa nuclear extract and immunoblotting.
HNRNPH1	ENSG00000169045	no	Homo_sapiens	AGGGA	5	17337441	ZF_RNPHF	4	3.761	4.166	43925	protein affinity purification
HNRNPH1	ENSG00000169045	yes	Homo_sapiens	AGGGA	5	19404736	ZF_RNPHF	4	3.761	4.166	43925	Mutational analysis, RNA affinity chromatography, UV cross-linking, immunoprecipitation with HeLa nuclear extract.
HNRNPH2	ENSG00000126945	no	Homo_sapiens	AGGGA	5	20028692	ZF_RNPHF	4	3.761	4.166	43925	In vivo splicing in HEK293T and HMVEC using wt or mutated constructs.
HNRNPH2	ENSG00000126945	no	Homo_sapiens	AGGGA	5	11571276	ZF_RNPHF	4	3.761	4.166	43925	RNA affinity binding assays, immunoblot with WT HeLa nuclear extracts or depleted by RNA affinity chromatography.
HNRNPH2	ENSG00000126945	no	Homo_sapiens	AGGGA	5	17337441	ZF_RNPHF	4	3.761	4.166	43925	RNA-affinity chromatography assays with HeLa nuclear extract and immunoblotting.
HNRNPH2	ENSG00000126945	no	Homo_sapiens	AGGGA	5	17337441	ZF_RNPHF	4	3.761	4.166	43925	protein affinity purification
HNRNPH2	ENSG00000126945	yes	Homo_sapiens	AGGGA	5	19404736	ZF_RNPHF	4	3.761	4.166	43925	Mutational analysis, RNA affinity chromatography, UV cross-linking, immunoprecipitation with HeLa nuclear extract.
HNRNPH3	ENSG00000096746	no	Homo_sapiens	AGGGA	5	20028692	RRM	4	3.761	4.166	43925	In vivo splicing in HEK293T and HMVEC using wt or mutated constructs.
HNRNPH3	ENSG00000096746	no	Homo_sapiens	AGGGA	5	11571276	RRM	4	3.761	4.166	43925	RNA affinity binding assays, immunoblot with WT HeLa nuclear extracts or depleted by RNA affinity chromatography.
HNRNPH3	ENSG00000096746	no	Homo_sapiens	AGGGA	5	17337441	RRM	4	3.761	4.166	43925	RNA-affinity chromatography assays with HeLa nuclear extract and immunoblotting.
HNRNPH3	ENSG00000096746	no	Homo_sapiens	AGGGA	5	17337441	RRM	4	3.761	4.166	43925	protein affinity purification
HNRNPH3	ENSG00000096746	yes	Homo_sapiens	AGGGA	5	19404736	RRM	4	3.761	4.166	43925	Mutational analysis, RNA affinity chromatography, UV cross-linking, immunoprecipitation with HeLa nuclear extract.
HNRNPL	ENSG00000104824	no	Homo_sapiens	CCAC	4	15889141	RRM	40	2.203	1.952	2.458	SELEX of 20nt random with recombinant protein. Consensus motif: ACACACAC. Pull-down assays, UV crosslink, immunoprecipitation with HeLa nuclear extracts.
KHSRP	ENSG00000088247	no	Homo_sapiens	AGGG	4	18684992	KH	4	1.925	2.386	2.423	NMR
MBNL1	ENSG00000152601	no	Homo_sapiens	GUGCUU	6	20071745	ZF_CCCH	11	6.279	6.807	11475	SELEX of 32nt random with recombinant protein. Consensus motifs: YGCY, YYYGCUKY.
MBNL1	ENSG00000152601	no	Homo_sapiens	CGCC	4	20071745	ZF_CCCH	38	3.245	28522	4.379	SELEX
MBNL1	ENSG00000152601	no	Homo_sapiens	UGCU	4	20071745	ZF_CCCH	12	1.947	1.845	2.053	SELEX
NONO	ENSG00000147140	no	Homo_sapiens	AGGGA	5	9001221	RRM	4	3.761	4.166	43925	SELEX
NOVA1	ENSG00000139910	no	Homo_sapiens	CCAC	4	17655233	KH	40	2.203	1.952	2.458	NMR
NXF1	ENSG00000162231	no	Homo_sapiens	CCUG	4	21822283	LRR_1;NTF2;TAP-RNA_BINE	28	1.418	1.229	1.836	X-RAY DIFFRACTION
PIWIL1	ENSG00000125207	no	Homo_sapiens	GCUU	4	21193640	GAGE;PAZ;PIWI	13	2.401	2.605	2.425	X-RAY DIFFRACTION
PTBP1	ENSG00000011304	no	Homo_sapiens	CUUU	4	16179478	RRM	14;43	2.643	3.413	15738	NMR
PTBP1	ENSG00000011304	no	Homo_sapiens	CUGG	4	16179478	RRM	29	1.474	1.233	1.844	NMR
PTBP1	ENSG00000011304	no	Homo_sapiens	CCUGG	5	16179478	RRM	28	3.025	2.761	3.536	NMR
RC3H1	ENSG00000135870	no	Homo_sapiens	UUUC	4	25026078	ZF_CCCH	44	1.674	2.634	1.434	X-RAY DIFFRACTION

SRP68	ENSG00000167881	no	Homo_sapiens	UAAG	4	24700861	SRP68	2	2.756	3.674	2.544	X-RAY DIFFRACTION
SRSF1	ENSG00000136450	no	Homo_sapiens	CCAACGC	7	16825284	RRM	34	10.381	9.214	12.005	Functional SELEX of random 7nt and 14nt with recombinant protein. Consensus motifs: CSScSSR and CSMMSSA.
SRSF2	ENSG00000161547	no	Homo_sapiens	GGAGAG	6	23846655	RRM	6	11444	5.064	5.866	RNAcompete
SRSF9	ENSG00000111786	no	Homo_sapiens	UGGAC	5	17548433	RRM	30	4.474	3.646	5.046	SELEX of 20nt random with recombinant protein. Consensus motif: AGGAC. EMSA, UV crosslink, SDS-PAGE with HeLa nuclear extracts.
TIA1	ENSG00000116001	no	Homo_sapiens	CUUUA	5	8576255	RRM	14	4.206	5.066	3.833	SELEX of 68nt random with recombinant protein. Winners confirmed by UV cross-linking and competition assay.
TIA1	ENSG00000116001	no	Homo_sapiens	CUUUC	5	8576255	RRM	43	3.749	4.502	3.714	SELEX of 68nt random with recombinant protein. Winners confirmed by UV cross-linking and competition assay.
TIAL1	ENSG00000151923	no	Homo_sapiens	CUUUA	5	8576255	RRM	14	4.206	5.066	3.833	SELEX of 68nt random with recombinant protein. Winners confirmed by UV cross-linking and competition assay.
TIAL1	ENSG00000151923	no	Homo_sapiens	CUUUC	5	8576255	RRM	43	3.749	4.502	3.714	SELEX of 68nt random with recombinant protein. Winners confirmed by UV cross-linking and competition assay.
YTHDC1	ENSG00000083896	no	Homo_sapiens	GAGUGC	6	20167602	YTH	9	6.362	6.383	6.171	SELEX
ZFP36	ENSG00000128016	no	Homo_sapiens	ACUUUC	6	18367721	ZF_CCCH	42	45444	6.672	6.084	RIP-chip
RBMX	ENSG00000147274	no	Homo_sapiens	AAGU	4	12761049	RRM	22	2.322	2.427	2.117	Mutagenesis;Deletion analysis
HNRNPH1	ENSG00000169045	no	Homo_sapiens	GGGA	4	11847131;9343212	ZF_RNPHF	5	1.877	2.062	2.224	snRNA inactivation in HeLa cell nuclear extract;Western blot

>SL7159-7184 apical loop - marked in red

GCUCACUGUGUCUAUUAGCUUGGCAUAACGGGGAGC

Gene name	Gene id	Mutated	Organism	Motif	Length	Reference	Domain		Quality_score			Experiments
CELF1	ENSG00000149187	no	Homo_sapiens	UGUGU	5	16938098	RRM	6	3.975	4.927	3.822	SELEX of 35nt random with recombinant protein. Surface plasmon resonance and EMSA.
CELF2	ENSG00000048740	no	Homo_sapiens	UGUGU	5	15657417	RRM	6	3.975	4.927	3.822	SELEX of 20nt random with recombinant protein, some winner sequences are verified by EMSA.
CELF2	ENSG00000048740	no	Homo_sapiens	UGUGU	5	11931771	RRM	6	3.975	4.927	3.822	UV crosslink, western blot, immunoprecipitation in HeLa nuclear extracts.
GRSF1	ENSG00000132463	no	Homo_sapiens	GGGGA	5	17337441	RRM	29	44652	4.502	4.937	protein affinity purification
HNRNPF	ENSG00000169813	no	Homo_sapiens	GGGGA	5	17337441	RRM;ZF_RNPHF	29	44652	4.502	4.937	protein affinity purification
HNRNPF	ENSG00000169813	no	Homo_sapiens	GGGGA	5	16885237	RRM;ZF_RNPHF	29	44652	4.502	4.937	NMR titration using recombinant protein
HNRNPF	ENSG00000169813	no	Homo_sapiens	GGGGA	5	11571276	RRM;ZF_RNPHF	29	44652	4.502	4.937	RNA affinity binding assays, immunoblot with WT HeLa nuclear extracts or depleted by RNA affinity chromatography.
HNRNPH1	ENSG00000169045	no	Homo_sapiens	GGGGA	5	17337441	ZF_RNPHF	29	44652	4.502	4.937	protein affinity purification
HNRNPH1	ENSG00000169045	no	Homo_sapiens	GGGGA	5	20028692	ZF_RNPHF	29	44652	4.502	4.937	EMSA, UV cross-linking, immunoprecipitation and Western blotting with HEK293T nuclear extract
HNRNPH1	ENSG00000169045	no	Homo_sapiens	GGGGA	5	11571276	ZF_RNPHF	29	44652	4.502	4.937	RNA affinity binding assays, immunoblot with WT HeLa nuclear extracts or depleted by RNA affinity chromatography.
HNRNPH2	ENSG00000126945	no	Homo_sapiens	GGGGA	5	17337441	ZF_RNPHF	29	44652	4.502	4.937	protein affinity purification
HNRNPH2	ENSG00000126945	no	Homo_sapiens	GGGGA	5	20028692	ZF_RNPHF	29	44652	4.502	4.937	EMSA, UV cross-linking, immunoprecipitation and Western blotting with HEK293T nuclear extract
HNRNPH2	ENSG00000126945	no	Homo_sapiens	GGGGA	5	11571276	ZF_RNPHF	29	44652	4.502	4.937	RNA affinity binding assays, immunoblot with WT HeLa nuclear extracts or depleted by RNA affinity chromatography.
HNRNPH3	ENSG00000096746	no	Homo_sapiens	GGGGA	5	17337441	RRM	29	44652	4.502	4.937	protein affinity purification
HNRNPH3	ENSG00000096746	no	Homo_sapiens	GGGGA	5	20028692	RRM	29	44652	4.502	4.937	EMSA, UV cross-linking, immunoprecipitation and Western blotting with HEK293T nuclear extract
HNRNPH3	ENSG00000096746	no	Homo_sapiens	GGGGA	5	11571276	RRM	29	44652	4.502	4.937	RNA affinity binding assays, immunoblot with WT HeLa nuclear extracts or depleted by RNA affinity chromatography.
KHSRP	ENSG00000088247	no	Homo_sapiens	UUAG	4	18684992	KH	14	3.305	4.513	29983	NMR
NOVA1	ENSG00000139910	no	Homo_sapiens	UCAC	4	10811881	KH	2	3.035	3.033	2.907	SELEX
NOVA1	ENSG00000139910	no	Homo_sapiens	UGUGU	5	17655233	KH	6	3.975	4.927	3.822	NMR titrations using recombinant protein
NOVA1	ENSG00000139910	no	Homo_sapiens	UCAC	4	17655233	KH	2	3.035	3.033	2.907	NMR
NOVA2	ENSG00000104967	no	Homo_sapiens	UCAC	4	10811881	KH	2	3.035	3.033	2.907	SELEX
OAS1	ENSG00000089127	no	Homo_sapiens	GCAUAA	6	23319625	OAS1_C;NTP_TRANSF_2	22	7.949	8.637	7.416	X-RAY DIFFRACTION
PIWIL1	ENSG00000125207	no	Homo_sapiens	GCUU	4	21193640	GAGE;PAZ;PIWI	17	2.911	3.115	2.936	X-RAY DIFFRACTION
PTBP1	ENSG00000011304	no	Homo_sapiens	CUCA	4	16179478	RRM	1	2.569	2.544	43953	NMR
PTBP1	ENSG00000011304	no	Homo_sapiens	CUAU	4	16179478	RRM	11	3.568	3.666	3.185	NMR
PTBP1	ENSG00000011304	no	Homo_sapiens	CUGU	4	16179478	RRM	5	2.346	2.568	2.373	NMR
PTBP1	ENSG00000011304	no	Homo_sapiens	CUUG	4	16179478	RRM	18	27791	3.131	2.682	NMR
PTBP1	ENSG00000011304	no	Homo_sapiens	UCUAU	5	16179478	RRM	10	5.321	5.712	4.826	NMR
SRP19	ENSG00000153037	no	Homo_sapiens	GAGC	4	11641499	SRP19	32	2.775	2.336	3.283	X-RAY DIFFRACTION
SRP19	ENSG00000153037	no	Homo_sapiens	GGAGC	5	20179341	SRP19	31	4.362	3.741	12905	X-RAY DIFFRACTION
SRP54	ENSG00000100883	no	Homo_sapiens	GGAGC	5	12244299	SRP54,SRP_SPB	31	4.362	3.741	12905	X-RAY DIFFRACTION
SRP68	ENSG00000167881	no	Homo_sapiens	GGAGC	5	24700861	SRP68	31	4.362	3.741	12905	X-RAY DIFFRACTION

SRSF5	ENSG00000100650	no	Homo_sapiens	ACGGG	5	9649504	RRM	27	6.383	5.835	7.289	Functional SELEX of constructs with 20nt random by splicing in HeLa S100 extracts complemented by recombinant protein. Consensus motif: ACDGS. UV crosslink, competition and immunoprecipitation assays in HeLa nuclear extracts.
TIAL1	ENSG00000151923	no	Homo_sapiens	AUUA	4	8576255	RRM	13	2.978	3.816	2.388	SELEX of 68nt random with recombinant protein. Winners confirmed by UV cross-linking and competition assay.
SRSF2	ENSG00000161547	no	Homo_sapiens	UUAG	4	10022872;9710624;762	RRM	14	3.305	4.513	29983	In vitro splicing assays and RNA competition assays
HNRNPA1	ENSG00000135486	no	Homo_sapiens	UUAG	4	10022872;9710624;762	RRM	14	3.305	4.513	29983	In vitro splicing assays and RNA competition assays
HNRNPH1	ENSG00000169045	no	Homo_sapiens	GGGG	4	15506926	ZF_RNPHF	29	2.281	2.781	3.182	Immunoprecipitation;UV cross-linking
HNRNPH1	ENSG00000169045	no	Homo_sapiens	GGGA	4	11847131;9343212	ZF_RNPHF	30	2.387	2.572	2.734	snRNA inactivation in HeLa cell nuclear extract;Western blot
NOVA1	ENSG00000139910	no	Homo_sapiens	UCAC	4	15367696	KH	2	3.035	3.033	2.907	RNA affinity binding assays

>SL7247-7291

UUUGACCCUCCCCGCGUUUUCAACUCUACGCAGGAAGUGGUUGGA

Gene name	Gene id	Mutated	Organism	Motif	Length	Reference	Domain		Quality_score			Experiments
AGO1	ENSG00000092847	no	Homo_sapiens	ACUCU	5	15152257	PAZ;PIWI	22	4.581	4.823	16893	X-RAY DIFFRACTION
DHX9	ENSG00000135829	no	Homo_sapiens	CGCG	4	23361462	DEAD;DSRM;HA2;HELICASE	12	4.393	45017	6.511	X-RAY DIFFRACTION
ELAVL1	ENSG00000066044	no	Homo_sapiens	UUUU	4	23519412	RRM	16	0.925	2.779	0.388	X-RAY DIFFRACTION
ELAVL2	ENSG00000107105	no	Homo_sapiens	GUUUUC	6	8497264	RRM	15	5.862	7.073	5.604	SELEX of 25nt random with recombinant protein.
F2	ENSG00000180210	no	Homo_sapiens	GAAG	4	18971322	GLA;KRINGLE;THROMBIN_L	33	2.106	1.437	15738	X-RAY DIFFRACTION
OAS1	ENSG00000089127	no	Homo_sapiens	UUGA	4	23319625	OAS1_C;NTP_TRANSF_2	1	2.447	24139	2.104	X-RAY DIFFRACTION
PCBP1	ENSG00000169564	no	Homo_sapiens	CCCUCCC	7	12011088	KH	5	6.418	23924	7.307	Mutational analysis, UV cross-linking, Immunoprecipitation, competition assay in LNCaP extracts
PCBP2	ENSG00000197111	no	Homo_sapiens	CCCU	4	17526645	KH	5	1.944	2.121	2.579	X-RAY DIFFRACTION
PCBP2	ENSG00000197111	no	Homo_sapiens	CCCUCCC	7	12011088	KH	5	6.418	23924	7.307	Mutational analysis, UV cross-linking, Immunoprecipitation, competition assay in LNCaP extracts
PCBP2	ENSG00000197111	no	Homo_sapiens	CCUCCCC	7	23846655	KH	6	6.529	7.351	7.511	RNAcompete
PCBP2	ENSG00000197111	no	Homo_sapiens	CCCUCCC	7	23846655	KH	5	6.418	23924	7.307	RNAcompete
PTBP1	ENSG00000011304	no	Homo_sapiens	CUCU	4	16179478	RRM	23	1.934	2.321	2.053	SOLUTION NMR
PTBP1	ENSG00000011304	no	Homo_sapiens	CUCU	4	16179478	RRM	23	1.934	2.321	2.053	NMR
PTBP1	ENSG00000011304	no	Homo_sapiens	CUAC	4	16179478	RRM	25	3.294	2.735	3.283	NMR
PTBP1	ENSG00000011304	no	Homo_sapiens	CUCC	4	16179478	RRM	7	1.914	1.989	2.244	NMR
PTBP1	ENSG00000011304	no	Homo_sapiens	UCUAC	5	16179478	RRM	24	5.113	4.709	4.931	NMR
PTBP1	ENSG00000011304	no	Homo_sapiens	CCUCC	5	16179478	RRM	6	3.391	3.583	26724	NMR
RC3H1	ENSG00000135870	no	Homo_sapiens	UUUC	4	25026078	ZF_CCCH	17	1.836	2.797	1.596	X-RAY DIFFRACTION
SFPQ	ENSG00000116560	no	Homo_sapiens	GUGGUUG	7	23846655	RRM	36	8.579	8.694	8.658	RNAcompete
SRP14	ENSG00000140319	no	Homo_sapiens	GUGG	4	11089964	SRP14	36	2.054	1.918	2.415	X-RAY DIFFRACTION
SRP19	ENSG00000153037	no	Homo_sapiens	CCUC	4	20179341	SRP19	6	1.914	1.993	2.248	X-RAY DIFFRACTION
SRP54	ENSG00000100883	no	Homo_sapiens	CCUC	4	12244299	SRP54,SRP_SPB	6	1.914	1.993	2.248	X-RAY DIFFRACTION
SRP68	ENSG00000167881	no	Homo_sapiens	CCUC	4	24700861	SRP68	6	1.914	1.993	2.248	X-RAY DIFFRACTION
SRSF1	ENSG00000136450	no	Homo_sapiens	CGCAGGA	7	16825284	RRM	28	9.471	30895	11.156	SELEX
SRSF1	ENSG00000136450	no	Homo_sapiens	CGCAGGA	7	16825284	RRM	28	9.471	30895	11.156	SELEX
SRSF1	ENSG00000136450	no	Homo_sapiens	CCCCGCG	7	16825284	RRM	9	8.856	33817	11.683	SELEX
SRSF5	ENSG00000100650	no	Homo_sapiens	GCAGG	5	9649504	RRM	29	3.679	3.701	4.339	Functional SELEX of constructs with 20nt random by splicing in HeLa S100 extracts complemented by recombinant protein. Consensus motif: ACDGS. UV crosslink, competition and immunoprecipitation assays in HeLa nuclear extracts.
SRSF9	ENSG00000111786	no	Homo_sapiens	AGGAA	5	17548433	RRM	31	26359	3.415	3.764	SELEX of 20nt random with recombinant protein. Consensus motif: AGGAC. EMSA, UV crosslink, SDS-PAGE with HeLa nuclear extracts.
SSB	ENSG00000138385	no	Homo_sapiens	UUUU	4	18547518	LA;RRM	16	0.925	2.779	0.388	X-RAY DIFFRACTION
TIA1	ENSG00000116001	no	Homo_sapiens	GUUUUC	6	8576255	RRM	15	5.862	7.073	5.604	SELEX of 68nt random with recombinant protein. Winners confirmed by UV cross-linking and competition assay.
TIAL1	ENSG00000151923	no	Homo_sapiens	GUUUUC	6	8576255	RRM	15	5.862	7.073	5.604	SELEX of 68nt random with recombinant protein. Winners confirmed by UV cross-linking and competition assay.
TRA2B	ENSG00000136527	no	Homo_sapiens	UCAAC	5	20926394	RRM	19	5.221	4.469	5.241	NMR spectroscopy
XPO5	ENSG00000124571	no	Homo_sapiens	CCGC	4	19965479	XPO1	11	3.417	2.952	4.992	X-RAY DIFFRACTION
XPO5	ENSG00000124571	no	Homo_sapiens	GUUU	4	19965479	XPO1	15	2.194	44077	1.898	X-RAY DIFFRACTION
ZFP36	ENSG00000128016	no	Homo_sapiens	CCCUCC	6	18367721	ZF_CCCH	5	5.132	5.729	6.041	RIP-chip
ZNF346	ENSG00000113761	no	Homo_sapiens	GUGGU	5	24521053	ZF_C2H2	36	4.396	4.307	4.398	SOLUTION NMR
RBMX	ENSG00000147274	no	Homo_sapiens	AAGU	4	12761049	RRM	34	2.484	2.589	2.279	Mutagenesis;Deletion analysis
SRSF1	ENSG00000136450	no	Homo_sapiens	CGCAGGA	7	11967553;12824367	RRM	28	9.471	30895	11.156	SELEX

* mutated target gene
