# Supplementary material for: New RNA Structural Elements Identified in the Coding Region of the Coxsackie B3 Virus Genome
Source: Viruses. 2020 Oct 30;12(11):1232. doi: 10.3390/v12111232 (PMC7692623; doi:10.3390/v12111232)
Supplement: Supplementary file 1 [file viruses-12-01232-s001.zip › SM5.pdf]

SM5-A Table. The top candidate proteins' lists. The MS data from the RNA-centric affinity chromatography experiment is included in the table. The table shows total numbers of MS matches/hits for each candidate protein as well as number of hits after correction with the control reaction (red font).

No	ID	Gene name	Protein name	MS spectra numbers		
				BEADS	F10RNA	F10RNA minus beads
1	P19338	**NCL**	Nucleolin	6	73	**67**
2	P09429	**HMGB1** HMG1	High mobility group protein B1	3	60	**57**
3	P61978	**HNRNPK** HNRPK	**Heterogeneous nuclear ribonucleoprotein K**	12	60	**48**
4	Q96AE4	**FUBP1**	Far upstream element-binding protein 1	5	53	**48**
5	Q92945	**KHSRP** FUBP2	**Far upstream element-binding protein 2**	1	46	**45**
6	P62280	**RPS11**	40S ribosomal protein S11	2	25	**23**
7	P62701	**RPS4X** CCG2, RPS4, SCAR	40S ribosomal protein S4, X isoform	3	23	**20**
8	P26373	**RPL13** BBC1, OK/SW-cl.46	60S ribosomal protein L13	1	19	**18**
9	P62269	**RPS18** D6S218E	40S ribosomal protein S18	4	21	**17**
10	P09651	**HNRNPA1** HNRPA1	**Heterogeneous nuclear ribonucleoprotein A1**	2	18	**16**
11	P62753	**RPS6** OK/SW-cl.2	40S ribosomal protein S6	2	18	**16**
12	P61247	**RPS3A** FTE1, MFTL	40S ribosomal protein S3a	1	17	**16**
13	P06748	**NPM1** NPM	Nucleophosmin	5	17	**12**
14	Q13283	**G3BP1** G3BP	Ras GTPase-activating protein-binding protein 1	2	14	**12**
15	Q13435	**SF3B2** SAP145	Splicing factor 3B subunit 2	1	13	**12**
16	P39019	**RPS19**	40S ribosomal protein S19	8	19	**11**
17	P26583	**HMGB2** HMG2	High mobility group protein B2	4	15	**11**
18	P68371	**TUBB4B** TUBB2C	Tubulin beta-4B chain	54	64	**10**
19	P23396	**RPS3** OK/SW-cl.26	40S ribosomal protein S3	6	16	**10**
20	P52597	**HNRNPF** HNRPF	**Heterogeneous nuclear ribonucleoprotein F**	2	12	**10**
21	P47914	**RPL29**	60S ribosomal protein L29	1	11	**10**
22	Q13765	**NACA** HSD48	Nascent polypeptide-associated complex subunit	1	10	**9**
23	E9PAV3	**NACA**	Nascent polypeptide-associated complex subunit	1	10	**9**
24	P11940	**PABPC1** PAB1, PABP1, PABPC2	**Polyadenylate-binding protein 1**	3	10	**7**
25	RS_P29353	**SHC1** SHC, SHCA	SHC-transforming protein 1	5	12	**7**
26	P31943	**HNRNPH1** HNRPH, HNRPH1	**Heterogeneous nuclear ribonucleoprotein H1**	2	8	**6**
27	P62241	**RPS8** OK/SW-cl.83	40S ribosomal protein S8	1	7	**6**
28	P62851	**RPS25**	40S ribosomal protein S25	1	7	**6**
29	P07814	**EPRS1** EPRS, GLNS, PARS, QARS,	Bifunctional glutamate/proline--tRNA ligase	2	8	**6**
30	P07437	**TUBB** TUBB5, OK/SW-cl.56	Tubulin beta chain	61	66	**5**
31	Q15366	**PCBP2**	**Poly(rC)-binding protein 2**	12	17	**5**
32	Q8NC51	**SERBP1** PAIRBP1, CGI-55	Plasminogen activator inhibitor 1 RNA-binding protein	5	10	**5**
33	Q13263	**TRIM28** KAP1, RNF96, TIF1B	Transcription intermediary factor 1-beta	6	11	**5**
34	P05198	**EIF2S1** EIF2A	Eukaryotic translation initiation factor 2 subunit 1	2	6	**4**
35	P62244	**RPS15A** OK/SW-cl.82	40S ribosomal protein S15a	2	6	**4**
36	P60866	**RPS20**	40S ribosomal protein S20	1	5	**4**
37	Q15643	**TRIP11** CEV14	Thyroid receptor-interacting protein 11	4	7	**3**
38	Q16777	**H2AC20** H2AFQ, HIST2H2AC	Histone H2A type 2-C	1	4	**3**
39	Q9BTM1	**H2AJ** H2AFJ	Histone H2AJ	1	4	**3**
40	Q6FI13	**H2AC18**, **H2AC19**	Histone H2A type 2-A	1	4	**3**
41	P0C0S8	**H2AC11, H2AC13, H2AC15,**	Histone H2A type 1	1	4	**3**
42	Q99878	**H2AC14** H2AFE, HIST1H2AJ	Histone H2A type 1-J	1	4	**3**
43	Q96KK5	**H2AC12** HIST1H2AH, HIST1H2AI	Histone H2A type 1-H	1	4	**3**
44	P20671	**H2AC7** H2AFG, HIST1H2AD	Histone H2A type 1-D	1	4	**3**
45	P68104	**EEF1A1** EEF1A, EF1A, LENG7	Elongation factor 1-alpha 1	11	13	**2**
46	Q5VTE0	**EEF1A1P5** EEF1AL3	Putative elongation factor 1-alpha-like 3	11	13	**2**
47	P46379	**BAG6** BAT3, G3	Large proline-rich protein BAG6	10	12	**2**
48	P67809	**YBX1** NSEP1, YB1	Nuclease-sensitive element-binding protein 1	7	9	**2**
49	P62829	**RPL23**	60S ribosomal protein L23	2	4	**2**
50	P60903	**S100A10** ANX2LG, CAL1L, CLP11	Protein S100-A10	1	3	**2**
51	P62266	**RPS23**	40S ribosomal protein S23	1	3	**2**
52	O75822	**EIF3J** EIF3S1, PRO0391	Eukaryotic translation initiation factor 3 subunit J	1	3	**2**
53	P42166	**TMPO** LAP2	Lamina-associated polypeptide 2, isoform alpha	1	3	**2**
54	Q9BQE3	**TUBA1C** TUBA6	Tubulin alpha-1C chain	37	38	**1**
55	Q71U36	**TUBA1A** TUBA3	Tubulin alpha-1A chain	37	38	**1**
56	P68363	**TUBA1B**	Tubulin alpha-1B chain	37	38	**1**
57	P52272	**HNRNPM** HNRPM, NAGR1	Heterogeneous nuclear ribonucleoprotein M	32	33	**1**
58	Q9H853	**TUBA4B** TUBA4	Putative tubulin-like protein alpha-4B	5	6	**1**
59	Q969Q0	**RPL36AL**	60S ribosomal protein L36a-like	7	8	**1**
60	Q14444	**CAPRIN1** GPIAP1, GPIP137,	Caprin-1	4	5	**1**
61	O75340	**PDCD6** ALG2	Programmed cell death protein 6	5	6	**1**
62	P49588	**AARS1** AARS	Alanine-tRNA ligase, cytoplasmic	4	5	**1**
63	P50991	**CCT4** CCTD, SRB	T-complex protein 1 subunit delta	4	5	**1**
64	P52943	**CRIP2** CRP2	Cysteine-rich protein 2	1	2	**1**
65	P08590	**MYL3**	Myosin light chain 3	1	2	**1**
66	P05976	**MYL1**	Myosin light chain 1/3, skeletal muscle isoform	1	2	**1**
67	P48643	**CCT5** CCTE, KIAA0098	T-complex protein 1 subunit epsilon	1	2	**1**
68	P51572	**BCAP31** BAP31, DXS1357E	B-cell receptor-associated protein 31	2	3	**1**
69	Q9Y224	**RTRAF** C14orf166, CGI-99	RNA transcription, translation and transport factor	1	2	**1**
70	Q96DB5	**RMDN1** FAM82B, CGI-90	Regulator of microtubule dynamics protein 1	1	2	**1**
71	P25398	**RPS12**	40S ribosomal protein S12	1	2	**1**
72	P49368	**CCT3** CCTG, TRIC5	T-complex protein 1 subunit gamma	1	2	**1**

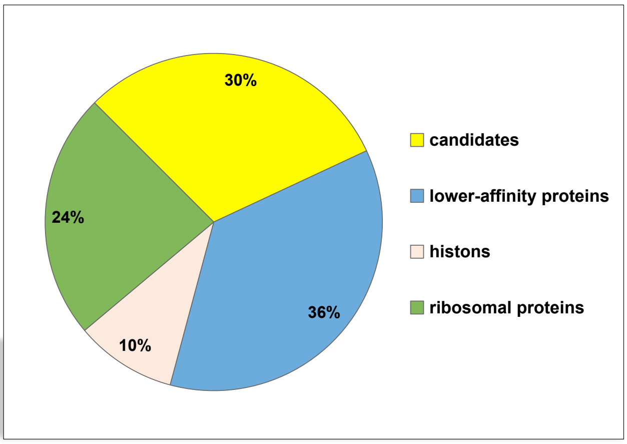

	number of proteins	%
candidate	22	30,55556
lower-affinity proteins	26	36,11111
histons	7	9,722222
ribosomal proteins	17	23,61111
total	72	100

SM5-B. Distribution of proteins identified by F10-CVB3 RNA affinity chromatography and MS analysis for untreated MCF-7 cells.

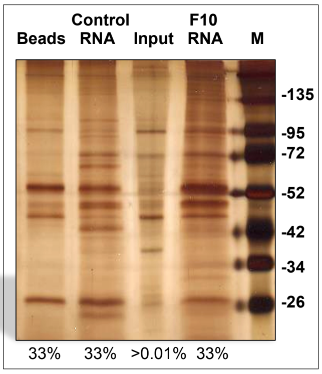

SM5-C. F10-CVB3 RNA affinity chromatography for untreated MCF- 7 cells. Total cytoplasmic fractions and the protein eluates were incubated for 5 min at 95°C and then loaded on a 10% SDS-PAGE gel. After electrophoresis the gel was silver stained according to manufacturer’s protocol (Pierce Silver Stain Kit).

Figure SM5. RNA affinity chromatography and MS analysis.
